# High-dimensional, outcome-dependent missing data problems: Models for the human KIR loci

**DOI:** 10.1177/09622802241304112

**Published:** 2025-01-31

**Authors:** Lars Leonardus Joannes van der Burg, Hein Putter, Henning Baldauf, Jürgen Sauter, Johannes Schetelig, Liesbeth C de Wreede, Stefan Böhringer

**Affiliations:** 1 Biomedical Data Sciences, 4501 LUMC, Leiden, The Netherlands; 2 535016 DKMS, Dresden/Tübingen, Germany; 3 Department of Internal Medicine I, University Hospital Carl Gustav Carus, Dresden, Germany; 4 Department of Pharmacology and Toxicology, 4501 LUMC, Leiden, The Netherlands

**Keywords:** Missing data, outcome dependent imputation, multiple imputation, expectation-maximization algorithm, haplotype reconstruction, *KIR* genes

## Abstract

Missing data problems are common in biological, high-dimensional data, where data can be partially or completely missing. Algorithms have been developed to reconstruct the missing values by means of imputation or expectation-maximization algorithms. For missing data problems, it has been suggested that the regression model of interest should be incorporated into the imputation procedure to reduce bias of the regression coefficients. We here consider a challenging missing data problem, where diplotypes of the *KIR* loci are to be reconstructed. These loci are difficult to genotype, resulting in ambiguous genotype calls. We extend a previously proposed expectation-maximization algorithm by incorporating a potentially high-dimensional regression model to model the outcome. Three strategies are evaluated: (1) only allelic predictors, (2) allelic predictors and forward-backward selection on haplotype predictors, and (3) penalized regression on a saturated model. In a simulation study, we compared these strategies with a baseline expectation-maximization algorithm without outcome model. For extreme choices of effect sizes and missingness levels, the outcome-based expectation-maximization algorithms outperformed the no-outcome expectation-maximization algorithm. However, in all other cases, the no-outcome expectation-maximization algorithm performed either superior or comparable to the three strategies, suggesting the outcome model can have a harmful effect. In a data analysis concerning death after allogeneic hematopoietic stem cell transplantation as a function of donor *KIR* genes, expectation-maximization algorithms with and without outcome showed very similar results. In conclusion, outcome based missing data models in the high-dimensional setting have to be used with care and are likely to lead to biased results.

## 1. Introduction

A common problem in the analysis of statistical models is the presence of missing data. This can be entirely missing values, that is, not available ( NA ) values, or partially missing data, where some information is still available. The latter case is termed coarsening, and includes missing phase information in genetic data.^1^

From a statistical point of view, missingness can be classified into the missing completely at random (MCAR), missing at random (MAR), and missing not at random (MNAR) taxonomy,^2^ which make different assumptions on the conditional distribution of the incomplete data, given observed data. MCAR assumes an independent distribution of missing values, making a complete case analysis valid, albeit power is usually reduced.^2,3^ The MAR assumption states that the probability of missingness only depends on other variables in the dataset. MAR underpins most approaches for handling missingness and under MAR complete case analysis can also lead to valid conclusions, although other analysis approaches are more efficient in general, because of the increased sample size.^4^ The discarding of incomplete cases can make the complete case analysis infeasible, especially for high-dimensional data where missingness is observed in most, if not all, participants. MNAR includes the case when unobserved variables are required for conditional independence and is difficult to deal with in practice.

For likelihood based models, two categories of approaches are often used, both assuming MAR: multiple imputation (MI)^2,5,6^ and expectation-maximization (EM) algorithms.^7^ Analysis using MI is a two-step procedure. In the first step, missing values are imputed by data drawn from an appropriate distribution iteratively, producing several datasets. In the second step, these data are analyzed “as usual” and results are pooled.^2,5,8^ The goal of MI is to derive valid pooled coefficients and standard errors in the second step.^2^ In contrast, the goal of the EM algorithm is to compute the maximum-likelihood estimate for each variable of the dataset. This is done by imputing expectations of missing values using working parameters assumed to approximate true parameters (the E-step). The MI and EM algorithm are similar in the sense that the same conditional distribution given observed data is used to draw from (MI) and to calculate expected values of (EM)*.* The resulting likelihood looks like a complete data likelihood and can be optimized as usual (the M-step).^9^ Resulting parameter estimates are used in an iteration as new working parameters.

Using MI, it is necessary to include the outcome variable of the regression model, that is the target of the analysis, in the procedure to avoid bias.^3,10^ This follows from the idea that if an association exists between predictors and outcome, their correlation is informative on missing values and if no such association exists, no harm is done. It has therefore been proposed to explicitly include the regression model into the MI procedure.^5^ The substantive model compatible fully conditional specification (SMC-FCS) procedure draws imputations by incorporating the regression model of interest into the imputation process, representing the research question. This model, also called the substantive model, models the expected relationship between predictors and outcome. The SMC-FCS model is a principled approach that leads to unbiased parameter estimates under non-linear link-functions while methods that ignore the substantive model can lead to biased estimates.^5^ This holds under correct specification of the substantive model. We here define a substantive model to be designed to answer the underlying research question and to be used after the imputation procedure has been completed. Such a substantive model might be difficult to define before imputation, because of issues like model selection and regularization. We distinguish here between the substantive model to be used *after* imputation, and the outcome model to be used *during* imputation, which is a regression model with the same outcome as the substantive model, but using fewer biological or statistical assumptions or chosen by statistical convenience. When emphasizing this distinction we refer to the latter as *working outcome model*. Considering the fact that it is common practice to include more variables into an MI procedure than are used in a substantive model in order to improve imputation results,^10^ a mismatch between the substantive and working outcome models occurs regularly in practice. As depending on the research question a substantive model might be hard to define, discrepancies between substantive and outcome models are important to investigate. We do this by mimicking several practical approaches covering the high-dimensional setting.

To this end, we develop an EM algorithm including an outcome model for a specific genetic situation which should, under correct specification, allow for unbiased estimation of the substantive model. To understand characteristics of the procedures we investigate two properties: first, the distribution of the partly missing data in terms of the overall distribution conditioned on all observed covariates and the distribution per individual, as proper imputation of values is the first step in obtaining optimal regression coefficients. Second, the bias and efficiency of the regression coefficients of a substantive model is of interest.

We have chosen the problem of diplotype (pair of haplotypes) reconstruction for ambiguous genotype data^11^ and discuss broad applicability of our results below. Previous work suggests that results can be improved when outcome data is taken into account beyond genetic data alone.^11^ However, this comes with several challenges. First, as discussed above, it is not straightforward to specify a substantive model. In general, most genetic variants are considered to have a small effect on any outcome^12,13^ implying that the correct choice of predictor variables is unknown a-priori. Second, potential outcome models are usually high-dimensional as haplotype (tuples of alleles) based models are of interest, eventually leading to fully saturated regression models. Third, we want to allow an analyst to specify a very rich outcome model, possibly including additional covariates that help to impute but are not of direct relevance for the substantive model. After the imputation procedure, the analyst can then fit and test several substantive models. This implies that a regression model used after running the EM algorithm might differ from the outcome model during the algorithm. Although this approach slightly deviates from the idea that the imputation model should be compatible with the substantive model, it leads to a much less cumbersome and slow workflow compared to re-running every new substantive model with the EM algorithm. This also reflects common practice, when no substantive model can be specified a-priori.

Our work is motivated by studies of the killer-cell immunoglobulin-like receptor (*KIR*) gene region, which is one of the most complex genetic regions observed in humans exhibiting high allelic polymorphism, copy-number-variation (CNV), and allele homology across genes, thereby making it difficult to measure and analyze. We illustrate our methods on a dataset describing the impact of *KIR* genes on outcomes after allogeneic stem cell transplantation of patients with secondary acute myeloid leukemia (sAML) or myelodysplastic syndromes (MDS).^11,14^ One main source of missingness in this data stems from the measurement process and leads to ambiguity of genotype calls, that is, a measurement is, in general, compatible with a larger set of diplotypes.^15^ Due to the high amount of missing data in this application, it is paramount to retain as much information as possible, suggesting to add an outcome model to the EM algorithm, to improve both estimation of haplotype frequencies (HTFs) and of regression coefficients that capture the impact of *KIR* genes on outcomes after transplantation.

*KIR* genes are under investigation because they orchestrate natural-killer (NK)-cell functioning, which is assumed to play a role in the graft-versus-leukemia (GvL) reaction after allogeneic hematopoietic stem cell transplantation (alloHCT). Certain *KIR* variants have previously been found to be associated with reduced disease relapse and improved patients’ overall survival, although their role is still under scrutiny.^14,16–18^ To fit in the methodological framework of the current article focusing on parametric regression models, we present a much simplified version of the analyses shown elsewhere by modeling patient death until 6 months after alloHCT as a binary outcome in our data application.

In a more general setting, we consider high-dimensional data for which a substantial model is either unknown or difficult to fit. While we consider a specific example concerning genetics, we believe that our findings are valid for similar data. Dimensionality of the data considered in this study is comparable to many other research questions (omics data such as gene expression, DNA variation, and blood metabolites). In our case, we can handle missing data using a likelihood approach, which might not be possible in other situations, making our results optimistic. The reader more interested in general conclusions can therefore focus on simulation results that give an impression on problems arising from similar amount of missingness in other datasets.

The article is structured as follows. In Section 2, we develop an EM algorithms for diplotype reconstruction taking into account an outcome model, and describe methodology used in the simulations and data analysis. Section 3 describes an extensive simulation study. In Section 4, we present results from the simulation study and a real data analysis and we close with a discussion in Section 5.

## 2. Methods

### 2.1. Data and genetic likelihood

Genetic data considered here is composed of observations of DNA sequences at fixed positions (loci), called alleles. Tuples along a single chromosome of alleles form haplotypes. Pairs thereof, inherited from both parents, are called diplotypes. If pairs of alleles are observed per locus, that is, it is unknown on which chromosomes alleles lie, observations are called (unphased) genotypes. The missing data problem considered here is that allelic state is observed incompletely and that phasing information is missing in general. Additionally, several loci might be indistinguishable by the measuring process, resulting in more than two observed alleles per locus (copy-number variation).^19^ We first consider genetic information only. Following notation from van der Burg et al.,^11^ for L loci, denote with $G_{i}=(G_{i1},\ldots ,G_{iL})$ observed genotypes for individual i . These genotypes may be ambiguous, that is, each genotype $g_{i}$ represents a set of possible underlying true diplotypes, denoted with $H_{i}=\{H_{i1},H_{i2}\}$ . Diplotypes are unobserved and we use notation $\{h_{1}|h_{2}\}∼g_{i}$ for the set of diplotypes compatible with a genotype observation. To guarantee the MAR setting, we assume that the conditional distribution $P(H_{i}|g_{i})$ is identical for all true, underlying diplotypes compatible with $g_{i}$ . Additionally assuming Hardy-Weinberg equilibrium (which assumes independence of haplotypes within diplotypes), estimates for HTFs, which are denoted by π are defined as the arg-max of the observed data likelihood,

(1) $$L_{\text{g, obs}}(\pi )=\prod_{i=1}^{N}\left(\sum_{\{h_{1}|h_{2}\}∼g_{i}}\pi_{h_{1}}\pi_{h_{2}}c_{h}\right)$$

where $\pi =(\pi_{1},\ldots ,\pi_{M})$ with M the number of haplotypes and N is the number of individuals in the dataset. Here, $c_{h}$ is a constant representing heterozygosity ( $c_{h}=2$ for a heterozygote diplotype and 1 otherwise). For complete data, the likelihood simplifies to

(2) $$L_{g}(\pi ;H)=\prod_{i=1}^{N}\pi_{h_{i1}}\pi_{h_{i2}}c_{hi}$$

where $H=(H_{1},\ldots ,H_{N})$ is the vector of diplotypes with $H_{i}=(H_{i1},H_{i2})$ . The logarithm of $L_{g}(\pi ;H)$ , denoted by $\ell_{g}(\pi ;H)$ , can be rearranged as follows:

(3) $$\ell_{g}(\pi ;H)=\sum_{i=1}^{N}\log\;\left(\pi_{h_{i1}}\pi_{h_{i2}}c_{hi}\right)=\sum_{j=1}^{M}\log\;(\pi_{j})\sum_{i=1}^{N}I_{ij}$$

with $I_{ij}=I(h_{i1}=j)+I(h_{i2}=j)$ , see also van der Burg et al.^11^ In case of missing data, ML estimates can also be obtained by the profile EM algorithm using the expected complete data likelihood.^11^

### 2.2. Likelihood with outcome

We now assume that besides a multi-locus genotype also an outcome is observed, depending on the diplotype and potentially influenced by additional covariates. We assume that the outcome is modeled by a regression model, the density of which is given by $L_{o}$ , and that all additional covariates are fully observed. This outcome likelihood can be incorporated into the EM algorithm by factoring the joint complete data likelihood L(θ;Y,H) as $L_{o}(\theta ;Y|H)L(\theta ;H)$ :

(4) $$L(\theta ;Y,H)=\prod_{i=1}^{N}L_{o}(\tau ;Y_{i}|h_{i})\underset{L_{g}(\pi ;H_{i})}{\underbrace{\pi_{h_{i1}}\pi_{h_{i2}}c_{hi}}}$$

where $Y_{i}$ is the outcome of individual i and θ=(π,τ) is the parameter vector. τ is the parameter vector of the outcome likelihood and is composed of regression coefficients and potentially scaling parameters. Making an additional assumption about independence of nuisance covariates and diplotypes, additional covariates can be used in the outcome likelihood. For brevity, we omit covariates in the following. The log-likelihood then becomes

(5) $$\ell (\theta ):=\ell (\theta ;Y,H)=C+\ell_{g}(\pi ;H)+\sum_{i=1}^{N}\ell_{o}(\tau ;y_{i},h_{i})$$

Here $\ell_{o}$ is the log of $L_{o}$ , and $C=\sum_{i}\log\;(c_{hi})$ .

### 2.3. EM algorithm

Each EM algorithm starts with the full data likelihood, which has to iterate/integrate over all possibilities for missing values. This likelihood can then be used to condition missing values on observed values and compute the expectation under given parameter values.^7^ In the context of genetics, mostly phase-ambiguity (i.e. genotypes are observed, but diplotypes are of interest) has been considered.^20,21^ Our study is based on an extended model for HTF estimation^11^ that can cope with genetically diverse gene regions as well as with genotyping ambiguities. First, both ambiguous genotype calls and CNVs are allowed. Second, the algorithm controls complexity by an iterative reconstruction and collapsing of rare haplotypes. Third, the stability of HTF estimates is guaranteed by a profiling step.

Lacking an analytical solution for the maximum likelihood estimator (MLE), the log-likelihood is optimized using an EM algorithm which iterates between taking expectations (E-step), and updating parameters $\theta^{\left(k\right)}$ in the M-step.^7^

#### 2.3.1. E-step

Denote with $\theta^{\left(k\right)}=(\tau^{\left(k\right)},\pi^{\left(k\right)})$ the current parameter estimates. Expectations are taken with respect to the conditional distribution

(6) $$P_{\theta^{\left(k\right)}}^{\left(i\right)}\left(H_{i}=(h_{i1},h_{i2})|G_{i},Y_{i}\right)=\frac{P(Y_{i}|H_{i}=(h_{i1},h_{i2}))P(H_{i}=(h_{i1},h_{i2}))}{\sum_{j\in H_{i}}P(Y_{i}|H_{i}=j)P(H_{i}=j)}$$

Here $H_{i}=\{h_{1}|h_{2}\}∼g_{i}$ . Using independence of the observations i=1,…,N , $P_{\theta^{\left(k\right)}}=\prod_{i=1}^{N}P_{\theta^{\left(k\right)}}^{\left(i\right)}$ denotes the resulting product measure. Taking the conditional expectation of the complete data log-likelihood with respect to $P_{\theta^{\left(k\right)}}$ leads to

(7) $$\begin{array}{rl} l_{E}(\theta ;G,Y) & =C+E_{P_{\theta^{\left(k\right)}}}\left[\sum_{j=1}^{M}\log\;(\pi_{j})\sum_{i=1}^{N}I_{ij}\right]+E_{E_{P_{\theta^{\left(k\right)}}}}\left[\sum_{i=1}^{N}l_{o}(\tau ;y_{i},h_{i})\right] \end{array}$$

(8) $$\begin{array}{rl} & =C+\sum_{j=1}^{M}\log\;(\pi_{j})\sum_{i=1}^{N}\underset{A_{ij}}{\underbrace{E_{P_{\theta^{\left(k\right)}}^{\left(i\right)}}\left[I_{ij}\right]}}+\sum_{i=1}^{N}\underset{B_{i}}{\underbrace{E_{P_{\theta^{\left(k\right)}}^{\left(i\right)}}\left[l_{o}(\tau ;y_{i},h_{i})\right]}} \end{array}$$

Terms $A_{ij}$ are computed directly as sums of probabilities given in equation (6). It remains to compute $B_{i}$ . To this end, we assume $l_{o}$ to come from an exponential family of distributions. All distributions from this family, like the normal and binomial, can be written in the common linear-exponential form of $f_{Y}(y,\delta ,\phi )=\exp\;\{y\delta -b(\delta ))/a(\phi )+c(y,\phi )\}$ , where a() , b() , and c() are known functions, δ the canonical parameter, and ϕ the dispersion parameter.^22^ This is linked with equation (8) via τ=(δ,ϕ) and $\delta_{i}=g(x_{i}\beta )$ , the linear prediction with link function g() . This allows to assume the following form for $E_{P_{\theta^{\left(k\right)}}}(l_{\phi })$ :

(9) $$\begin{array}{rl} E_{P_{\theta^{\left(k\right)}}}\left[l_{\phi }(\tau ;y,h)\right] & =E_{P_{\theta^{\left(k\right)}}}\left[\sum_{i=1}^{N}\left\{\log\;(e(h_{i}))+\log\;(g(\tau ))+\eta (\tau {)}^{T}T(h_{i})\right\}\right] \end{array}$$

(10) $$\begin{array}{rl} & =\sum_{i=1}^{N}\left[E_{P_{\theta^{\left(k\right)}}}(\log\;(e(h_{i})))+\log\;(g(\tau ))+\eta (\tau {)}^{T}E_{P_{\theta^{\left(k\right)}}}(T(h_{i}))\right] \end{array}$$

(11) $$\begin{array}{rl} & =\sum_{i=1}^{N}\left[D_{i}+\log\;(g(\tau ))+\eta (\tau {)}^{T}\hat{T}(h_{i})\right] \end{array}$$

Here e and g are real-valued, η and T are vector-valued functions. The first expectation in equation (10) does not depend on parameters and can be treated as constant ( $D_{i}$ ). We compute this expectation using weighted regression, where each individual is entered multiple times into the dataset, once for each compatible diplotype with each row being weighted by the corresponding diplotype probability.^22^

#### 2.3.2. M-step

The M-step is estimated as the population frequency, calculated via $\frac{1}{2N}\sum_{i=1}^{N}x_{ij}$ , where $x_{ij}$ is the combination of $A_{ij}$ and $B_{i}$ (equation (8)).^11^

### 2.4. Reconstruction algorithm

**Figure 1.**
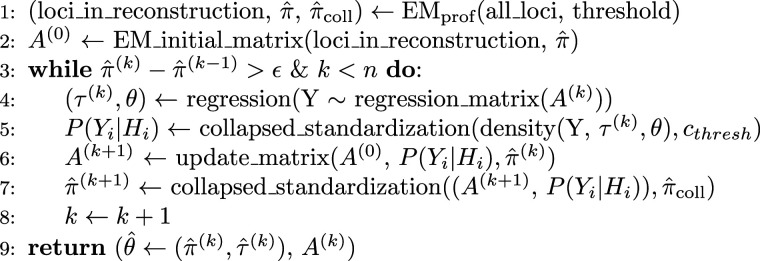
Profile EM-algorithm with outcome for a set of loci. HTFs are updated with the profile EM-algorithm with outcome for the haplotypes (*loci in reconstruction*) by iterating between a weighted haplotype only profile EMalgorithm (EM_prof_; van der Burg et al. (2023)) and updating regression coefficients. Maximized parameters are returned as θ .

To reconstruct actual data, the EM algorithm has to be broken down into smaller steps to make it computationally feasible (Figure 1). The algorithm adds loci iteratively, each time adding a new locus to a previous reconstruction. To obtain good starting values, the algorithm starts with the profile EM algorithm not including outcome information (step 1; van der Burg et al.^11^) results of which are used for the first E-step (matrix $A^{\left(0\right)}$ , step 2). After fitting the working outcome model and obtaining regression coefficients as the first component of the parameter vector (M-step, step 4), the E-step is performed, updating the design matrix using this model (step 6; equation (6)). Finally, HTFs are updated, completing the E-step (step 7). In this step, rare haplotypes with frequency estimates below a threshold are collapsed into a synthetic “collapsed” haplotype. The EM algorithm is continued until convergence or exceeding an iteration threshold. Unless stated otherwise, iteration and collapsing thresholds are chosen as 200 and $1\times 10^{-7}$ , respectively. Convergence is defined as largest change in HTFs of subsequent EM algorithm iterations of $<10^{-5}$ .

Note that in the E-step for HTFs (step 7), frequencies of collapsed haplotypes from the previous step are fixed to their frequency from the previous iteration, resulting in a profiling for this parameter. This profiling was introduced earlier and is due to the fact that collapsing does not retain full information about missingness and can, therefore, lead to bias (see van der Burg et al.^11^ for details).To avoid numerical instability, the terms $P(Y_{i}|H_{i})$ are rescaled (step 5) as all these terms might be close to zero. To this end, they are rescaled to a constant $c_{thresh}$ separately for collapsed and non-collapsed haplotypes. This avoids the situation when all probability mass comes to lie on a collapsed haplotype. Full details are given in Supplemental Appendix A.

#### 2.4.1. Outcome design matrix

Different types of design matrices are used depending on their role in the procedure: data simulation, the different iterations of the EM algorithm and a substantive model for data analysis, all potentially being different. All matrices are derived by the following steps, starting with the matrix of known (simulation) or expected (EM algorithm or substantive model) diplotype counts $\left(A_{ij}\right)$ . This matrix is transformed into main effects and higher-order terms through marginalization and an intercept column is added. The new matrix contains (expected) number of alleles, pair-wise haplotypes, three-locus haplotypes, etc. in additive coding. To ensure full rank, the “NEG” allele, denoting the absence of a genotype call, is chosen as a reference allele, implying that alleles and haplotypes containing this allele are excluded from the design matrix. This matrix is saturated and depending on its role, columns are removed.

#### 2.4.2. Working outcome model

Since in a high-dimensional situation it is generally difficult to specify a “true” regression model additional modeling steps are required. Three strategies for constructing the design matrix for the outcome model within the EM algorithm (step 4) were considered:

1. **Allele effects**: All pair-wise and higher-order haplotypes are discarded from the design matrix; CNVs contribute additively to the constituting alleles.
2. **Forward-backward selection**: Analysis starts with allele main effects from strategy 1. Next, pair-wise haplotypes consisting of significant alleles ( p -value <0.1 ) are included in a forward step, where-upon haplotypes are eliminated using backward-selection ( p -value >0.1 ). If any significant pair-wise haplotypes are retained, a forward-backward step for three-locus haplotypes is performed, etc. When no haplotypes are selected, the approach is identical to the first strategy.
3. **Penalized regression**: The full design matrix is entered into an elastic net regression. A tuning parameter λ is selected by cross-validation for mixing parameter α=0.5 (*R* package *glmnet*^23^). Penalized regression is a likelihood based approach that adds a penalty term that is a function of the model parameters and parameterized by a tuning parameter. These models include the LASSO, elastic net, and ridge regression.^24^ These methods can be used in case the design matrix in undetermined, that is, in the high-dimensional case.

#### 2.4.3. CNV alleles

CNV observations are sets of alleles that can be observed for a gene on a single haplotype, molecularly corresponding to several gene copies. In the simulation design matrix, the set of alleles is treated as a separate allele observable for this gene (locus). Because of their low frequency, effect sizes are difficult to estimate and we decompose CNV alleles into their individual alleles, when constructing design matrices for model fitting. CNVs contribute to each allele or haplotype in the allele set as a fractional count (additive effect), which is determined as one over the number of combinations that can be formed by the decomposition.

#### 2.4.4. Substantive models

A design matrix is constructed based on the expected counts as described above (Section 2.4.1). Two types of substantive models are considered: (1) only use allelic predictors, (2) use all haplotypes with an expected frequency >0.01 (the so-called candidate list haplotypes) ensuring identical design matrices across iterations (only possible in simulations or if other information is available to build this list). This latter model requires a penalized approach with elastic net penalty for the analysis, where the tuning parameter is again selected by cross-validation.

## 3. Simulation study

Our simulation study follows the aims, data-generating mechanisms, estimands, methods, and performance measures (ADEMP) structure discussed by Morris et al.^25^

### 3.1. Aim

The aim of the simulation study is to compare the performance of the profile EM algorithm based on genotypes only with the profile EM algorithm with outcome. As outcome models, the different modeling strategies mentioned above (Section 2.4.2) are compared.

### 3.2. Data-generation mechanism

#### 3.2.1. Haplotype simulation

Diplotypes consisting of three loci were simulated. We used N=2000 individuals throughout, roughly corresponding to the sample size of the dataset. Two different sets of values of allele frequencies (AFs) per locus and several values for the correlation between alleles (linkage disequilibrium; LD) were considered. In the KIR dataset, there are roughly speaking two different allele distributions observed. Some genes have one allele with a high frequency ( >0.7 ; often the “NEG” allele, i.e. the absence of the locus in the individual), while the other genes exhibit a more equally divided distribution (i.e. all AFs <0.5 ). For more information about the KIR dataset, see van der Burg et al.^11^ The AFs used for the three genes in the two simulation sets are given in Table 1.

**Table 1.** Parameters of the simulation studies.

Gene 1		Gene 2		Gene 3	
NEG1	0.15/0.15 (0.00/0.00)	NEG2	0.31/0.31 (0.00/0.00)	NEG3	0.70/0.70 (0.00/0.00)
A ${}^{⋆}$	0.40/0.20 (0.75/0.75)	E	0.43/0.32 ( − 0.50/ − 0.50)	G	0.19/0.125 (0.25/0.25)
B	0.30/0.20 (0.25/0.25)	F ${}^{⋆}$	0.25/0.32 (0.50/0.50)	H ${}^{⋄}$	0.10/0.125 ( − 0.50/ − 0.50)
C ${}^{⋄}$	0.10/0.20 ( − 0.75/ − 0.75)	E $\hat{\;}$ F	0.01/0.05 (0.00/ − 0.50)	G $\hat{\;}$ H	0.01/0.05 ( − 0.25/ − 0.50)
D	0.04/0.20 ( − 0.25/ − 0.25)				
A $\hat{\;}$ B	0.008/0.04 (1.00/2.00)				
C $\hat{\;}$ D	0.002/0.01 ( − 1.00/ − 2.00)				

Note: The two sets of AFs (set 1/set 2) for the simulation of the individual diplotypes. AFs are based on the actual KIR dataset. Within brackets, the starting points of the two sets of regression coefficients (set 1/set 2) for the simulation of the outcomes. These effect sizes are divided or multiplied by 5, thus each set leads to three sub-scenarios. Haplotypes that in the scenarios with haplotype effects have a double effect (once for individual alleles and once for the haplotype) are A–F and C–H (also indicated by superscripts ⋆ and ⋄ ). LD is added to the combinations B–F, D–G, and E–H, where allelic ambiguities are added between the haplotypes A/C, B/D, E/F, and G/H. Alleles consisting of two gene copies (copy number variation) are separated by a caret ( $\hat{\;}$ ). These simulation studies are similar to simulation study I by van der Burg et al.,^11^ but have different effect sizes.

AFs: allele frequencies; KIR: killer-cell immunoglobulin-like receptor; LD: linkage disequilibrium.

For diplotype simulation, HTFs are determined first. To this end, loci are added iteratively, where in each iteration, LD between new alleles and existing haplotypes defines the new joint, multinomial distribution (Table 1). From this distribution, $2n_{obs}$ haplotypes are drawn. After converting diplotypes to genotypes, ambiguities are added to all allele copies in eligible genotype calls with constant probabilities across loci based on varying ambiguity scenarios (Table 1). For all such allele copies resulting in the same ambiguous genotype, the same probability of being ambiguous is used, corresponding to the MAR assumption mentioned above. Note that, in each scenario, there is always some base level of ambiguity, which originates from haplotype phasing and CNVs. Both for LD and ambiguities we define three levels: none ( 0 ), medium ( 0.4 ), and high ( 0.8 ) on a standardized scale between 0 and 1 (see Supplemental Appendix B).

#### 3.2.2. Outcome simulation

To determine the effect of ambiguities on estimating allele and haplotype effects in regression models, continuous and binary outcomes were simulated based on diplotypes. The design matrix X is constructed as described above (Section 2.4.1). Both allele and haplotype effects were considered (Table 1). Continuous outcomes are drawn according to the following model:

(12) $$Y=X^{⊺}\beta +\epsilon$$

where $\beta =(\beta_{0},\ldots ,\beta_{A})$ , with intercept $\beta_{0}=0$ , and $\epsilon_{i}\overset{i.i.d.}{∼}N(0,1)$ . Binary outcomes are drawn from independent Bernoulli variables with probabilities

(13) $$P(Y=1)=(1+\text{exp}(-X^{⊺}\beta ){)}^{-1}$$

Here $\beta_{0}=-1.75$ , which corresponds to a baseline event probability of 15%. Regression coefficients for the different simulation scenarios are given in Table 1. Haplotype effects, if present, were chosen as the sum of the effects of its alleles.

#### 3.2.3. Partial factorial design

To limit the number of simulation scenarios, a subset was chosen from combinations of (1) two sets of HTFs (non-uniform (default) and approximately uniform), (2) two sets of regression coefficients (small CNV allele effects (default) and strong CNV allele effects), (3) two sets of (non-allele) haplotype regression coefficients (none (default) and haplotype effects present), and (4) two types of outcomes (continuous (default) and binary), leading to a partial factorial design of eight scenarios (Supplemental Appendix C and Table S1). Criteria to eliminate scenarios were to avoid models with likely unstable outcome models (e.g. small effect sizes and low corresponding HTFs). Each of these scenarios was combined with LD ( N=3 ), ambiguity ( N=3 ), within EM algorithm outcome models ( N=4 ; without outcome, with allelic outcome; with forward-backward selection outcome or with penalized outcome), and sub-scenarios ( N=3 ; default effect sizes or divided by or multiplied with (5), leading to 864 scenarios in total.

#### 3.2.4. Proof-of-concept simulations

To establish validity of the outcome profile EM algorithm, another two simulation scenarios with few alleles and strong effect sizes were chosen, without (scenario A) and with (scenario B) haplotype effects (Table 2). Simulations for this scenario follow the same steps as described above.

**Table 2.** Parameters of the proof-of-concept simulation.

Gene 1		Gene 2		Gene 3	
NEG1	0.25 (0.00)	NEG2	0.35 (0.00)	NEG3	0.70 (0.00)
A ${}^{⋆}$	0.45 (5.00)	E ${}^{⋆}$	0.35 (3.50)	G	0.15 (2.50)
B	0.30 ( − 5.00)	F ${}^{⋄}$	0.30 ( − 3.50)	H ${}^{⋄}$	0.15 ( − 2.50)

Note: The set of AFs for the simulation of the individual diplotypes. AFs are based on the actual KIR dataset. Within brackets, the starting points of the effects size set for the simulation of the individuals outcome. These effect sizes are divided or multiplied by 3, thus each set leads to three sub-scenarios. Haplotypes that in the scenarios with haplotype effects have a double effect (once for individual alleles and once for the haplotype) are A–E and F–H (also indicated by superscripts ⋆ and ⋄ ). LD is added to the combinations A–F, B–G, and E–H, where allelic ambiguities are added between the haplotypes A/B, E/F, and G/H.

AFs: allele frequencies; KIR: killer-cell immunoglobulin-like receptor; LD: linkage disequilibrium.

### 3.3. Estimands

Estimands are the individual and population HTFs. The population HTFs are the ${\hat{\pi }}^{\left(k\right)}$ estimated in the outcome EM algorithm (Figure 1). We define individual HTFs by converting expectations estimated by the EM algorithm ( $A_{ij}^{\left(k\right)}$ from iteration k ) which are counts of haplotypes to probabilities of diplotypes for each individual. These vectors can be compared to the vector where probability of one is assigned to the true diplotype. The average of individual HTFs are population HTFs. Accuracy of these estimands can differ within a single dataset, for example, when ambiguities are focused on less frequent haplotypes making diplotype predictions for carriers difficult, without substantially affecting overall accuracy.

Additionally, we evaluate the accuracy of substantive model regression coefficients.

### 3.4. Methods to evaluate

Each replication is analyzed with the profile EM algorithm with and without included outcome. The threshold for collapsing rare haplotypes was set to $1\times 10^{-7}$ , as motivated by previous work.^11^ To facilitate valid comparisons between all replications, the order in which genes were added into the reconstruction was fixed as given in Table 1. Additionally, to ensure that the effect sizes between the replications can be compared, all haplotypes with a theoretical probability >0.01 (candidate list haplotypes) were never collapsed into the collapsed haplotype, even if their frequency was estimated to be under the threshold value. As part of model evaluation, we also compare the different model selection strategies (alleles only, forward-backward and penalized) with the no-outcome EM algorithm and among each other.

### 3.5. Performance measures

For each scenario, 100 independent replications ( $n_{sim}$ ) were run (for motivation of this number, see van der Burg et al.^11^). Quality of the estimates obtained from the different methods in the different scenarios is assessed via the root mean square error (RMSE), incorporating both the bias and the variance of the estimates

(14) $$\text{RMSE}=\sqrt{\frac{1}{n_{sim}}\sum_{i=1}^{n_{sim}}{\left(Z_{i}-{\hat{Z}}_{i}\right)}^{2}}$$

where $Z_{i}$ and ${\hat{Z}}_{i}$ are the true and estimated value, respectively, of the HTFs and the regression coefficients of both the outcome and substantive models. The estimated HTFs are further analyzed with the haplotype reconstruction (HTR) measure (equation (15)) and the Kullback-Leibler divergence (KLD),^11^ where low RMSE, HTR, and KLD values correspond to better diplotype reconstruction.

To quantify individual HTF accuracy, we use the HTR measure:

(15) $${\text{HTR}}^{\text{DT}}=\frac{1}{N}\sum_{j=1}^{N}{\left({\hat{\pi }}_{ij}-\pi_{ij}^{c}\right)}^{2}$$

where for individual i , ${\hat{\pi }}_{ij}$ is the estimated allele frequency vector for locus j and $\pi_{ij}^{c}$ the true probability vector for locus j . This HTR measure only penalizes differences in diplotypes when their allele composition is different, not when differences are due to haplotype phasing. CNVs are decomposed into alleles for this measure.

## 4. Results

### 4.1. Proof-of-concept

We start with a simple proof-of-concept simulation study, including few allele effects. Here, only the sub-scenario of scenario A (without additional haplotype effects) containing high effect sizes will be discussed. We only show results for the six HTs with the highest HTFs*.* Further explanation and results are given in Supplemental Appendices D and E.

**Figure 2.**
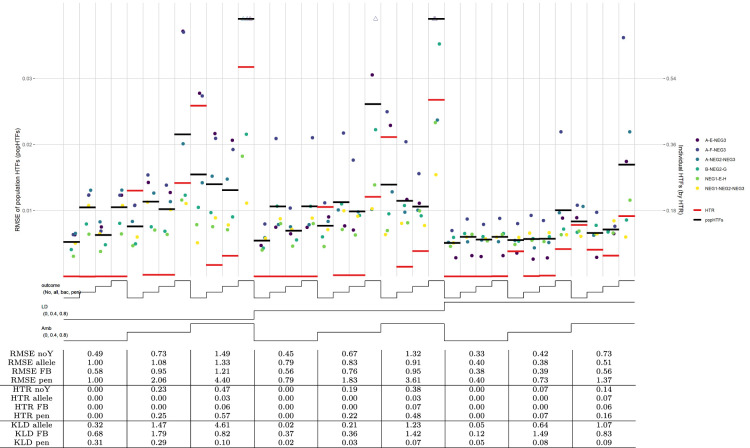
HTF estimation, scenario A. Note: Nested-loop plot of RMSEs for HTFs and HTR measure for all sub-scenarios of the proof-of-concept study without haplotype effects. Each column of points separated by vertical grid lines depicts a different set of parameters, where the lines at the bottom of the graph define these parameter sets. Each sub-scenario is analyzed by four outcome models: no-outcome (lowest line; noY), allelic model (one step; allele), forward-backward model (second step; FB), and penalized regression model (upper line; pen). The sub-scenarios differ in their added LD and ambiguities, where a step in the line corresponds to a higher value (lowest line is 0.0; highest line is 0.8). Further explanation of the nested-loop plot is given by Rücker and Schwarzer.*^26^* The haplotypes plotted are a combination of gene 1, gene 2, and gene 3, with the genes in that order separated by a “ − .” For visualization purposes, dots of haplotypes with a too high RMSE are replaced by a triangle and put on top of the graph. The horizontal bold black lines represent the RMSE of the population HTFs (popHTFs; with values on the left *y*-axis), while the bold red lines represent the HTR measure of the individual HTFs (with values on the right *y*-axis). The table below the graph shows mean RMSE values of these popHTFs and the HTR measures, as well as the Kullback-Leibler divergence ratios between the no-outcome model and the three outcome models. All values in the table give ranges (min–max) of values for the three sub-scenarios above. A ratio >1 indicates higher similarity of the HTFs of the outcome models with the truth than the no-outcome model. Mean RMSE values in the table have been multiplied by 100 for readability. In this plot, only the six HTs with the highest HTFs are plotted. The mean RMSE, HTR measure and table are based on all HTs, which are also displayed in Supplemental Figure S1.

The simulation results are displayed in a nested-loop plot of Figure 2*. *In this plot, results of all sub-scenarios are plotted in vertical bins stacked next to each other, where the set of lines below the graph indicates the parameters used for each sub-scenario. For scenario A, there are nine datasets with different combinations of LD and ambiguities, and each dataset has been analysed with four EM algorithms that differ in the used outcome model: no-outcome model, allelic model, forward-backward model, and penalized model. The HTFs estimated with each model are compared, both on an individual level with the HTR measure (bold red horizontal lines), and on the population level with population HTFs (bold black horizontal lines).

As observed by the large differences between the red lines corresponding to the HTR measure, HTFs estimated with the allelic outcome model strongly outperform the no-outcome model in sub-scenarios with medium and/or high ambiguity and LD (Figure 2). The forward-backward selection model performs slightly worse than the allelic model, but still better than the no-outcome model, while the penalized model is slightly worse than the no-outcome model. Both the forward-backward and penalized models take possible haplotype effects into account, which were absent in the simulations. This is also reflected in KLD ratios (comparing the KLD of outcome models with the KLD of the no-outcome model), which for most of these sub-scenarios are above one (Figure 2). However, the fact that the black lines corresponding to the population HTFs are comparable across most sub-scenarios shows that the beneficial effect of the allelic and forward-backward model in the aforementioned sub-scenarios is small. This is especially true for sub-scenarios with low ambiguities, which is further confirmed by KLD ratios being far below 1.

Two substantive models were evaluated: one with only allelic predictors and another with all candidate list haplotypes. RMSEs for the regression coefficients from both substantive models follow a similar pattern as observed above: high effect sizes allow for more reliable estimation with the allelic and forward-backward selection model (Supplemental Figure S2(A) and (B)). For other sub-scenarios, the four methods perform comparably.

### 4.2. Partial factorial design

#### 4.2.1. Haplotype frequencies

**Figure 3.**
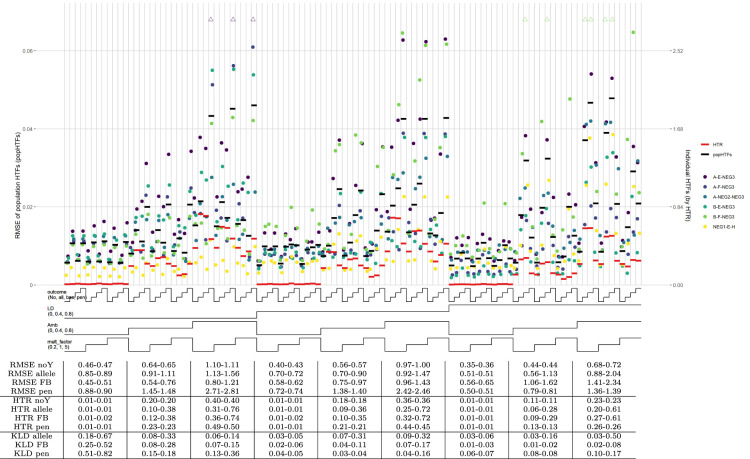
HTF estimation, scenario 001. Note: Nested-loop plot of RMSEs for HTFs and HTR measure for all sub-scenarios of default scenario 001, containing HTF and effect size set 1. Explanation about the nested-loop plots is given in the legend of Figure 1. The horizontal bold black lines represent the RMSE of the popHTFs (with values on the left *y*-axis), while the bold red lines represent the HTR measure of the individual HTFs (with values on the right *y*-axis). The table below the graph shows mean RMSE values of these popHTFs and the HTR measures, as well as the KLD ratios between the no-outcome model and the three outcome models. All values in the table are the range (min–max) of values for the three sub-scenarios above. A ratio >1 indicates higher similarity of the HTFs of the outcome models with the truth than the no-outcome model. Mean RMSE values in the table have been multiplied by 100 for readability. In this plot only, the six HTs with the highest HTFs are plotted. The mean RMSE, HTR measure, and table are based on all HTs, which are also displayed in Supplemental Figure S3.

For the default scenario (scenario 001) with allelic effects only and a continuous outcome, all methods perform well for low ambiguity with the HTR measure (Figure 3). In most other sub-scenarios, the no-outcome model outperforms the three outcome models. Exceptions are the allelic and forward-backward models for medium LD, high ambiguities, and the highest effects. For high LD, and the same ambiguities and effects all methods performed similar again. Population HTFs are consistently well estimated by the no-outcome model and almost always outperform the three outcome models, with lower RMSE and KLD ratios substantially lower than 1 (Figure 3). With specific sub-scenarios, the outcome models can perform adequately, but the no-outcome model is never substantially worse.

Overall, estimates obtained by the penalized model perform relatively worst, reflecting the bias-variance trade-off of this model. The allelic and forward-backward model perform best under several sub-scenarios, however, the no-outcome model performs better in other sub-scenarios and is most consistent across all sub-scenarios.

The HTF estimates of the scenario with haplotype effects (scenario 002) shows similar results, except for a drop of the performance of the allelic model (Supplemental Figure S4(A)). In all other cases, the no-outcome performs either better or comparable to the outcome models.

#### 4.2.2. Root mean square errors

**Figure 4.**
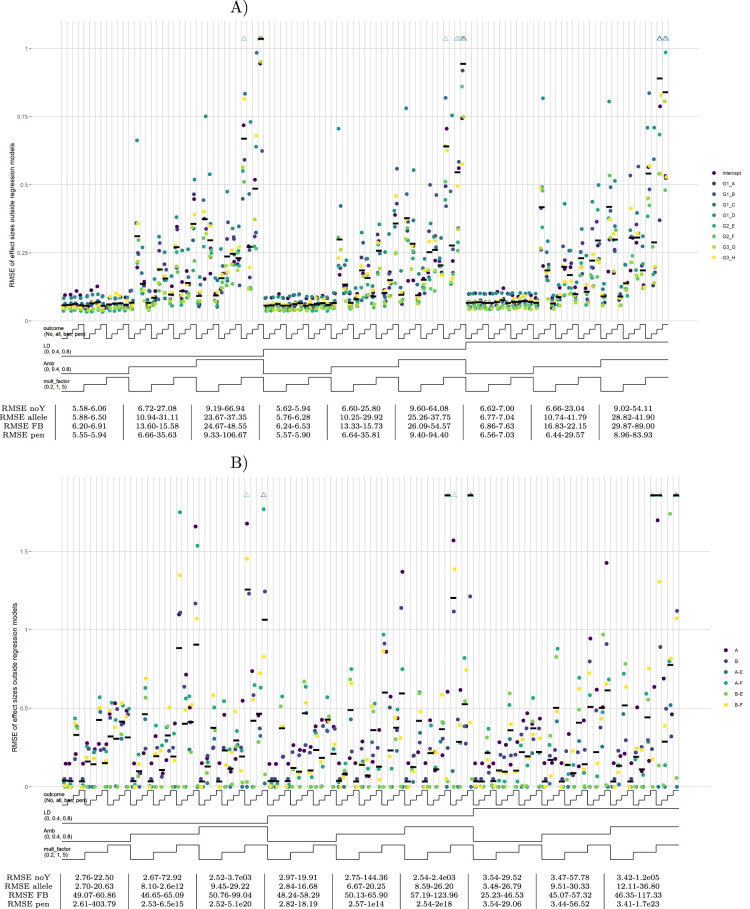
Substantive model regression coefficients, scenario 001. Note: Nested-loop plots of RMSE values for estimated regression coefficients for all sub-scenarios of default scenario 001, containing HTF and effect size set 1. Two substantive models are run with different sets of predictors: (A) only alleles as predictor and (B) all candidate list predictors as predictor. Explanation about the nested-loop plots is given in the legend of Figure 1. Here, the colored dots are either alleles (single letters) or haplotypes (combination of alleles separated by a “ − ”). The gene origin of each allele is conform Table 1. The horizontal bold black lines represent the RMSE of the effect sizes, with the values displayed in the table below the graph. These RMSE values give the range (min–max) for the three sub-scenarios above. Mean RMSE values have been multiplied by 100 for readability. In this plot, only the six HTs with the highest HTFs are plotted. The mean RMSE, HTR measure, and table are based on all HTs, which are also displayed in Supplemental Figure S5.

For the substantive models with only allelic predictors, the default scenario with high effect sizes shows superior performance of the forward-backward model, and especially of the allelic models, compared to the no-outcome model in terms of RMSEs of regression coefficients (Figure 4(a)). Including all candidate list predictors in the substantive model, the allelic outcome model still performs well, while the forward-backward model shows inconsistent behavior (Figure 4(b)). For both substantive models, the penalized model performs well for a low amount of ambiguities but starts to deteriorate for more ambiguities. In the scenario, where also haplotype effects are simulated, the beneficial effect of the allelic outcome model is attenuated (Supplemental Figure S6(A) and (B)). The allelic outcome model now only outperforms the no-outcome model with candidate list predictors in the substantive model, while for the other sub-scenarios it always performs worse. The RMSE values for the substantive model regression coefficients for the three outcome models in the other scenarios are equal to, or worse than the no-outcome model (Supplemental Figure S6(C) and (D)). Especially with high effect sizes, RMSEs can be very large for the outcome models (Supplemental Figure S6(E) to (H)). More results of the partially factorial design simulation are given in SupplementalAppendix D.

### 4.3. Data analysis

#### 4.3.1. Set-up

The KIR data contains genotype data of seven genes (*KIR2DL3*, *KIR2DL1*, *KIR2DS1*, *KIR2DL2*, *KIR2DS4*, *KIR3DL1*, and *KIR3DS1*), which is a subset of the known *KIR* genes. The current analysis is based on the subset of 1030 donor-patients pairs registered with EBMT for whom survival outcome data were available and who had no “POS” denotation for any of the seven genes.^14,27^ For the data analysis, different grouping threshold values were used ( $1\times 10^{-i}$ for i∈{4,5,6,7,8,10,12,15} ), iterating over all seven genes as starting genes in the algorithm. After each analysis, the final gene sequence was converted into one common gene order to facilitate comparisons between analyses with different starting genes.

In each analysis, the no-outcome as well as the three outcome models (alleles only, forward-backward, and penalized) were included. Survival outcome was converted into a binary outcome by computing the survival indicator at 6 months, patients censored before 6 months were considered to be alive at 6 months. In total, 229 (22.2%) patients had experienced the event. For both, the inside EM algorithm regression models as for the substantive models a logistic outcome model was used.

Regression coefficients obtained via the outcome and substantive model were used to compare outcome models with the no-outcome model. Additionally, HTFs were compared to a superset of the data (containing an additional 2517 genotype donors for which no patient outcome is available)^11^ and to estimates obtained from another study.^15^

#### 4.3.2. Results

**Figure 5.**
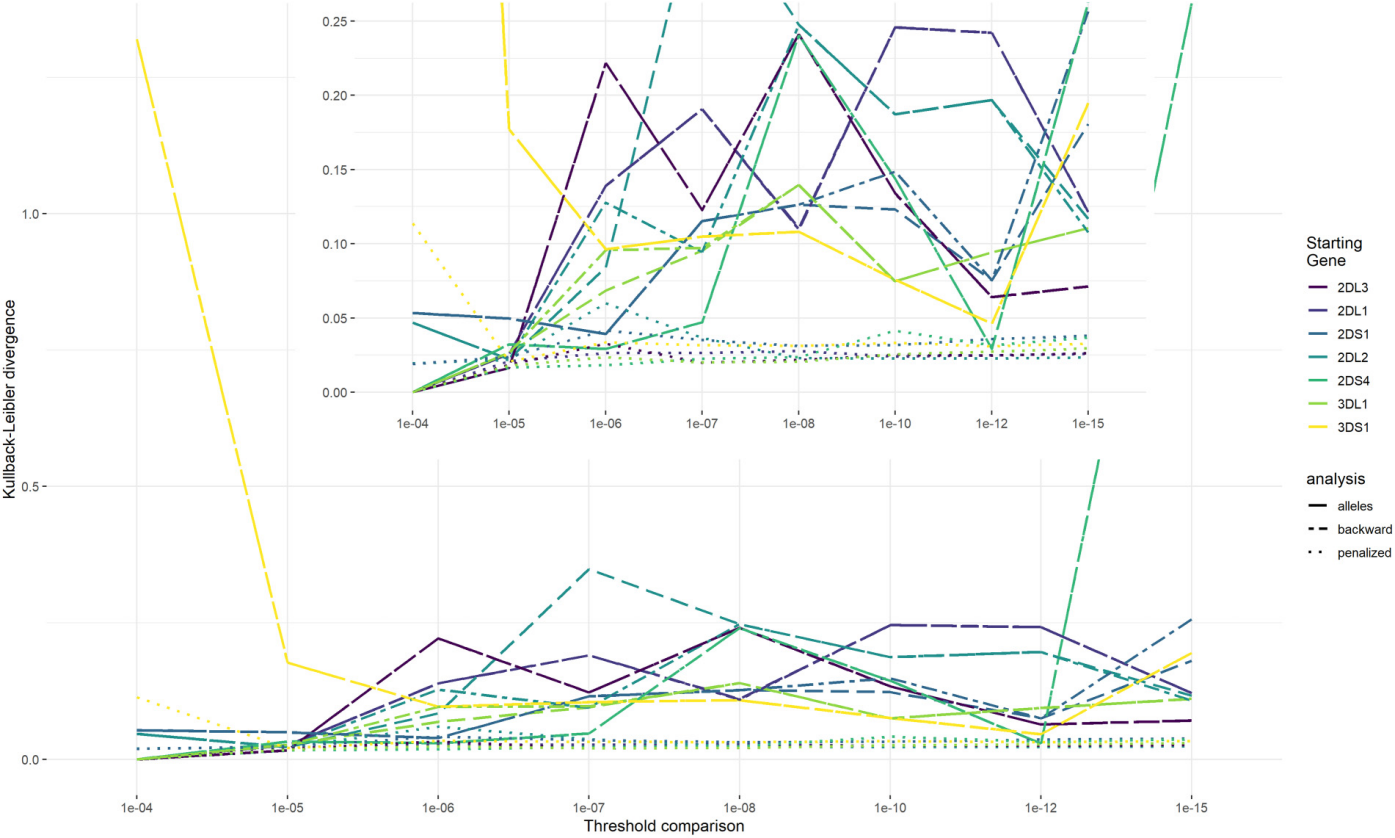
HTF dissimilarity. Note: The dissimilarity between the HTFs of the no-outcome EM algorithm and the HTFs estimated with the three outcome models for different threshold values ( $1\times 10^{-i}$ for i∈{04,05,06,07,08,10,12,15} ) estimated with the KLD. When HTFs of two models are identical, KLD equals 0. HTFs: haplotype frequencies; EM: expectation-maximization; KLD: Kullback-Leibler divergence.

All genes were considered as starting genes in the reconstruction. KLDs comparing results of the no-outcome EM algorithm with the three outcome EM algorithm strategies are shown in Figure 5 and are used to evaluate the collapsing threshold. The KLD measures do not show monotonic behavior with decreasing threshold values which would allow to choose a threshold below which KLD values would not change substantially. Instead, we use a collapsing threshold of $10^{-7}$ , following previous work.^11^

Table 3 shows the estimated HTFs for the different methods with this threshold over the seven starting genes. HTFs estimated by the four models are very similar. Only the SDs (calculated over the different starting genes) in the allelic model are substantially larger compared to the other methods. Interestingly, despite the almost identical effect size estimates in the regression models, HTFs of allelic and forward-backward models are not more similar to each other as compared to other models. Compared with the superset and the external study, the four methods tested here result in comparable HTFs estimates. These differences indicate that method heterogeneity is small compared to sample fluctuation. Overall no substantial inconsistencies were observed. This is also the case for the information $R^{2}$ (Supplemental Table S2), where no substantial information gain or loss is observed, independent of the starting gene. Only for alleles with very low AF, there is information gain.

**Table 3.** Haplotype frequency comparison.

Haplotypes	Solloch	Superset	No-outcome	Allelic	Forward-backward	Penalized
001-003c-NEG-NEG-010-005-NEG	0.0994	0.0864 (0.0003)	0.0844 (0.0001)	0.0886 (0.0023)	0.0894 (0.0003)	0.0895 (0.0001)
001-003c-NEG-NEG-003-001c-NEG	0.0689	0.0672 (0.0002)	0.0745 (0.0004)	0.0772 (0.0008)	0.0775 (0.0005)	0.0775 (0.0005)
002-001c-NEG-NEG-006-004-NEG	0.0822	0.0747 (0.0002)	0.0718 (0.0001)	0.0767 (0.0020)	0.0774 (0.0004)	0.0775 (0.0002)
001-003c-002c-NEG-NEG-NEG-013c	0.0293	0.0505 (0.0003)	0.0538 (0.0003)	0.0541 (0.0063)	0.0518 (0.0005)	0.0515 (0.0002)
NEG-004-002c-001-NEG-NEG-013c	0.0252	0.0474 (0.0001)	0.0498 (0.0003)	0.0466 (0.0206)	0.0544 (0.0003)	0.0546 (0.0003)
001-003c-NEG-NEG-006-004-NEG	0.0456	0.0464 (0.0002)	0.0483 (0.0003)	0.0445 (0.0007)	0.0447 (0.0004)	0.0448 (0.0003)
NEG-NEG-NEG-003-001c-002-NEG	0.0430	0.0474 (0.0001)	0.0432 (0.0005)	0.0461 (0.0010)	0.0464 (0.0004)	0.0462 (0.0005)
001-003c-NEG-NEG-001c-015c-NEG	0.0430	0.0343 (0.0001)	0.0420 (0.0001)	0.0438 (0.0011)	0.0442 (0.0002)	0.0442 (0.0002)
002-001c-NEG-NEG-001c-002-NEG	0.0329	0.0324 (0.0003)	0.0411 (0.0004)	0.0423 (0.0008)	0.0425 (0.0005)	0.0423 (0.0003)
002-001c-002c-NEG-NEG-NEG-013c	0.0117	0.0304 (0.0001)	0.0340 (0.0004)	0.0342 (0.0049)	0.0324 (0.0006)	0.0322 (0.0005)
002-001c-NEG-NEG-003-001c-NEG	0.0424	0.0336 (0.0002)	0.0330 (0.0006)	0.0290 (0.0012)	0.0292 (0.0009)	0.0292 (0.0006)
002-001c-NEG-NEG-010-005-NEG	0.0291	0.0346 (0.0001)	0.0308 (0.0005)	0.0298 (0.0010)	0.0301 (0.0010)	0.0296 (0.0007)
NEG-NEG-NEG-003-003-001c-NEG	0.0152	0.0289 (0.0003)	0.0256 (0.0003)	0.0270 (0.0008)	0.0274 (0.0007)	0.0273 (0.0003)
001-003c-NEG-NEG-003-008-NEG	0.0025	0.0285 (0.0001)	0.0229 (0.0003)	0.0241 (0.0006)	0.0243 (0.0003)	0.0241 (0.0003)
001-003c-NEG-NEG-001c-002-NEG	0.0120	0.0173 (0.0000)	0.0228 (0.0001)	0.0191 (0.0007)	0.0193 (0.0002)	0.0194 (0.0001)
NEG-004-NEG-001-006-004-NEG	0.0101	0.0168 (0.0001)	0.0203 (0.0003)	0.0213 (0.0036)	0.0201 (0.0005)	0.0198 (0.0004)
001-003c-006-NEG-NEG-NEG-013c	0.0089	0.0140 (0.0000)	0.0174 (0.0003)	0.0183 (0.0003)	0.0183 (0.0003)	0.0182 (0.0003)
NEG-NEG-002c-003-NEG-NEG-013c	0.0133	0.0186 (0.0002)	0.0143 (0.0001)	0.0148 (0.0025)	0.0137 (0.0005)	0.0142 (0.0001)
NEG-004-NEG-001-010-005-NEG	0.0044	0.0096 (0.0002)	0.0131 (0.0004)	0.0135 (0.0035)	0.0122 (0.0004)	0.0121 (0.0004)
002-001c-NEG-NEG-003-008-NEG	0.0019	0.0116 (0.0000)	0.0120 (0.0001)	0.0120 (0.0003)	0.0119 (0.0004)	0.0121 (0.0001)

Note: Average HTFs over the seven starting genes in the different EM algorithm (between brackets the standard deviations over the seven starting genes). The 20 haplotypes with the highest HTFs in the baseline EM algorithm without outcome are shown and ordered numerically. Additionally, the HTFs observed in a study, here used as a rough comparison^15^ and the superset. Analyses were run with $1\times 10^{-07}$ as threshold value and haplotype names were recoded into a common order (*KIR2DL3-KIR2DL1-KIR2DS1-KIR2DL2-KIR2DS4-KIR3DL1-KIR3DS1*). HTFs: haplotype frequencies; EM: expectation-maximization; KIR: killer-cell immunoglobulin-like receptor.

The two substantive models run after the EM algorithm do not support a beneficial effect of the incorporation of the outcome in haplotype reconstruction for this dataset either, probably because of over-parameterization of the models (Supplemental Appendix E). The substantive models with candidate list haplotypes as predictors are estimated as intercept-only models for both the no-outcome and the outcome methods, whereas for all methods the substantive model with allelic predictors results in unrealistically large effect sizes.

## 5. Discussion

In this article, we have addressed the problem of missing data analysis in the context of genetic data. While we have implemented a specific model, our findings have implications beyond the genetic situation, which is discussed after our specific findings.

Here, we extended an EM algorithm^11^ for haplotype reconstruction that can deal with a very general class of missing data, including ambiguities of measurements. Our extension allows to incorporate an outcome model in the imputation process, which is deemed necessary for unbiased estimates and which could potentially improve efficiency of regression models.^3^ In the genetic situation, it is generally difficult to specify a “true” regression model, also called the substantive model, which can be used during reconstruction and later for the final analysis. We have therefore extended the profile EM algorithm by allowing to choose an outcome model used during reconstruction independently from a substantive model for the final analysis. In genetics, marginal analyses are often of interest, even when higher-dimensional effects can be suspected.^28,29^ This suggests that for high(er)-dimensional data, two outcome models might be required: one for imputation and one for estimation of effects.

In the simulations, we found that there can be a huge benefit of incorporating the outcome in the EM algorithm. Both the allelic and forward-backward regression models outperform the no-outcome EM algorithm model with respect to the HTR measure and RMSEs of regression coefficients for multiple sub-scenarios. This pertains to the proof-of-concept simulations with high effects sizes and a few other simulation scenarios. On the other hand, the outcome-based EM algorithm can perform poorly for these measures in most other scenarios (Figure 3). Our results show that, at a minimum, regression models have to be correctly specified as the EM algorithm with outcome also performed poorly in the proof-of-concept simulations under misspecification. Another aspect is model size. Saturated, nearly saturated or penalized models performed poorly in general. We used penalized regression to offer a generic solution: the outcome model always contains all possible haplotype indicators and the user does not have to choose a model explicitly. However, the inherently more sparse allelic and forward-backward selection models strongly outperformed penalized regression under correct specification. We used elastic net regression ( α=0.5 ); more sparse options are available which were not fully explored. We believe, however, that no universally applicable setting can be suggested other than preferring sparse models in general. As a possible explanation, in our case, the analysis of EM trajectories of regression coefficients suggests that the EM algorithm is quite sensitive to starting values: once initial regression coefficient estimates are far enough off of the truth, estimates tend to not converge to true values but to a different local optimum for both regression coefficients and HTFs (data not shown).

A possible strategy is to run both the no-outcome and EM algorithm with outcome and compare estimates, and then accept the outcome-based estimates only if they do not diverge too strongly from no-outcome findings. While it was possible to make such a strategy based on the Euclidean distances work under specific scenarios, we were not able to define a meaningful acceptance cut-off in general because it depends highly on parameter settings (data not shown). We therefore do not see this as a promising strategy.

In summary, a benefit of an EM algorithm with outcome could only be demonstrated in simulations where missingness was modeled based on the real KIR dataset, but effect sizes were chosen higher than can be expected for real data. Especially in the genetic situation, effect sizes are deemed to be small, in general.^12^ In all other cases, the no-outcome EM algorithm performed comparable or better than the outcome model. Additionally, the regression model has to be correctly specified, when in practice, the genetic pathway is usually unknown.

In our data analysis, the beneficial impact of the outcome-models was limited. HTFs do not differ substantially and the information $R^{2}$ estimates do not show information gain. In this analysis, the penalized model performed most robustly against changes in the parameters (starting gene and grouping threshold) owing to the fact that it always shrank back to the intercept model ( $\beta_{1},\ldots ,\beta_{n}$ are set to 0). This intercept model is still different from the no-outcome model, as several iterations of the EM algorithm might have run until it was reached. This finding is, in principle, compatible with findings in the literature where reports of effects of *KIR* genes on survival in the context of allogeneic hematopoietic stem cell transplantation have been inconsistent,^14,17^ suggesting small or absent effects. Care in this interpretation has to be taken though, since it is doubtful how reliable the penalized outcome model is, as shown by the simulation study.

We believe that our findings allow for some general conclusions about handling missing data in similar situations. While we have not implemented a MI algorithm, results from our EM algorithm can be used to create multiple imputed dataset. Each EM algorithm iteration involves computing the diplotype-distribution of missing values for each individual from which data can be drawn (Figure 1). We see the use of an EM algorithm *versus* a MI strategy as two different ways to estimate the same substantive model and, therefore, believe that our findings apply to a wide range of models, including MI algorithms, for handling missing data under the MAR assumption. Multiple imputation including the outcome variable in the imputation data has been shown to increase efficiency when implemented by regressions,^30^ predictive mean matching,^10^ or substantive model based imputations.^5^ In these applications, however, the substantive model is known and it is possible to obtain reliable parameter estimates, that is, the substantive model can be well fitted. Our results show that these conditions are crucial for successful data imputation. Whenever model uncertainty becomes large either due to high dimensionality or poor conditioning of design matrices, the inclusion of a (working) outcome model is likely to be detrimental. To benefit from a substantive model when data are imputed it is therefore critical to be able to substantiate the choice of outcome model and to assess it’s model fit.

### 5.1. Conclusions

Regression model-based imputation seems beneficial in many cases, at least when the model is correctly specified, driven by true associations, and the model is well-conditioned (i.e. the design-matrix of a regression model is well-conditioned). However, misspecification can lead to substantial bias and decrease in efficiency. Outcome-based models are therefore not automatically “better” than no-outcome models. Especially in situations where it is challenging to correctly specify a substantive model—for example, due to high-dimensional predictors or interactions—, incorporating the outcome in the imputation procedure could do more harm than good. Therefore, outcome and no-outcome models have always to be performed together to check plausibility. In the high-dimensional setting, sparse models are required, but difficult to construct, especially if the process to be modeled is not well understood. Benefits of outcome-based imputation in such settings are doubtful.

## Supplemental Material

sj-pdf-1-smm-10.1177_09622802241304112 - Supplemental material for High-dimensional, outcome-dependent missing data problems: Models for the human KIR loci Supplemental material, sj-pdf-1-smm-10.1177_09622802241304112 for High-dimensional, outcome-dependent missing data problems: Models for the human KIR loci by Lars Leonardus Joannes van der Burg, Hein Putter, Henning Baldauf, Jürgen Sauter, Johannes Schetelig, Liesbeth C deWreede and Stefan Böhringer in Statistical Methods in Medical Research
