# Supplementary material for: High-dimensional, outcome-dependent missing data problems: Models for the human KIR loci
Source: Stat Methods Med Res. 2025 Jan 31;34(3):440–56. doi: 10.1177/09622802241304112 (PMC11951372; doi:10.1177/09622802241304112)
Supplement: sj-pdf-1-smm-10.1177_09622802241304112 - Supplemental material for High-dimensional, outcome-dependent missing data problems: Models for the human KIR loci [file sj-pdf-1-smm-10.1177_09622802241304112.pdf]

Appendix A: Outcome likelihood rescaling

In the E-step of the EM-algorithm $P(Y_i|H_i)$ are rescaled. As the algorithm optimizes all HTFs in every step, in principle, collapsed haplotypes (i.e., haplotypes composed of the previous collapsed haplotype set and alleles at the added locus) can increase in frequency in subsequent iterations. As collapsing is only performed for haplotypes that are unlikely to exist in the data, such behavior would be undesirable (van der Burg et al., 2023). To limit the influence of high probabilities $P(Y_i|H_i)$ for collapsed haplotypes compared to non-collapsed haplotypes, a scaling factor is chosen so that the sum of likelihood terms across all haplotypes in the collapsed group is c_{thresh}, and $1 - c_{thresh}$ for the group of non-collapsed haplotypes. We choose $c_{thresh} = 0.5$, which boils down to all collapsed haplotypes are scaled to compose half of $P(Y_i|H_i)$ and all non-collapsed haplotypes comprise the other half. For example, consider an individual with 4 haplotypes, two collapsed with frequencies before rescaling of 0.3 and 0.4 and two non-collapsed with frequencies 0.1 and 0.2. With $c_{thresh} = 0.5$ the probabilities of each group must sum up to 0.5, so the collapsed HTFs are rescaled to 0.21 and 0.29, where the non-collapsed HTFs are rescaled to 0.167 and 0.333. In practice this choice leads to probabilities in Equation (6) to be close to those when ignoring collapsed haplotypes. As a special case, if $\sum P(Y_i|H_i) < 1e^{-10}$, i.e., all effect sizes are approximately zero, rescaling is omitted.

Appendix B: Reparametrization

The haplotype is partitioned into $K-1$ loci, with the $K-1$ loci indexed by i, and the single, remaining locus, with the alleles indexed by j. HTFs at all loci are therefore given by the vector $(\pi_{(i,j)})_{(i,j)}$. We can now derive marginal HTFs and LD parameters as follows:

$$\pi_i := \sum_j \pi_{(i,j)}, \pi_j := \sum_i \pi_{(i,j)}, \delta_{i,j} := \pi_{(i,j)} - \pi_i \pi_j$$

The mapping $(\pi_{(i,j)})_{(i,j)} \mapsto ((\pi_i)_i, (\pi_j)_j), (\delta_{i,j})_{(i,j)})$ is bijective and thereby defines a reparametrization.

Appendix C: Partial factorial design

Table S1 displays the 16 possible scenarios with the combination of the two sets of HTFs; the two sets of regression coefficients; the two sets of haplotype regression coefficients and the two types of outcomes. Eight of these scenarios are evaluated in the partial factorial design simulation study.

Scenario	Name	HTF set	RC set	haplotype RC set	Outcome	Evaluated
1	001	-	-	-	-	yes
2	011	+	-	-	-	yes
3	002	-	+	-	-	yes
4	012	+	+		-	yes
5	003	-	-	+	-	yes
6	013	+	-	+	-	yes
7	004	-	+	+	-	no
8	014	+	+	+	-	no
9	001*	-	-	-	+	yes
10	011*	+	-	-	+	no
11	002*	-	+	-	+	yes
12	012*	+	+	-	+	no
13	003*	-	-	+	+	no
14	013*	+	-	+	+	no
15	004*	-	+	+	+	no
16	014*	+	+	+	+	no

Table S1: partial factorial design. All 16 different scenarios with scenario code and indication of the eight scenarios that are actually evaluated. The minus indicates that the default value is used for that scenario, where a plus indicates the use of the other set.

Appendix D: Additional results

Proof-of-concept results

For scenario A, the benefit of the allelic and forward-backward regression models over the no-outcome EM-algorithm in terms of the HTR measure is limited without large effect sizes (Figure S7). Where with substantial ambiguities and LD, and high effects both quickly outperform the other two methods, the high effect sizes prove to be essential. With lower effect sizes, the no-outcome model performs comparable or better. This also holds for the population HTFs. Only with the high effect sizes the allelic and forward-backward regression models can perform better than the no-outcome model. For scenario B - where haplotype effects are simulated - the population HTFs and HTR scores are quite comparable to those observed for scenario A (Figure S8). With high effect sizes the no-outcome model can be beaten. The main difference is that the RMSEs, HTR values and KLD-ratios obtained with the allelic model are poorer than in scenario A, while the estimates of the forward-backward and penalized models remain similar.

In each EM-algorithm with an outcome model, this outcome model is run every iteration with the current HTFs as predictor. Figure S9A shows the estimated regression coefficients from the outcome models from the last EM-algorithm iteration for the proof-of-concept simulation without allelic effects. With low effect sizes the regression coefficients of the allelic and penalized model are substantially better than those of the forward-backward model, however, with higher effect sizes the estimates from the penalized model deteriorate much faster, resulting in RMSEs that are worse. The pattern is different with haplotype effects simulated (scenario B; Figure S9B). The three methods with outcome models have comparable RMSEs with low effect sizes, while with high effect sizes the RMSEs of the forward-backward model are much better than those of the other two. Only under the combination of high LD and high ambiguities the forward-backward model is outperformed by the other two. In general, the estimates from scenario B are substantially higher than those observed in scenario A, indicating that the added haplotype effects are difficult to deal with.

The impact of the outcome is clearly visible in the coefficients from the substantive models. With only allelic predictors and no simulated ambiguities, the different models have similar errors (Figure S10A). However, with intermediate or high ambiguity and sufficient effect sizes the RMSEs of the no-outcome and penalized models are much higher than those of the allelic and forward-backward regression models. Only when both high LD and high ambiguities are added, all models behave comparably poor. When we use all candidate list predictors as predictors for the substantive model, similar observations are made (Figure S10B). The beneficial effect of the allelic and forward-backward regression models are now also observed for the high LD and high ambiguities scenario. For scenario B comparable observations are made, with the allelic and forward-backward model having the lowest RMSE values with sufficient ambiguities and effect sizes (Figure S10C & S10D). However, both models have for certain sub-scenarios regression coefficients similar to those of the no-outcome model, and do not perform consequently better.

Partial factorial design simulation study results

For the HTFs of the other sub-scenarios (except scenario 002) the RMSE values are higher and the KLD ratios lower compared to the default scenario. In terms of the HTR measure, the no-outcome model performs best in most cases. Simulations with the uniform haplotype set (scenario 011; Figure S4B) shows that for the sub-scenarios with low or medium LD, high ambiguities and high effect sizes, the allelic, and especially the forward-backward models have lower RMSEs; KLD ratios exceeding one and considerably better HTR measures. For other sub-scenarios, the no-outcome model performs best. Related scenarios (uniform HTF set and haplotype effects: scenario 012) or the use of strong CNV effects (scenario 003 and 013) shows similar patterns (Figure S4C-E). Results on HTFs with the binomial outcome (scenarios 001* and 002*) are very comparable (Figure S4F & S4G), where in most cases, the outcome models perform worse than the no-outcome model in terms of RMSE, KLD ratios and HTR.

The RMSEs of the working outcome model regression coefficients for scenario 001 showed similar values for the allelic and penalized models, whereas RMSEs for the forward-backward model are higher (Figure S11A). Upon increasing the effect sizes, the RMSEs of forward-backward and penalized models increase, while in contrast, the allelic model improves. Adding haplotype effects (scenario 002), the regression coefficients of the forward-backward and penalized model remain similar (Figure S11B), whereas the allelic model performs worse, especially with higher effect sizes. Choosing frequencies to be similar (scenario 011) or assigning effects to CNV alleles (scenario 003) gives similar results (Figure S11C & S11D). Scenario 013 (uniform frequencies with CNV alleles) shows similar results, except for increased RMSEs of the allelic regression model when LD is high (Figure S11E). This effect is also observed in scenario 012 which for the other sub-scenarios resembles scenario 002 (Figure S11F).

For the binomial outcome (Figures S11G & S11H), the regression coefficients from the working outcome model show a similar pattern: allelic and forward-backward model perform equally (because all haplotype predictors are discarded this is by design, see Section 2.4.2) while the penalized model shrinks most outcome model regression coefficients to zero, leading to lower overall RMSEs.

Appendix E: supplementary material

All estimated values for the real data analysis are saved in the following five files:

- Regression coefficients for the predictors of the working outcome models for the allelic, forward-backward and penalized model.
- Effect sizes for each haplotype calculated from the regression coefficients of the working outcome models, for the three outcome models.
- HTFs estimated by the EM-algorithm, for the three outcome models and the no-outcome model.
- Regression coefficients of the substantive model with alleles as predictors, for the three outcome models and the no-outcome model.
- Regression coefficients of the substantive model with the candidate list haplotypes as predictors, for the three outcome models and the no-outcome model.

When in one of these files an item is empty for a variable, this variable was not included in the analysis with that threshold and model.

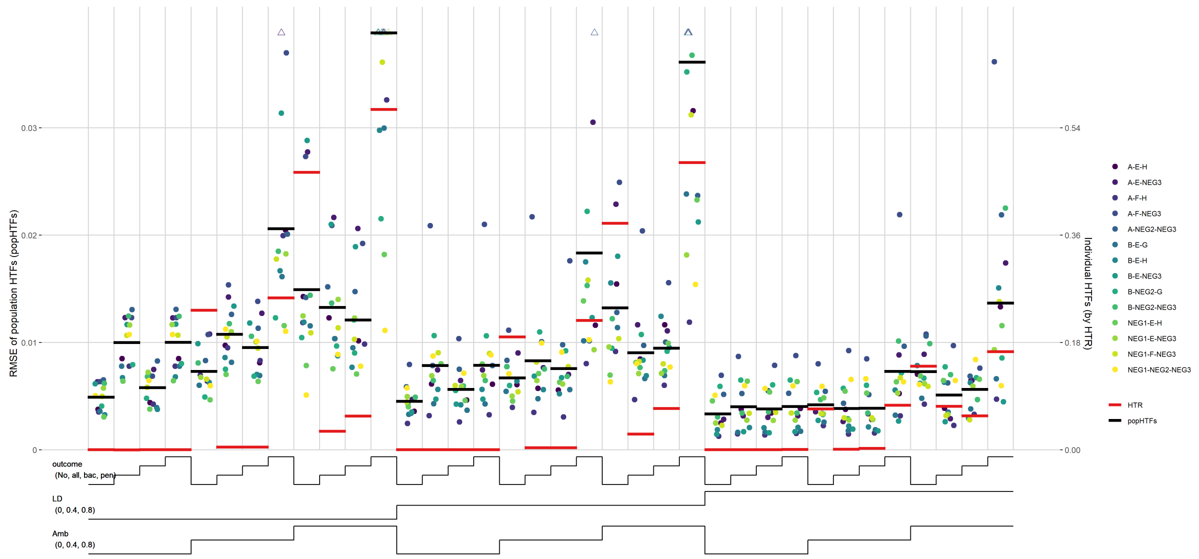

RMSE noY	0.49	0.73	1.49	0.45	0.67	1.32	0.33	0.42	0.73
RMSE allele	1.00	1.08	1.33	0.79	0.83	0.91	0.40	0.38	0.51
RMSE FB	0.58	0.95	1.21	0.56	0.76	0.95	0.38	0.39	0.56
RMSE pen	1.00	2.06	4.40	0.79	1.83	3.61	0.40	0.73	1.37
HTR noY	0.00	0.23	0.47	0.00	0.19	0.38	0.00	0.07	0.14
HTR allele	0.00	0.00	0.03	0.00	0.00	0.03	0.00	0.00	0.07
HTR FB	0.00	0.00	0.06	0.00	0.00	0.07	0.00	0.00	0.06
HTR pen	0.00	0.25	0.57	0.00	0.22	0.48	0.00	0.07	0.16
KLD allele	0.32	1.47	4.61	0.02	0.21	1.23	0.05	0.64	1.07
KLD FB	0.68	1.79	0.82	0.37	0.36	1.42	0.12	1.49	0.83
KLD pen	0.31	0.29	0.10	0.02	0.03	0.07	0.05	0.08	0.09

Figure S1: HTF estimation, scenario A. Nested-loop plot of RMSEs for HTFs and haplotype reconstruction (HTR) measure for all sub-scenarios of the proof-of-concept study without haplotype effects. Explanation about the Nested-loop plots is given in the legend of Figure 2. The horizontal bold black lines represent the RMSE of the popHTFs (with values on the left y-axis), while the bold red lines represent the HTR measure of the individual HTFs (with values on the right y-axis). The table below the graph shows mean RMSE values of these popHTFs and the HTR measures, as well as the Kullback-Leibler divergence (KLD) ratios between the no-outcome model and the three outcome models. All values in the table give ranges (min-max) of values for the three sub-scenarios above. A ratio > 1 indicates higher similarity of the HTFs of the outcome models with the truth than the no-outcome model. Mean RMSE values in the table have been multiplied by 100 for readability.

A)

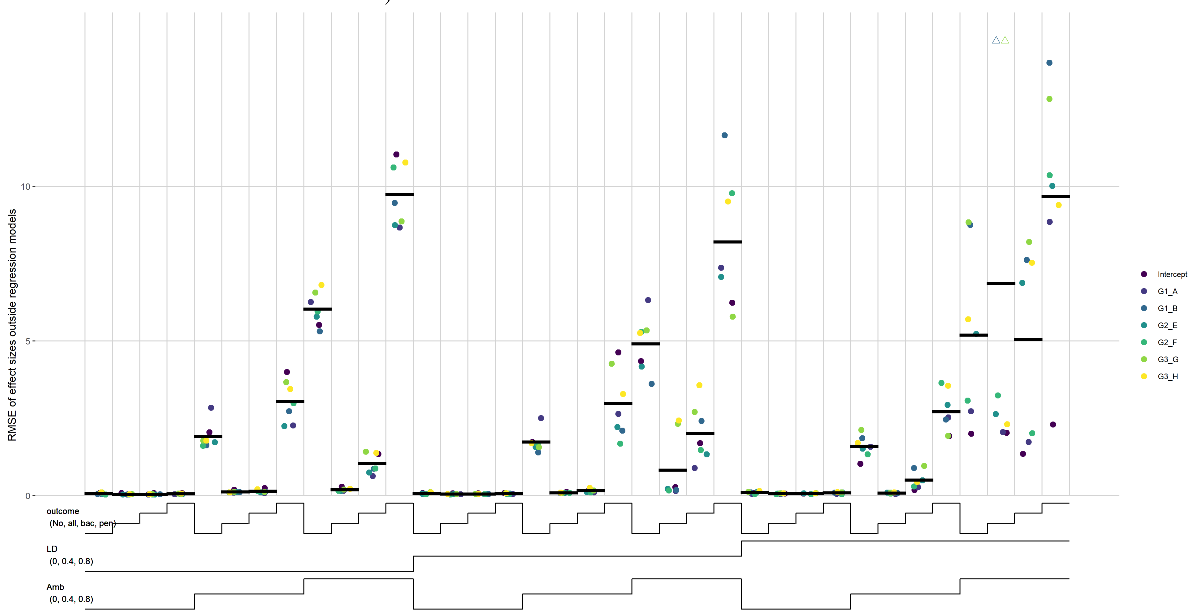

RMSE noY	6.88	191.47	602.91	7.31	173.36	490.63	9.76	159.21	518.76
RMSE allele	4.57	11.95	18.68	4.99	9.09	81.94	6.24	8.26	685.31
RMSE FB	4.57	14.55	103.74	5.30	15.67	201.14	6.70	50.40	504.73
RMSE pen	5.64	304.86	973.50	6.34	297.43	819.92	8.53	270.86	967.68

B)

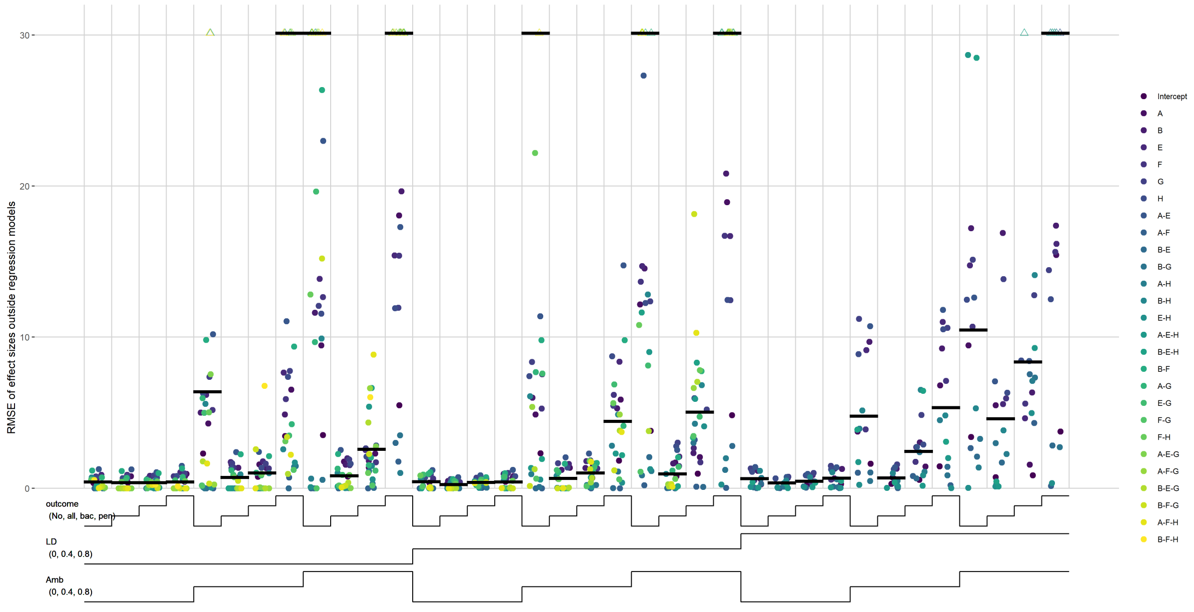

RMSE noY	41.21	639.21	4.8e03	43.98	6.9e04	8.7e03	63.55	477.33	1e03
RMSE allele	36.34	71.41	81.75	24.60	65.59	94.90	35.00	67.75	460.02
RMSE FB	37.46	101.91	257.96	39.18	101.26	503.69	46.99	243.19	835.96
RMSE pen	41.13	1.6e16	9.3e18	41.10	443.23	7.9e20	66.29	534.06	3.3e18

Figure S2: Substantive model regression coefficients. Nested-loop plots of RMSE values for estimated regression coefficients for the sub-scenarios of the proof-of-concept study A) scenario A (without haplotype effects) with only alleles as predictor, B) scenario A with all candidate list haplotypes as predictors, both only with high simulated effect sizes. Explanation about the Nested-loop plots is given in the legend of Figure 2. Here, the coloured dots are either alleles (single letters) or haplotypes (combination of alleles separated by a '-'). The gene origin of each allele is conform Table 2. The horizontal bold black lines represent the RMSE of the effect sizes, with its values displayed in the table below the graph. Mean RMSE values have been multiplied by 100 for readability.

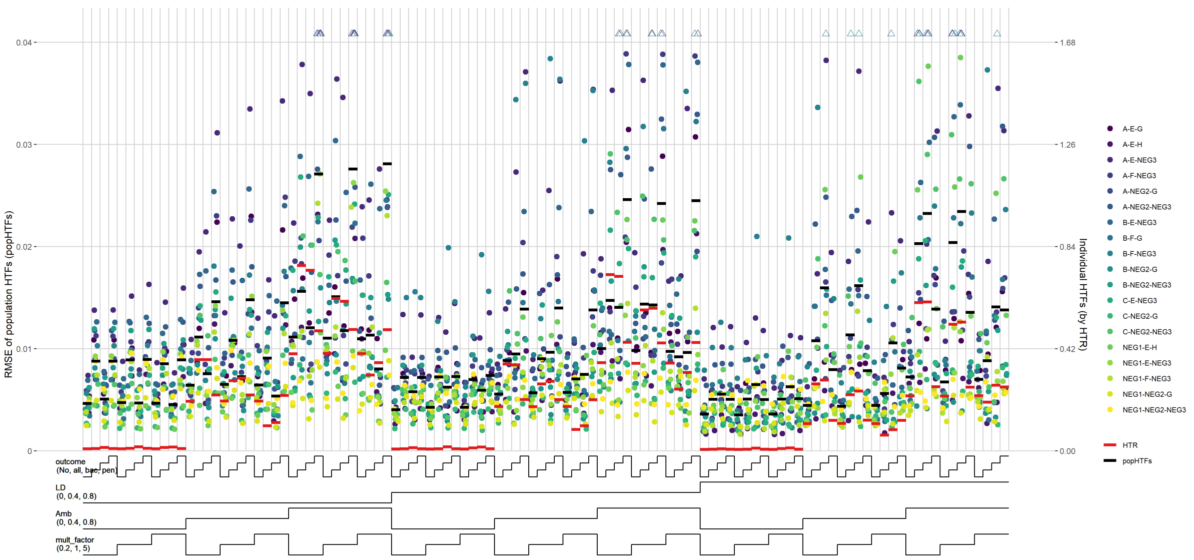

RMSE noY	0.46-0.47	0.64-0.65	1.10-1.11	0.40-0.43	0.56-0.57	0.97-1.00	0.35-0.36	0.44-0.44	0.68-0.72
RMSE allele	0.85-0.89	0.91-1.11	1.13-1.56	0.70-0.72	0.70-0.90	0.92-1.47	0.51-0.51	0.56-1.13	0.88-2.04
RMSE FB	0.45-0.51	0.54-0.76	0.80-1.21	0.58-0.62	0.75-0.97	0.96-1.43	0.56-0.65	1.06-1.62	1.41-2.34
RMSE pen	0.88-0.90	1.45-1.48	2.71-2.81	0.72-0.74	1.38-1.40	2.42-2.46	0.50-0.51	0.79-0.81	1.36-1.39
HTR noY	0.01-0.01	0.20-0.20	0.40-0.40	0.01-0.01	0.18-0.18	0.36-0.36	0.01-0.01	0.11-0.11	0.23-0.23
HTR allele	0.01-0.01	0.10-0.38	0.31-0.76	0.01-0.01	0.09-0.36	0.25-0.72	0.01-0.01	0.06-0.28	0.20-0.61
HTR FB	0.01-0.02	0.12-0.38	0.36-0.74	0.01-0.02	0.10-0.35	0.32-0.72	0.01-0.01	0.09-0.29	0.27-0.61
HTR pen	0.01-0.01	0.23-0.23	0.49-0.50	0.01-0.01	0.21-0.21	0.44-0.45	0.01-0.01	0.13-0.13	0.26-0.26
KLD allele	0.18-0.67	0.08-0.33	0.06-0.14	0.03-0.05	0.07-0.31	0.09-0.32	0.03-0.06	0.03-0.16	0.03-0.50
KLD FB	0.25-0.52	0.08-0.28	0.07-0.15	0.02-0.06	0.04-0.11	0.07-0.17	0.01-0.03	0.01-0.02	0.02-0.08
KLD pen	0.51-0.82	0.15-0.18	0.13-0.36	0.04-0.05	0.03-0.04	0.04-0.16	0.06-0.07	0.08-0.08	0.10-0.17

Figure S3: HTF estimation, scenario 001. Nested-loop plot of RMSEs for HTFs and HTR measure for all sub-scenarios of default scenario 001, containing HTF and effect size set 1. Explanation about the Nested-loop plots is given in the legend of Figure 2. The horizontal bold black lines represent the RMSE of the popHTFs (with values on the left y-axis), while the bold red lines represent the HTR measure of the individual HTFs (with values on the right y-axis). The table below the graph shows mean RMSE values of these popHTFs and the HTR measures, as well as the KLD ratios between the no-outcome model and the three outcome models. All values in the table are the range (min-max) of values for the three sub-scenarios above. A ratio > 1 indicates higher similarity of the HTFs of the outcome models with the truth than the no-outcome model. Mean RMSE values in the table have been multiplied by 100 for readability.

A)

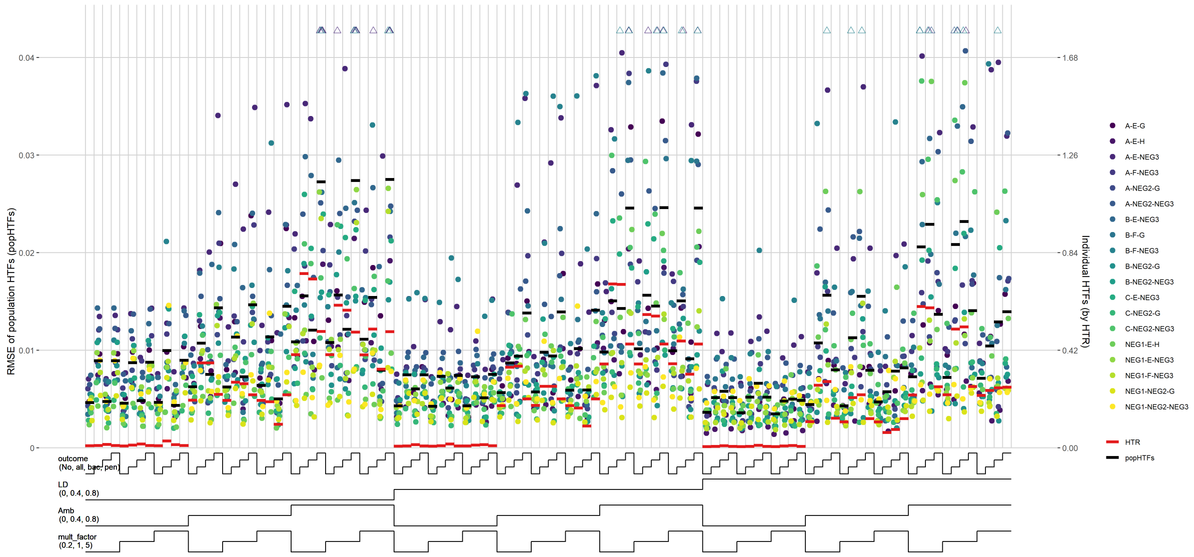

RMSE noY	0.46-0.47	0.62-0.64	1.08-1.11	0.43-0.43	0.57-0.59	0.98-1.02	0.35-0.36	0.43-0.47	0.71-0.73
RMSE allele	0.87-1.00	1.07-1.14	1.54-1.57	0.73-0.75	0.87-1.02	1.51-1.56	0.48-0.52	0.58-1.13	1.04-2.08
RMSE FB	0.43-0.48	0.50-0.75	0.80-1.21	0.51-0.61	0.59-0.94	0.91-1.45	0.50-0.66	0.79-1.56	1.29-2.32
RMSE pen	0.88-0.90	1.44-1.47	2.73-2.75	0.74-0.75	1.38-1.41	2.46-2.46	0.49-0.52	0.79-0.82	1.37-1.41
HTR noY	0.01-0.01	0.20-0.20	0.40-0.40	0.01-0.01	0.18-0.18	0.36-0.36	0.01-0.01	0.11-0.11	0.23-0.23
HTR allele	0.01-0.03	0.20-0.37	0.51-0.75	0.01-0.01	0.17-0.35	0.46-0.71	0.01-0.01	0.07-0.27	0.25-0.61
HTR FB	0.01-0.02	0.10-0.37	0.34-0.73	0.01-0.02	0.09-0.35	0.32-0.70	0.01-0.01	0.08-0.29	0.26-0.60
HTR pen	0.01-0.01	0.23-0.23	0.50-0.50	0.01-0.01	0.21-0.21	0.45-0.45	0.01-0.01	0.13-0.13	0.26-0.26
KLD allele	0.03-0.61	0.04-0.10	0.04-0.07	0.04-0.07	0.07-0.11	0.08-0.09	0.09-0.12	0.03-0.22	0.04-0.05
KLD FB	0.14-0.33	0.09-0.23	0.06-0.16	0.04-0.06	0.05-0.22	0.06-0.18	0.02-0.04	0.01-0.04	0.03-0.06
KLD pen	0.36-0.54	0.12-0.16	0.12-0.15	0.04-0.07	0.04-0.05	0.04-0.16	0.09-0.11	0.05-0.08	0.11-0.20

B)

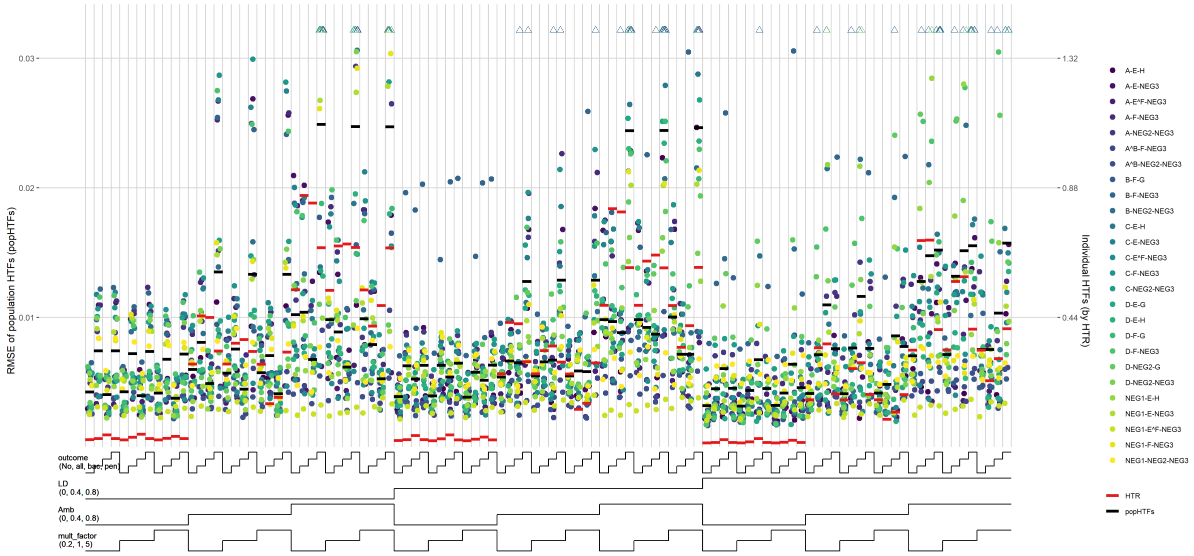

RMSE noY	0.42-0.43	0.57-0.60	0.98-1.02	0.38-0.39	0.54-0.55	0.98-1.00	0.32-0.32	0.41-0.41	0.70-0.75
RMSE allele	0.68-0.74	0.71-0.81	0.79-1.04	0.58-0.63	0.59-0.66	0.72-0.97	0.42-0.45	0.48-0.76	0.75-1.32
RMSE FB	0.38-0.40	0.41-0.49	0.53-0.67	0.51-0.54	0.58-0.67	0.71-0.88	0.61-0.67	0.86-1.16	1.03-1.51
RMSE pen	0.72-0.74	1.33-1.35	2.47-2.49	0.63-0.65	1.28-1.29	2.44-2.46	0.45-0.46	0.76-0.78	1.52-1.57
HTR noY	0.02-0.02	0.28-0.28	0.53-0.53	0.02-0.02	0.25-0.25	0.48-0.48	0.01-0.01	0.16-0.16	0.33-0.33
HTR allele	0.03-0.03	0.15-0.45	0.41-0.85	0.02-0.03	0.13-0.42	0.34-0.81	0.02-0.02	0.09-0.34	0.23-0.70
HTR FB	0.04-0.04	0.17-0.44	0.48-0.83	0.03-0.04	0.15-0.42	0.41-0.80	0.02-0.03	0.12-0.35	0.30-0.70
HTR pen	0.03-0.03	0.32-0.33	0.68-0.68	0.02-0.02	0.29-0.29	0.61-0.61	0.02-0.02	0.18-0.18	0.40-0.40
KLD allele	0.11-0.25	0.38-0.71	0.33-1.01	0.06-0.08	0.31-0.53	0.20-1.06	0.09-0.12	0.02-0.12	0.04-0.33
KLD FB	0.14-0.34	0.17-0.41	0.59-1.89	0.04-0.06	0.06-0.08	0.07-0.20	0.01-0.02	0.01-0.02	0.02-0.07
KLD pen	0.14-0.19	0.26-0.68	0.44-1.12	0.06-0.08	0.03-0.04	0.06-0.07	0.08-0.11	0.08-0.09	0.17-0.74

C)

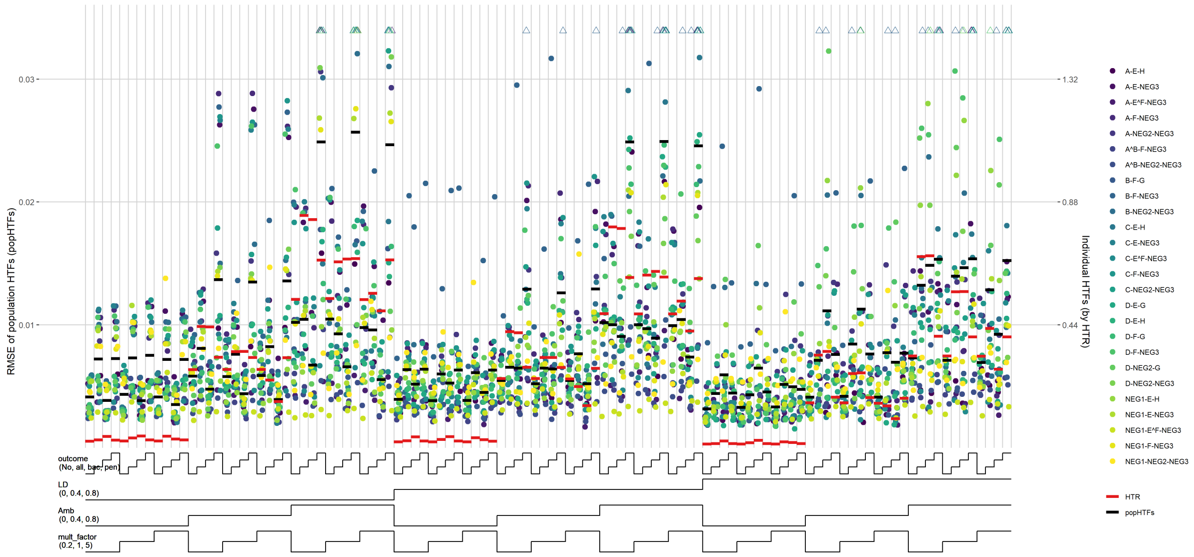

RMSE noY	0.41-0.44	0.58-0.59	0.98-1.05	0.38-0.39	0.52-0.55	0.99-1.02	0.32-0.33	0.41-0.42	0.69-0.73
RMSE allele	0.72-0.73	0.76-0.82	0.93-1.05	0.60-0.64	0.65-0.76	0.97-1.04	0.43-0.52	0.71-0.84	1.29-1.40
RMSE FB	0.35-0.39	0.39-0.48	0.56-0.67	0.45-0.53	0.52-0.65	0.74-0.91	0.50-0.65	0.70-1.13	0.92-1.48
RMSE pen	0.72-0.75	1.35-1.37	2.47-2.57	0.63-0.64	1.26-1.29	2.46-2.49	0.45-0.48	0.76-0.77	1.52-1.54
HTR noY	0.02-0.02	0.28-0.28	0.53-0.53	0.02-0.02	0.25-0.25	0.48-0.48	0.01-0.01	0.16-0.16	0.33-0.33
HTR allele	0.03-0.04	0.24-0.43	0.55-0.83	0.02-0.04	0.22-0.42	0.53-0.79	0.02-0.02	0.15-0.33	0.43-0.68
HTR FB	0.03-0.04	0.17-0.43	0.49-0.82	0.03-0.04	0.15-0.41	0.42-0.79	0.02-0.03	0.10-0.34	0.28-0.69
HTR pen	0.03-0.03	0.32-0.32	0.67-0.68	0.02-0.02	0.28-0.29	0.61-0.61	0.02-0.02	0.18-0.18	0.40-0.40
KLD allele	0.03-0.30	0.03-0.73	0.10-0.42	0.05-0.10	0.10-0.65	0.09-0.23	0.05-0.10	0.02-0.04	0.02-0.06
KLD FB	0.20-0.29	0.22-0.32	0.47-1.56	0.04-0.07	0.06-0.16	0.05-0.17	0.01-0.03	0.01-0.03	0.02-0.06
KLD pen	0.13-0.24	0.41-0.59	0.32-0.91	0.07-0.09	0.03-0.04	0.03-0.06	0.08-0.09	0.05-0.08	0.51-0.54

D)

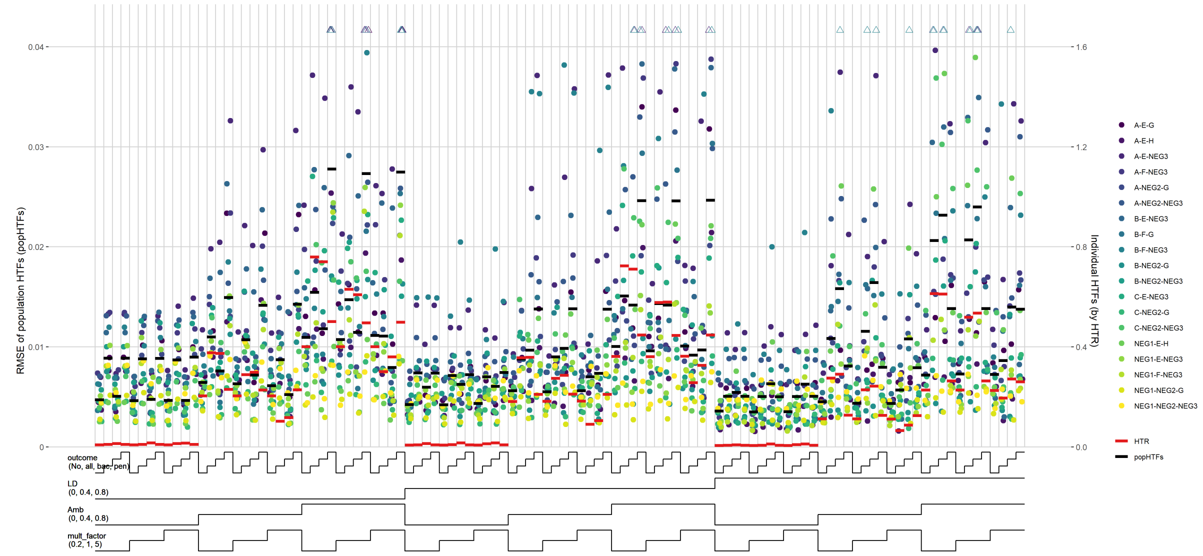

RMSE noY	0.46-0.47	0.61-0.64	1.07-1.11	0.42-0.44	0.55-0.56	0.96-1.01	0.35-0.36	0.43-0.45	0.72-0.73
RMSE allele	0.87-0.89	0.87-1.10	1.11-1.55	0.70-0.74	0.70-0.90	0.92-1.51	0.50-0.51	0.57-1.16	0.86-2.07
RMSE FB	0.47-0.51	0.52-0.76	0.79-1.18	0.60-0.63	0.74-0.98	0.97-1.42	0.54-0.63	1.08-1.64	1.40-2.40
RMSE pen	0.88-0.90	1.42-1.49	2.73-2.78	0.72-0.74	1.38-1.38	2.46-2.47	0.50-0.51	0.79-0.81	1.38-1.38
HTR noY	0.01-0.01	0.20-0.20	0.40-0.40	0.01-0.01	0.18-0.18	0.36-0.36	0.01-0.01	0.11-0.11	0.23-0.23
HTR allele	0.01-0.01	0.10-0.38	0.30-0.76	0.01-0.01	0.09-0.36	0.26-0.72	0.01-0.01	0.07-0.28	0.20-0.61
HTR FB	0.01-0.02	0.12-0.37	0.36-0.74	0.01-0.02	0.11-0.36	0.33-0.71	0.01-0.01	0.09-0.29	0.27-0.61
HTR pen	0.01-0.01	0.23-0.23	0.50-0.50	0.01-0.01	0.21-0.21	0.45-0.45	0.01-0.01	0.12-0.13	0.26-0.26
KLD allele	0.20-0.90	0.07-0.26	0.07-0.14	0.04-0.05	0.06-0.28	0.08-0.44	0.06-0.08	0.02-0.23	0.03-0.57
KLD FB	0.29-0.49	0.07-0.21	0.07-0.16	0.03-0.05	0.03-0.12	0.06-0.29	0.02-0.06	0.01-0.03	0.02-0.09
KLD pen	0.34-1.04	0.11-0.23	0.13-0.30	0.04-0.05	0.03-0.05	0.04-0.22	0.08-0.09	0.07-0.08	0.13-0.17

E)

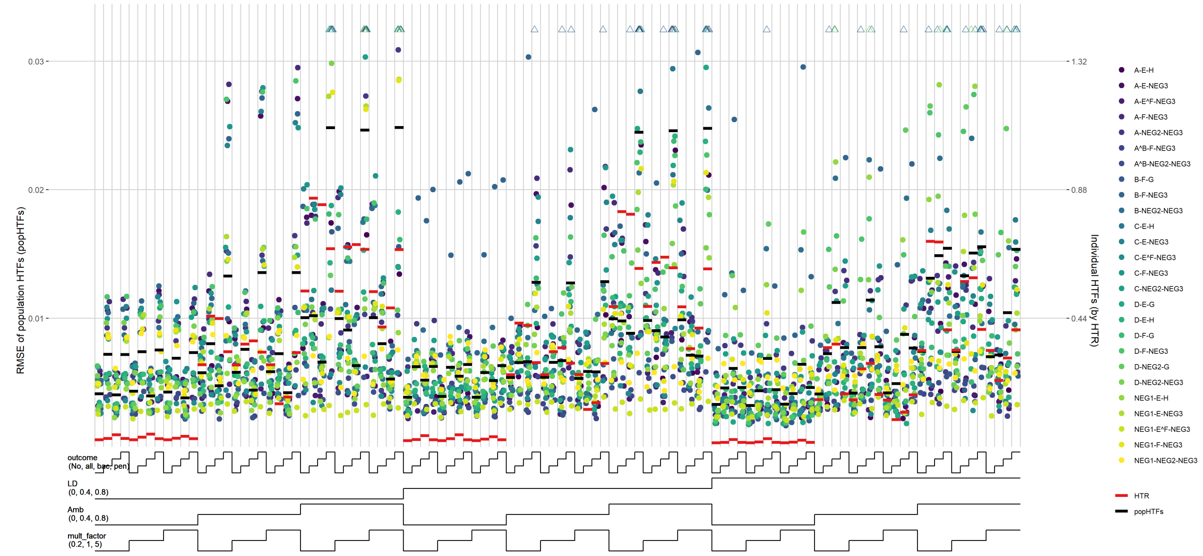

RMSE noY	0.41-0.43	0.58-0.58	1.00-1.01	0.39-0.39	0.53-0.54	0.98-1.00	0.32-0.33	0.41-0.42	0.70-0.74
RMSE allele	0.69-0.74	0.72-0.80	0.80-1.02	0.58-0.62	0.59-0.66	0.71-0.98	0.42-0.46	0.49-0.77	0.71-1.33
RMSE FB	0.38-0.41	0.42-0.48	0.53-0.66	0.51-0.55	0.58-0.67	0.71-0.88	0.61-0.69	0.88-1.14	1.04-1.51
RMSE pen	0.72-0.75	1.33-1.36	2.46-2.48	0.62-0.64	1.27-1.29	2.45-2.48	0.44-0.46	0.77-0.78	1.54-1.56
HTR noY	0.02-0.02	0.28-0.28	0.53-0.53	0.02-0.02	0.25-0.25	0.48-0.48	0.01-0.01	0.16-0.16	0.33-0.33
HTR allele	0.03-0.03	0.15-0.45	0.41-0.85	0.02-0.03	0.13-0.42	0.34-0.81	0.02-0.02	0.09-0.34	0.23-0.70
HTR FB	0.04-0.04	0.17-0.44	0.48-0.83	0.03-0.04	0.15-0.42	0.41-0.80	0.02-0.03	0.12-0.35	0.30-0.70
HTR pen	0.03-0.03	0.32-0.33	0.68-0.68	0.02-0.02	0.29-0.29	0.61-0.61	0.02-0.02	0.18-0.18	0.40-0.40
KLD allele	0.11-0.17	0.38-0.82	0.47-0.87	0.06-0.11	0.26-0.54	0.15-1.70	0.09-0.11	0.03-0.13	0.03-0.11
KLD FB	0.17-0.27	0.27-0.31	0.73-1.57	0.03-0.08	0.05-0.09	0.05-0.30	0.02-0.02	0.01-0.02	0.02-0.03
KLD pen	0.16-0.28	0.36-0.72	0.41-8.08	0.05-0.10	0.02-0.04	0.03-0.10	0.09-0.11	0.07-0.10	0.44-0.61

F)

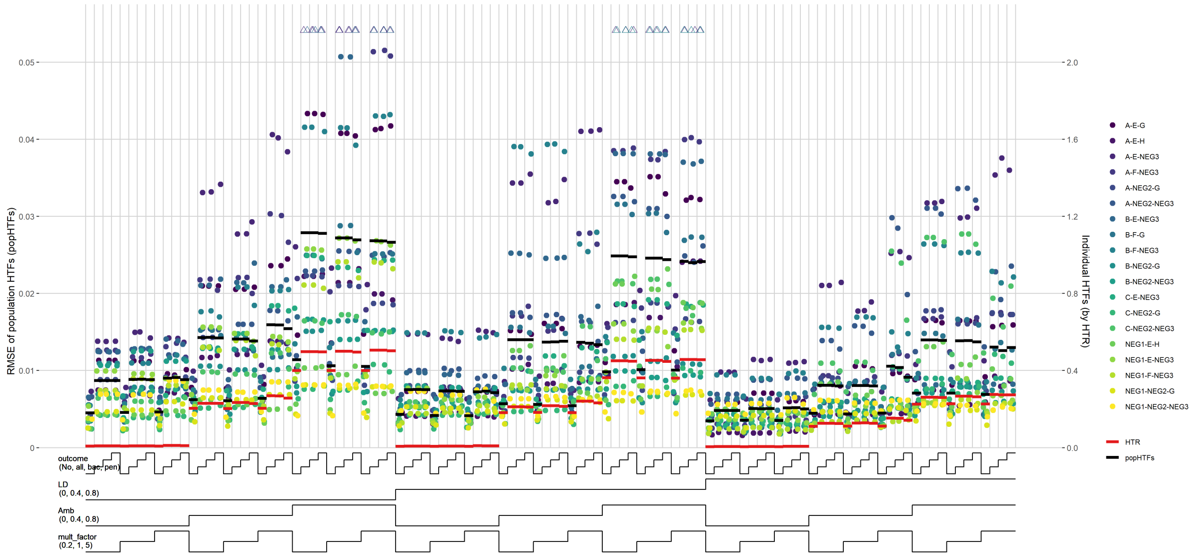

RMSE noY	0.45-0.46	0.61-0.64	1.05-1.14	0.42-0.43	0.55-0.57	0.98-1.01	0.35-0.36	0.44-0.45	0.69-0.71
RMSE allele	0.87-0.90	1.41-1.60	2.68-2.79	0.73-0.75	1.37-1.40	2.42-2.49	0.48-0.52	0.81-1.06	1.31-1.40
RMSE FB	0.87-0.91	1.41-1.59	2.68-2.79	0.73-0.76	1.36-1.40	2.40-2.49	0.48-0.52	0.81-1.04	1.26-1.40
RMSE pen	0.87-0.88	1.38-1.54	2.66-2.78	0.71-0.75	1.34-1.40	2.41-2.48	0.48-0.51	0.80-0.91	1.30-1.39
HTR noY	0.01-0.01	0.20-0.20	0.40-0.40	0.01-0.01	0.18-0.18	0.36-0.36	0.01-0.01	0.11-0.11	0.23-0.23
HTR allele	0.01-0.01	0.23-0.27	0.50-0.51	0.01-0.01	0.21-0.24	0.45-0.46	0.01-0.01	0.13-0.15	0.26-0.28
HTR FB	0.01-0.01	0.23-0.27	0.50-0.51	0.01-0.01	0.21-0.24	0.45-0.46	0.01-0.01	0.13-0.15	0.26-0.27
HTR pen	0.01-0.01	0.23-0.26	0.50-0.50	0.01-0.01	0.21-0.23	0.45-0.46	0.01-0.01	0.13-0.14	0.26-0.27
KLD allele	0.27-0.52	0.06-0.14	0.13-0.21	0.05-0.05	0.04-0.05	0.05-0.15	0.05-0.08	0.04-0.08	0.08-0.35
KLD FB	0.27-0.53	0.06-0.14	0.13-0.21	0.05-0.05	0.04-0.05	0.05-0.21	0.06-0.08	0.04-0.08	0.08-0.39
KLD pen	0.15-0.52	0.09-0.15	0.10-0.16	0.05-0.06	0.04-0.05	0.07-0.13	0.05-0.09	0.06-0.08	0.09-0.23

G)

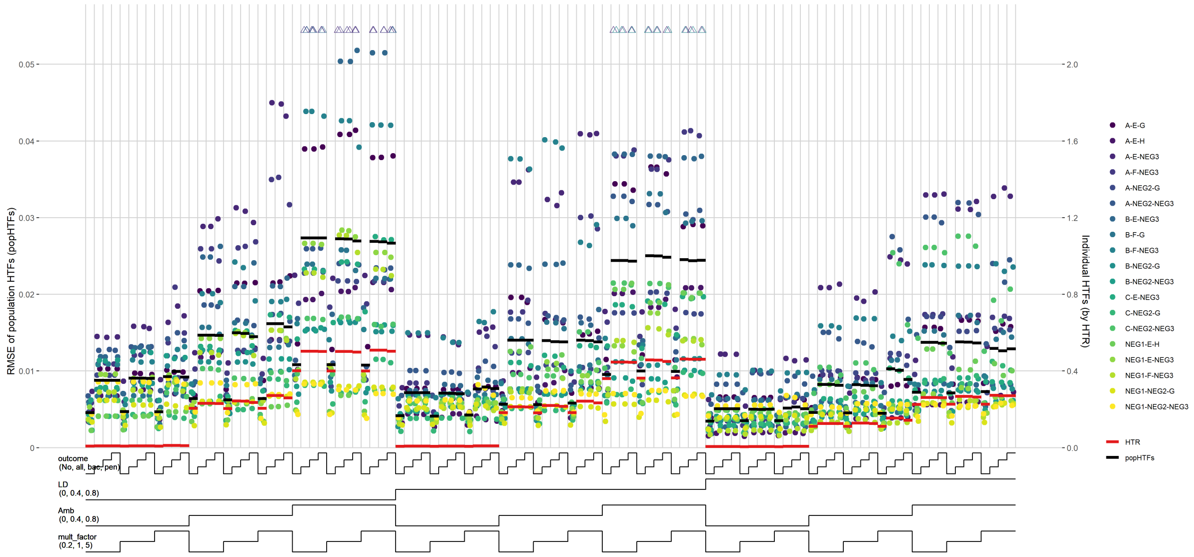

RMSE noY	0.46-0.47	0.62-0.64	1.08-1.08	0.41-0.43	0.56-0.58	0.95-1.00	0.35-0.35	0.45-0.46	0.72-0.73
RMSE allele	0.88-0.93	1.47-1.62	2.69-2.74	0.71-0.77	1.40-1.40	2.44-2.50	0.50-0.52	0.82-1.03	1.29-1.38
RMSE FB	0.88-0.99	1.47-1.62	2.69-2.74	0.71-0.79	1.38-1.40	2.44-2.50	0.50-0.53	0.81-1.01	1.26-1.38
RMSE pen	0.88-0.92	1.45-1.58	2.67-2.74	0.70-0.77	1.38-1.40	2.43-2.48	0.50-0.51	0.81-0.89	1.29-1.37
HTR noY	0.01-0.01	0.20-0.21	0.40-0.40	0.01-0.01	0.18-0.18	0.36-0.36	0.01-0.01	0.11-0.11	0.23-0.23
HTR allele	0.01-0.01	0.23-0.27	0.50-0.51	0.01-0.01	0.21-0.24	0.45-0.46	0.01-0.01	0.13-0.15	0.26-0.27
HTR FB	0.01-0.01	0.23-0.27	0.50-0.51	0.01-0.01	0.21-0.24	0.45-0.46	0.01-0.01	0.13-0.15	0.26-0.27
HTR pen	0.01-0.01	0.23-0.26	0.50-0.50	0.01-0.01	0.21-0.23	0.45-0.46	0.01-0.01	0.13-0.14	0.26-0.27
KLD allele	0.16-0.63	0.04-0.15	0.18-0.47	0.04-0.06	0.04-0.05	0.04-0.17	0.08-0.08	0.05-0.06	0.14-0.25
KLD FB	0.03-0.60	0.04-0.15	0.18-0.51	0.03-0.06	0.04-0.05	0.05-0.18	0.07-0.09	0.05-0.06	0.14-0.28
KLD pen	0.03-0.41	0.06-0.15	0.18-0.47	0.03-0.06	0.04-0.05	0.06-0.19	0.06-0.08	0.06-0.08	0.15-0.20

Figure S4: HTF estimation. Nested-loop plot of RMSEs for HTFs and HTR measure for all sub-scenarios of A) scenario 002, B) scenario 011, C) scenario 012, D) scenario 003, E) scenario 013, F) scenario 001* G) scenario 002*. Explanation about the Nested-loop plots is given in the legend of Figure 2. The horizontal bold black lines represent the RMSE of the popHTFs (with values on the left y-axis), while the bold red lines represent the HTR measure of the individual HTFs (with values on the right y-axis). The table below the graph shows mean RMSE values of these popHTFs and the HTR measures, as well as the KLD ratios between the no-outcome model and the three outcome models. All values in the table are the range (min-max) of values for the three sub-scenarios above. A ratio > 1 indicates higher similarity of the HTFs of the outcome models with the truth than the no-outcome model. Mean RMSE values in the table have been multiplied by 100 for readability.

A)

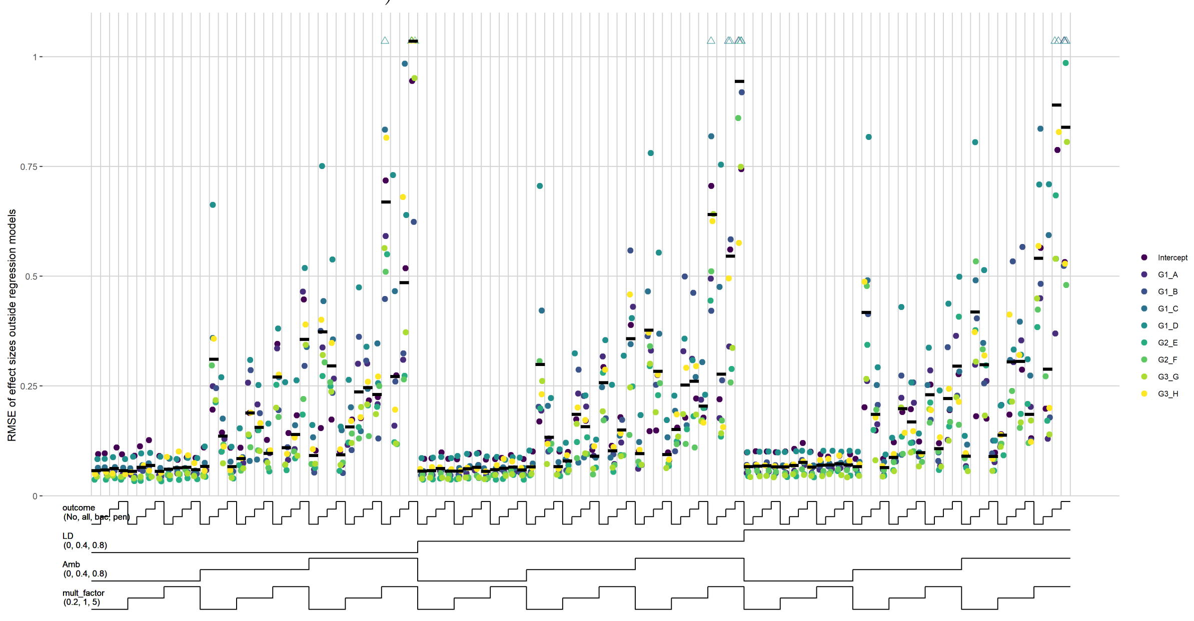

RMSE noY	5.58-6.06	6.72-27.08	9.19-66.94	5.62-5.94	6.60-25.80	9.60-64.08	6.62-7.00	6.66-23.04	9.02-54.11
RMSE allele	5.88-6.50	10.94-31.11	23.67-37.35	5.76-6.28	10.25-29.92	25.26-37.75	6.77-7.04	10.74-41.79	28.82-41.90
RMSE FB	6.20-6.91	13.60-15.58	24.67-48.55	6.24-6.53	13.33-15.73	26.09-54.57	6.86-7.63	16.83-22.15	29.87-89.00
RMSE pen	5.55-5.94	6.66-35.63	9.33-106.67	5.57-5.90	6.64-35.81	9.40-94.40	6.56-7.03	6.44-29.57	8.96-83.93

B)

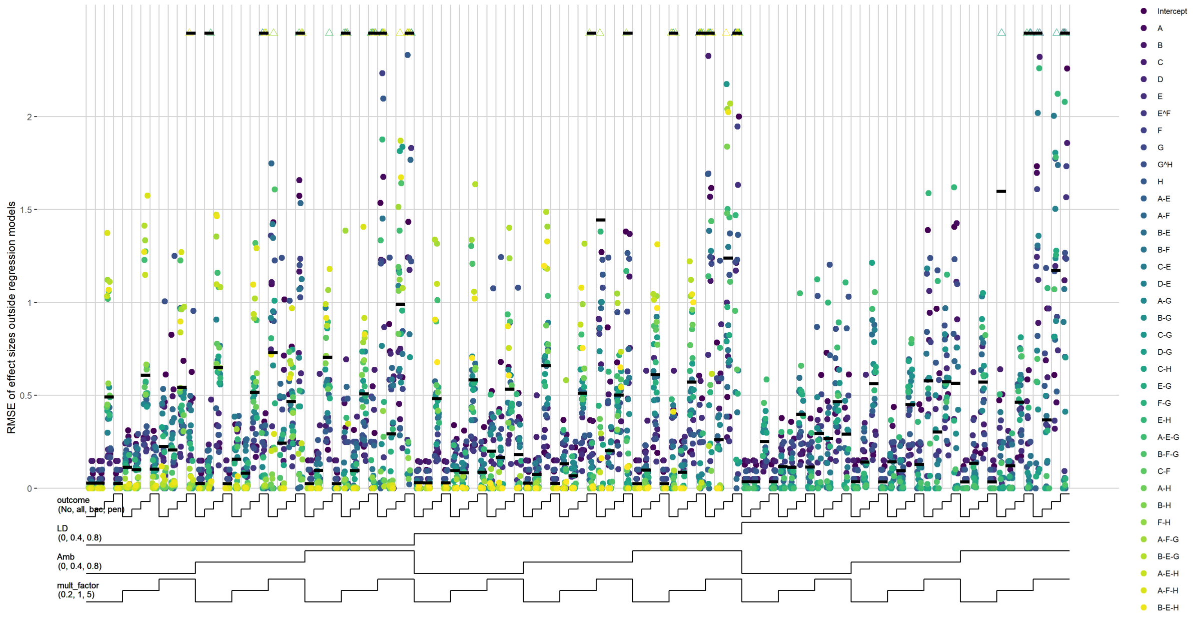

RMSE noY	2.76-22.50	2.67-72.92	2.52-3.7e03	2.97-19.91	2.75-144.36	2.54-2.4e03	3.54-29.52	3.47-57.78	3.42-1.2e05
RMSE allele	2.70-20.63	8.10-2.6e12	9.45-29.22	2.84-16.68	6.67-20.25	8.59-26.20	3.48-26.79	9.51-30.33	12.11-36.80
RMSE FB	49.07-60.86	46.65-65.09	50.76-99.04	48.24-58.29	50.13-65.90	57.19-123.96	25.23-46.53	45.07-57.32	46.35-117.33
RMSE pen	2.61-403.79	2.53-6.5e15	2.52-5.1e20	2.82-18.19	2.57-1e14	2.54-2e18	3.54-29.06	3.44-56.52	3.41-1.7e23

Figure S5: Substantive model regression coefficients, scenario 001. Nested-loop plots of RMSE values for estimated regression coefficients for all sub-scenarios of default scenario 001, containing HTF and effect size set 1. Two substantive models are run with different sets of predictors: A) only alleles as predictor and B) all candidate list predictors as predictor. Explanation about the Nested-loop plots is given in the legend of Figure 2. Here, the coloured dots are either alleles (single letters) or haplotypes (combination of alleles separated by a '-'). The gene origin of each allele is conform Table 1. The horizontal bold black lines represent the RMSE of the effect sizes, with its values displayed in the table below the graph. These RMSE values give the range (min-max) for the three sub-scenarios above. Mean RMSE values have been multiplied by 100 for readability.

A)

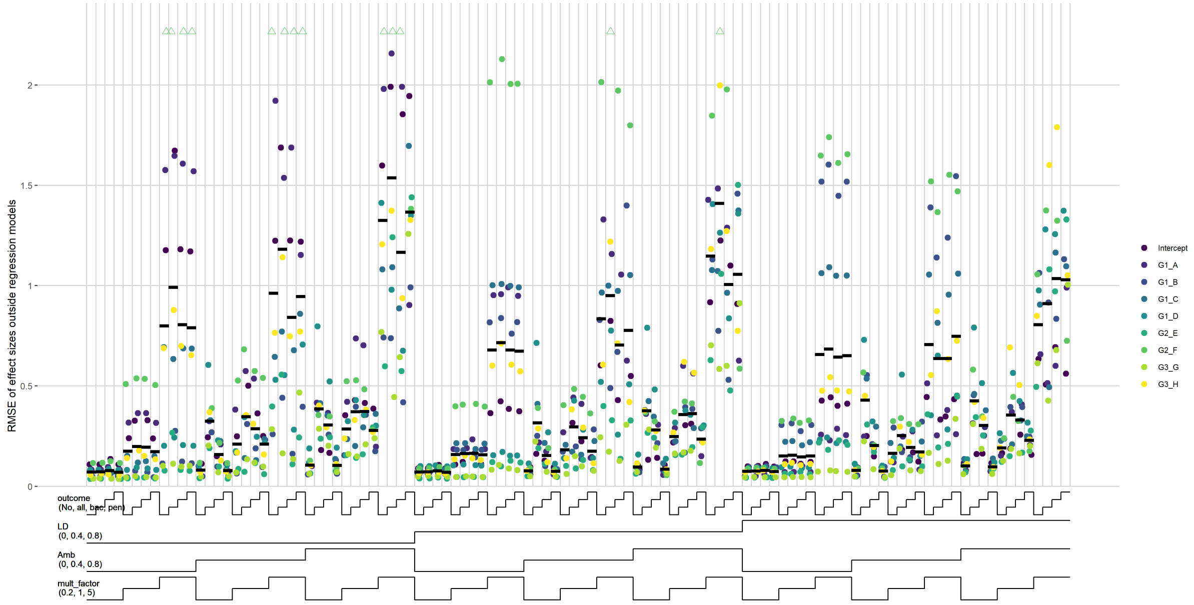

RMSE noY	7.12-80.00	8.07-96.31	10.52-132.52	7.12-67.94	7.70-83.54	9.67-114.74	7.47-65.78	7.96-70.67	10.19-80.65
RMSE allele	7.26-99.21	32.53-118.14	37.17-153.82	7.26-71.68	29.64-95.13	35.75-141.12	7.64-68.45	25.34-63.77	35.61-91.14
RMSE FB	7.89-80.54	15.87-84.26	30.64-116.62	7.68-68.02	15.40-70.42	28.11-100.60	8.02-64.43	19.41-63.74	30.41-103.56
RMSE pen	7.08-79.10	7.86-94.57	10.42-136.67	7.08-67.44	7.51-77.72	8.59-105.66	7.42-65.20	7.63-74.81	9.77-102.99

B)

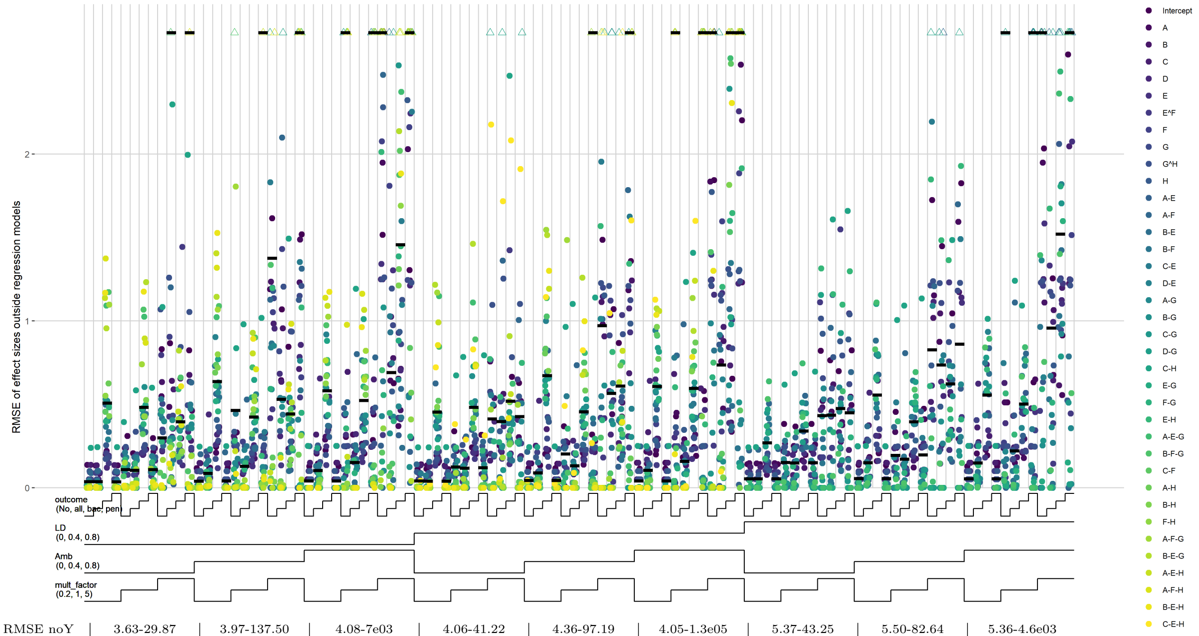

RMSE noY	3.63-29.87	3.97-137.50	4.08-7e03	4.06-41.22	4.36-97.19	4.05-1.3e05	5.37-43.25	5.50-82.64	5.36-4.6e03
RMSE allele	3.62-1.9e04	8.65-53.04	10.32-68.99	3.98-39.69	8.86-56.58	10.37-73.56	5.32-43.49	14.99-73.58	14.79-95.71
RMSE FB	39.50-50.69	42.29-63.67	52.34-145.57	45.33-51.84	45.48-67.23	59.47-341.74	26.91-47.41	39.57-62.16	50.19-152.04
RMSE pen	3.56-3e09	4.02-4.2e17	4.08-3.8e19	3.97-42.68	4.35-2.3e15	4.12-5e19	5.36-44.89	5.44-86.12	5.30-1.5e19

C)

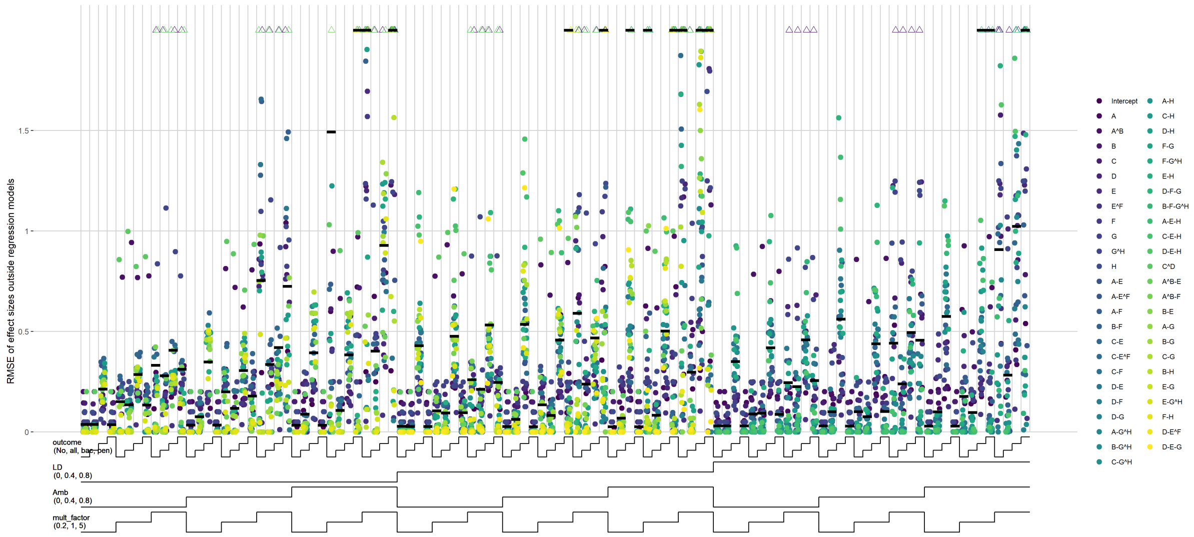

RMSE noY	3.65-33.10	3.34-75.32	3.31-323.18	2.81-26.01	2.66-58.99	2.54-3.9e+03	2.97-24.52	2.94-44.15	2.91-90.80
RMSE allele	3.73-27.98	7.57-33.54	8.80-40.21	2.65-21.13	6.17-23.80	6.86-29.73	2.97-22.52	7.51-23.90	9.63-28.30
RMSE FB	21.26-40.66	30.49-41.97	38.29-92.85	42.93-53.18	45.70-53.51	50.18-3.8e+33	35.05-45.85	43.84-56.13	57.45-2.2e+14
RMSE pen	3.59-31.12	3.40-72.43	3.31-3e+19	2.67-24.59	2.57-2.1e+07	2.56-1.1e+25	2.96-25.53	2.92-45.58	2.90-5.7e+16

D)

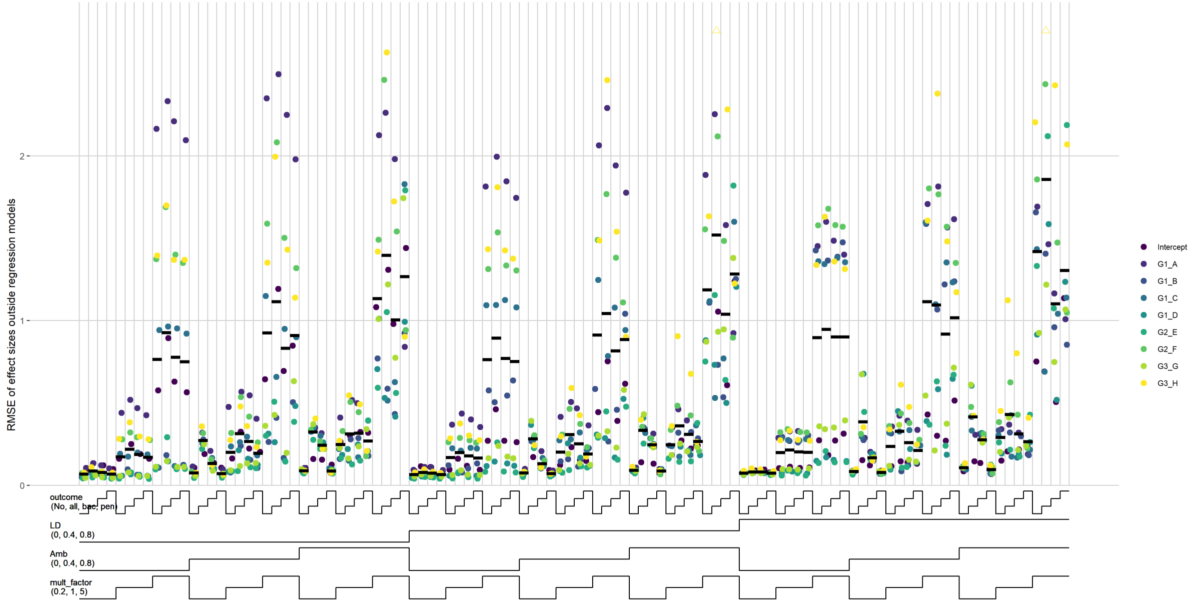

RMSE noY	6.89-76.43	7.58-92.50	8.90-113.42	6.59-76.30	7.52-91.30	9.13-118.68	7.40-89.66	8.40-111.49	10.64-141.89
RMSE allele	8.65-92.73	27.22-111.45	31.26-139.58	7.78-89.41	28.29-104.26	33.34-152.05	8.19-94.66	32.82-109.50	41.53-185.69
RMSE FB	7.84-77.82	13.24-83.15	24.39-100.43	7.38-77.10	13.22-81.65	24.65-103.85	8.19-90.10	16.71-91.79	27.59-110.21
RMSE pen	6.80-75.00	7.12-90.99	8.76-126.75	6.51-75.16	7.16-88.57	8.69-128.29	7.35-90.11	7.86-101.73	9.50-130.49

E)

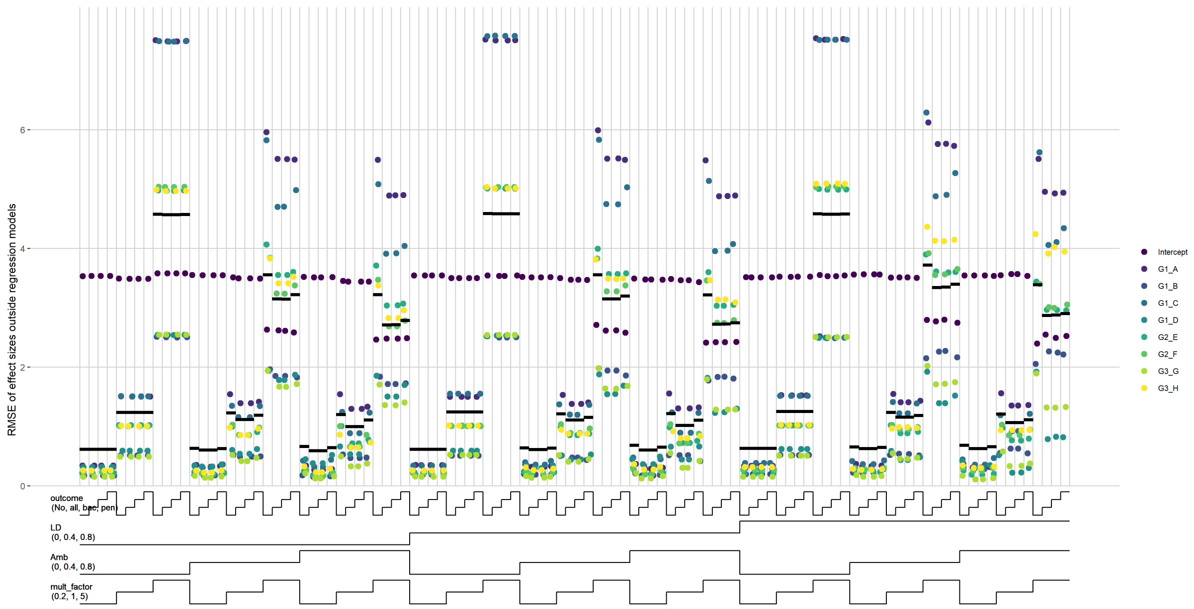

RMSE noY	61.65-457.91	63.01-355.62	66.31-322.38	61.67-459.05	64.17-355.90	68.38-321.83	63.18-458.45	65.62-372.23	68.55-338.86
RMSE allele	61.55-457.06	60.53-315.01	59.39-271.37	61.55-458.56	61.17-315.02	60.51-272.61	63.04-457.77	62.96-334.25	62.78-287.06
RMSE FB	61.55-457.06	60.53-314.89	59.39-271.51	61.55-458.56	61.17-314.94	60.51-272.94	63.04-457.77	62.96-334.97	62.78-287.85
RMSE pen	61.58-457.36	62.92-322.18	64.57-278.74	61.58-458.75	63.46-320.04	65.17-274.77	63.09-458.19	64.75-339.83	65.98-290.37

F)

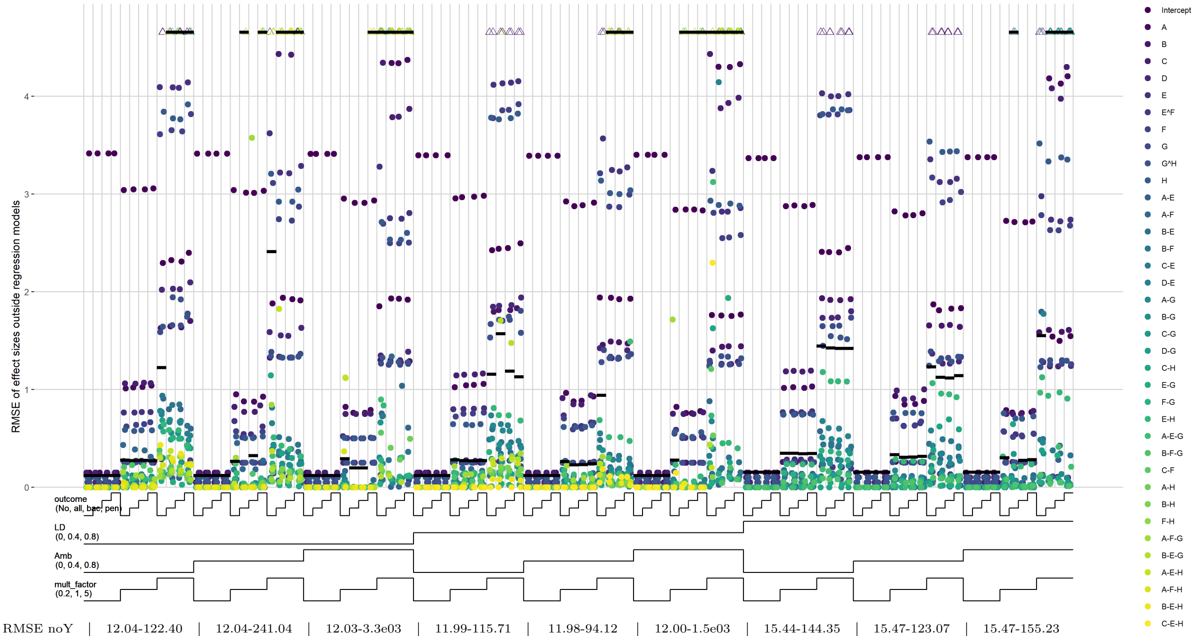

RMSE noY	12.04-122.40	12.04-241.04	12.03-3.3e03	11.99-115.71	11.98-94.12	12.00-1.5e03	15.44-144.35	15.47-123.07	15.47-155.23
RMSE allele	12.04-1.3e08	12.04-3.3e10	12.03-1.8e17	11.99-157.17	11.98-9.2e06	12.00-1.3e28	15.44-142.61	15.47-112.55	15.47-2.1e19
RMSE FB	12.04-1.3e11	12.04-1.7e15	12.03-1.4e17	11.99-118.80	11.98-1.1e07	12.00-4.3e19	15.44-142.07	15.47-111.74	15.47-1.1e17
RMSE pen	12.04-1.9e09	12.04-1.2e15	12.03-1.5e22	11.99-112.99	11.98-4.7e14	12.00-7.6e17	15.44-142.10	15.47-114.08	15.47-1.8e17

G)

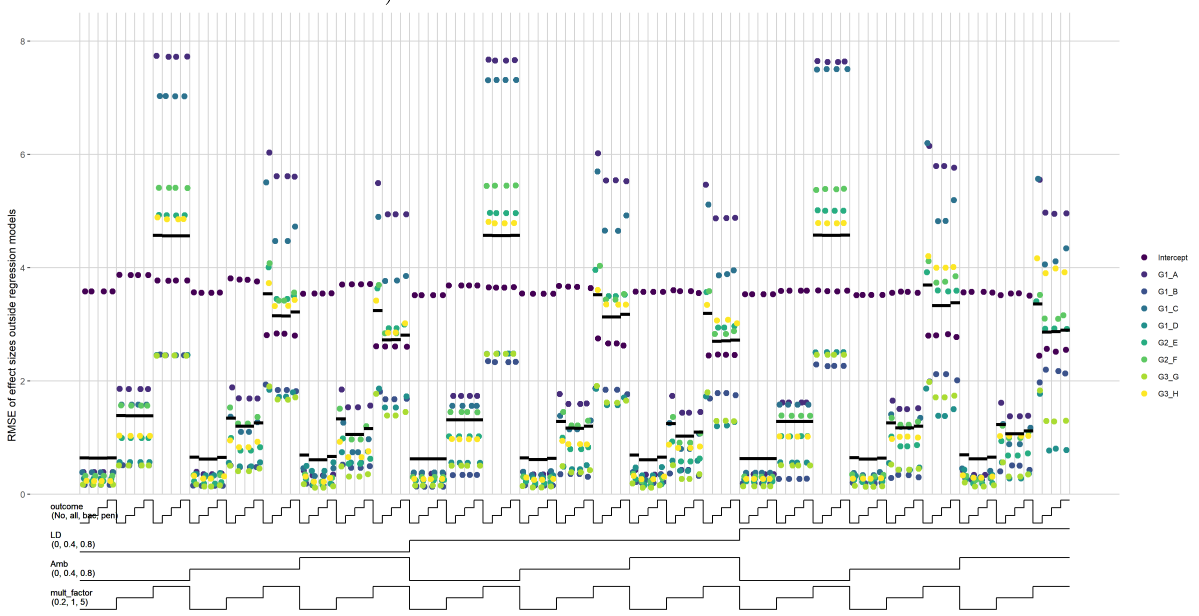

RMSE noY	64.32-457.39	65.74-354.11	69.28-324.76	62.66-457.56	64.56-352.56	69.39-319.64	63.33-457.79	64.79-369.56	69.98-336.17
RMSE allele	64.23-456.64	62.25-315.22	61.23-272.88	62.57-457.09	61.39-313.32	61.10-270.51	63.21-457.24	62.43-333.21	62.64-286.76
RMSE FB	64.23-456.62	62.25-315.19	61.23-273.09	62.57-457.08	61.39-313.27	61.10-271.08	63.21-457.22	62.43-333.46	62.64-287.49
RMSE pen	64.26-456.70	65.12-322.01	66.81-281.25	62.60-457.34	63.73-318.04	65.76-272.20	63.25-457.70	64.15-338.49	65.80-289.80

H)

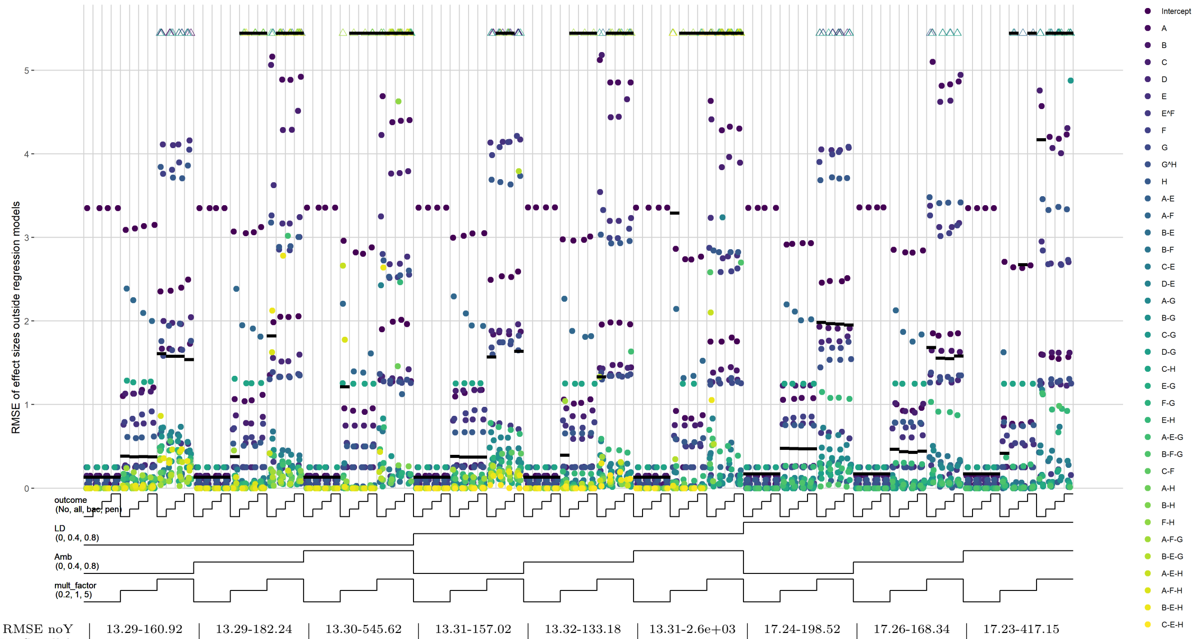

RMSE noY	13.29-160.92	13.29-182.24	13.30-545.62	13.31-157.02	13.32-133.18	13.31-2.6e+03	17.24-198.52	17.26-168.34	17.23-417.15
RMSE allele	13.32-158.02	13.29-2.3e+12	13.30-5.9e+17	13.31-3.1e+04	13.32-2.6e+15	13.31-4.2e+18	17.24-196.89	17.26-155.49	17.23-3.8e+17
RMSE FB	13.30-157.81	13.29-2.2e+14	13.30-4.5e+21	13.31-1.4e+04	13.32-4e+14	13.31-5.6e+19	17.24-196.47	17.26-154.98	17.23-1.3e+19
RMSE pen	13.29-154.06	13.29-6.8e+15	13.30-1.6e+21	13.31-163.83	13.32-1.2e+15	13.31-1.1e+19	17.24-195.39	17.26-158.05	17.23-7.8e+16

Figure S6: substantive model regression coefficients Nested-loop plots of RMSE values for estimated regression coefficients for all sub-scenarios of A) scenario 002 with only alleles as predictor, B) scenario 002 with all candidate list haplotypes as predictors, C) scenario 011 with all candidate list haplotypes as predictors D) scenario 012 with only alleles as predictor E) scenario 001* with only alleles as predictors F) scenario 001* with all candidate list haplotypes as predictors G) scenario 002* with only alleles as predictor H) scenario 002* with all candidate list haplotypes as predictors. Explanation about the Nested-loop plots is given in the legend of Figure 2. Here, the coloured dots are either alleles (single letters) or haplotypes (combination of alleles separated by a '-'). The gene origin of each allele is conform Table 1. The horizontal bold black lines represent the RMSE of the effect sizes, with its values displayed in the table below the graph. These RMSE values give the range (min-max) for the three sub-scenarios above. Mean RMSE values have been multiplied by 100 for readability.

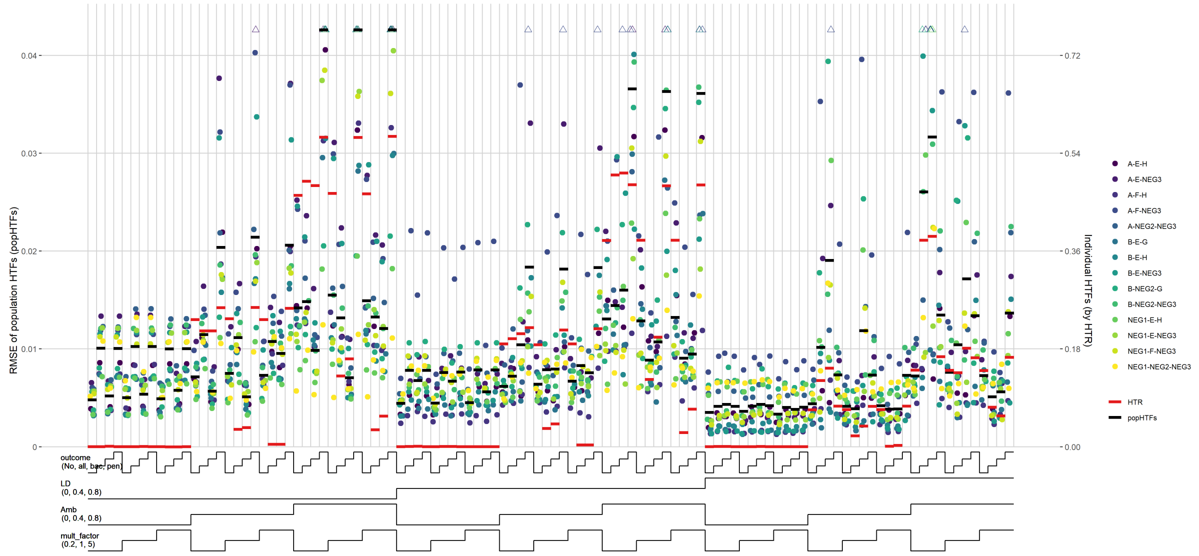

RMSE noY	0.48-0.50	0.71-0.75	1.42-1.55	0.44-0.45	0.62-0.67	1.29-1.32	0.33-0.36	0.41-0.44	0.72-0.76
RMSE allele	1.00-1.02	1.08-1.15	1.32-1.48	0.76-0.79	0.72-0.83	0.89-1.44	0.40-0.42	0.38-1.02	0.51-2.61
RMSE FB	0.52-0.58	0.51-0.95	0.71-1.21	0.56-0.68	0.76-1.04	0.95-1.60	0.38-0.43	0.39-1.90	0.56-3.17
RMSE pen	1.00-1.03	2.04-2.14	4.36-4.40	0.76-0.79	1.82-1.84	3.61-3.66	0.40-0.42	0.73-0.73	1.34-1.37
HTR noY	0.00-0.00	0.23-0.24	0.46-0.47	0.00-0.00	0.19-0.19	0.38-0.38	0.00-0.00	0.07-0.07	0.14-0.14
HTR allele	0.00-0.00	0.00-0.21	0.03-0.49	0.00-0.00	0.00-0.20	0.03-0.50	0.00-0.00	0.00-0.12	0.07-0.38
HTR FB	0.00-0.00	0.00-0.21	0.06-0.48	0.00-0.00	0.00-0.21	0.07-0.50	0.00-0.00	0.00-0.14	0.06-0.39
HTR pen	0.00-0.00	0.25-0.26	0.57-0.57	0.00-0.00	0.21-0.22	0.48-0.48	0.00-0.00	0.07-0.08	0.16-0.17
KLD allele	0.21-0.32	0.41-1.92	0.41-4.61	0.01-0.02	0.21-0.39	0.17-1.44	0.05-0.07	0.02-0.64	0.03-1.07
KLD FB	0.50-0.68	0.79-3.63	0.43-2.87	0.02-0.37	0.08-0.36	0.11-1.42	0.09-0.22	0.01-1.49	0.01-0.83
KLD pen	0.21-0.31	0.14-0.29	0.08-0.21	0.01-0.02	0.02-0.03	0.03-0.09	0.05-0.07	0.07-0.08	0.06-0.17

Figure S7: HTF estimation, scenario A. Nested-loop plot of RMSEs for HTFs and haplotype reconstruction (HTR) measure for all sub-scenarios of the proof-of-concept study without haplotype effects. Explanation about the Nested-loop plots is given in the legend of Figure 2. The horizontal bold black lines represent the RMSE of the popHTFs (with values on the left y-axis), while the bold red lines represent the HTR measure of the individual HTFs (with values on the right y-axis). The table below the graph shows mean RMSE values of these popHTFs and the HTR measure, as well as the Kullback-Leibler divergence (KLD) ratios between the no-outcome model and the three outcome models. All values in the table give ranges (min-max) of values for the three sub-scenarios above. A ratio > 1 indicates higher similarity of the HTFs of the outcome models with the truth than the no-outcome model. Mean RMSE values in the table have been multiplied by 100 for readability.

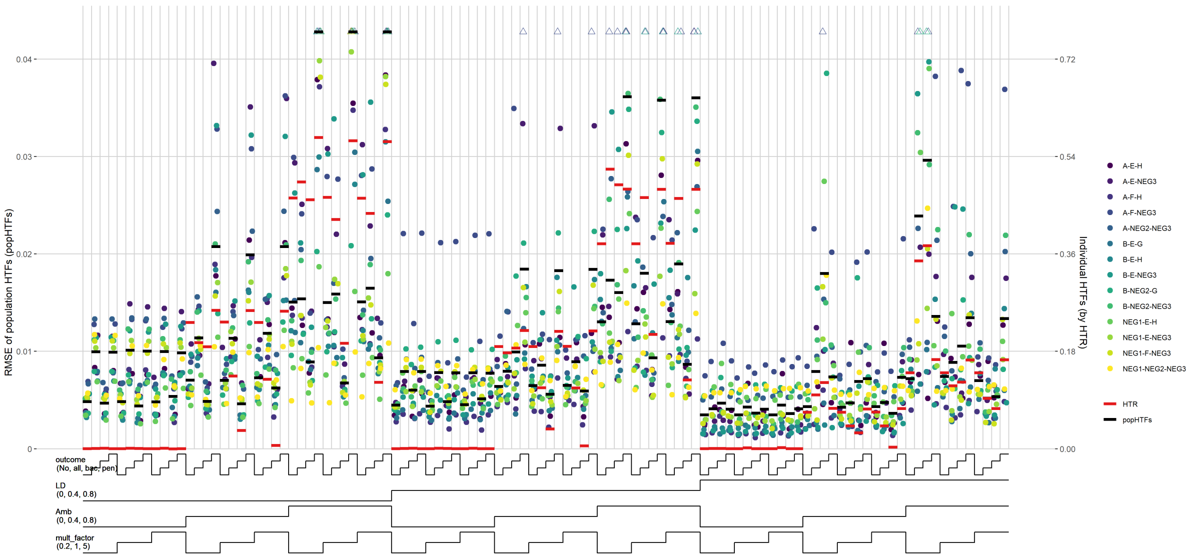

RMSE noY	0.48-0.48	0.70-0.73	1.50-1.51	0.44-0.45	0.64-0.65	1.28-1.31	0.35-0.35	0.43-0.43	0.70-0.74
RMSE allele	1.00-1.01	1.13-1.18	1.54-1.65	0.78-0.79	0.80-0.89	1.73-1.90	0.41-0.45	0.47-0.79	0.52-2.39
RMSE FB	0.44-0.54	0.46-0.62	0.68-0.93	0.48-0.60	0.56-0.99	0.87-1.60	0.36-0.47	0.36-1.80	0.54-2.96
RMSE pen	0.99-1.00	1.99-2.08	4.42-4.49	0.78-0.79	1.83-1.84	3.58-3.61	0.41-0.41	0.72-0.74	1.34-1.36
HTR noY	0.00-0.00	0.23-0.23	0.46-0.46	0.00-0.00	0.19-0.19	0.38-0.38	0.00-0.00	0.07-0.07	0.14-0.14
HTR allele	0.00-0.00	0.13-0.20	0.42-0.49	0.00-0.00	0.11-0.18	0.46-0.52	0.00-0.00	0.04-0.10	0.09-0.35
HTR FB	0.00-0.00	0.01-0.19	0.12-0.46	0.00-0.00	0.01-0.19	0.13-0.49	0.00-0.00	0.00-0.12	0.07-0.38
HTR pen	0.00-0.00	0.25-0.26	0.57-0.58	0.00-0.00	0.22-0.22	0.48-0.48	0.00-0.00	0.07-0.07	0.16-0.16
KLD allele	0.14-0.18	0.10-0.16	0.11-0.15	0.02-0.02	0.11-0.21	0.07-0.12	0.03-0.04	0.02-0.10	0.02-0.63
KLD FB	0.24-0.89	0.74-5.96	0.35-1.11	0.04-0.31	0.06-0.71	0.07-0.39	0.03-0.34	0.01-1.93	0.01-0.44
KLD pen	0.18-0.26	0.18-0.27	0.07-0.14	0.02-0.03	0.02-0.03	0.06-0.09	0.05-0.06	0.05-0.07	0.09-0.11

Figure S8: HTF estimation, scenario B. Nested-loop plot of RMSEs for HTFs and HTR measure for all sub-scenarios of the proof-of-concept study with haplotype effects. Each column of points separated by vertical grid lines depicts a different set of parameter, where the lines at the bottom of the graph define these parameter sets. Explanation about the Nested-loop plots is given in the legend of Figure 2. The horizontal bold black lines represent the RMSE of the popHTFs (with values on the left y-axis), while the bold red lines represent the HTR measure of the individual HTFs (with values on the right y-axis). The table below the graph shows mean RMSE values of these popHTFs and the HTR measures, as well as the KLD ratios between the no-outcome model and the three outcome models. All values in the table give ranges (min-max) of values for the three sub-scenarios above. A ratio > 1 indicates higher similarity of the HTFs of the outcome models with the truth than the no-outcome model. Mean RMSE values in the table have been multiplied by 100 for readability.

A)

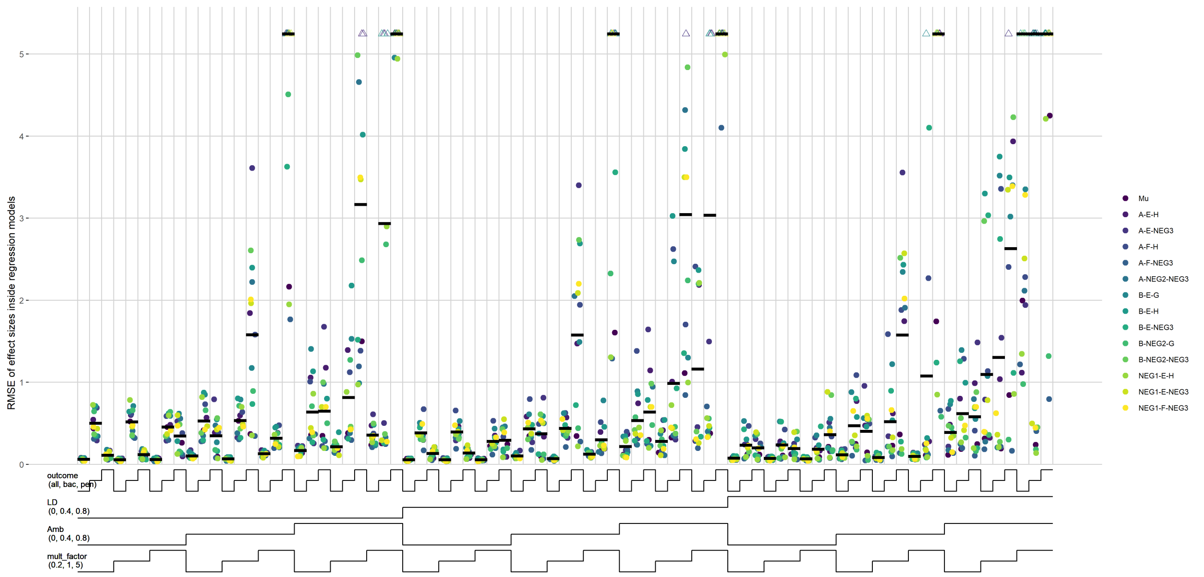

RMSE allele	5.58-6.04	6.66-13.00	16.82-35.54	5.64-5.74	6.93-12.36	21.76-116.23	6.80-7.43	8.39-11.71	38.85-573.13
RMSE FB	45.48-51.69	31.76-53.47	63.78-293.34	27.89-39.39	29.97-43.76	53.28-303.55	18.35-23.59	47.21-107.71	61.92-600.21
RMSE pen	11.14-34.51	34.87-796.90	64.91-1.6e03	13.28-29.43	37.35-791.90	63.62-1.5e03	19.35-36.07	40.28-779.70	57.91-1.3e03

B)

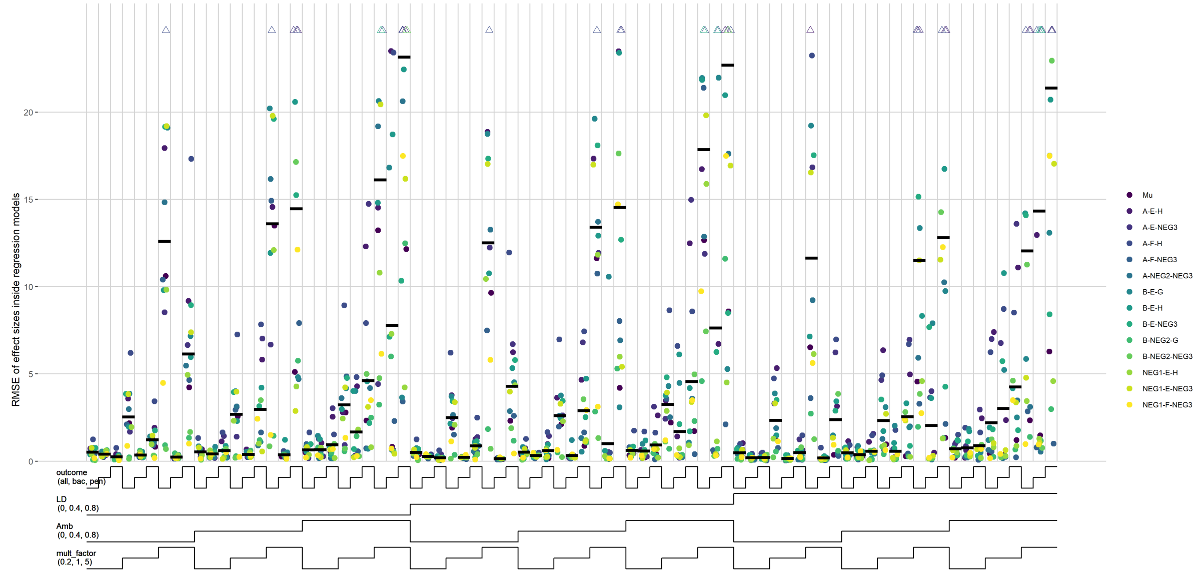

RMSE allele	51.49-1.3e03	52.79-1.4e03	65.24-1.6e03	50.79-1.3e03	52.16-1.3e03	62.74-1.8e03	48.19-1.2e03	48.79-1.2e03	71.14-1.2e03
RMSE FB	23.74-40.28	37.21-41.24	65.77-779.01	15.29-27.91	32.96-100.95	58.60-762.94	15.90-19.51	38.14-205.42	76.22-1.4e03
RMSE pen	25.40-614.21	60.58-1.4e03	93.98-2.3e03	19.90-430.18	61.23-1.5e03	93.78-2.3e03	21.52-238.54	56.75-1.3e03	89.65-2.1e03

Figure S9: Outcome regression coefficient Nested-loop plots of RMSE values for estimated regression coefficients for all sub-scenarios of the proof-of-concept study A) scenario A (without haplotype effects) and B) scenario B (with haplotype effects). Explanation about the Nested-loop plots is given in the legend of Figure 2. Here only three outcome models are tested: allelic model (lowest line), forward-backward model (middle step) and penalized regression model (upper line). The horizontal bold black lines represent the RMSE of the effect sizes, with its values displayed in the table below the graph. These RMSE values give the range (min-max) for the three sub-scenarios above. Mean RMSE values have been multiplied by 100 for readability.

A)

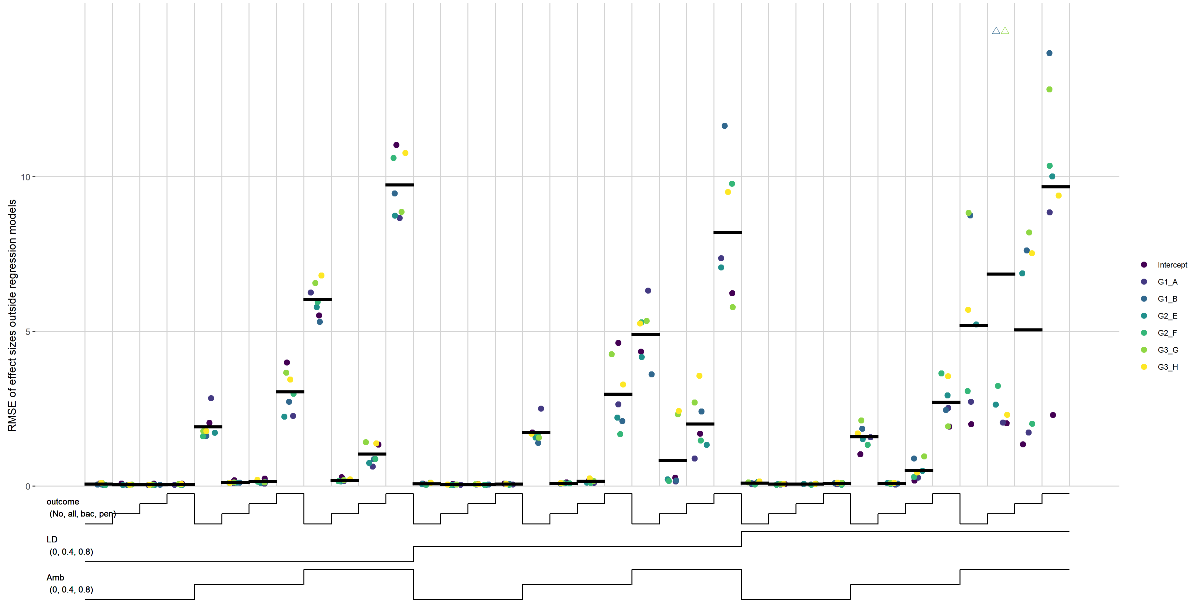

RMSE noY	4.66-6.88	8.91-191.47	25.13-602.91	4.84-7.31	8.76-173.36	20.77-490.63	6.56-9.76	10.17-159.21	22.21-518.76
RMSE allele	4.52-4.86	5.50-11.95	9.71-18.68	4.90-4.99	5.59-10.94	17.67-81.94	6.24-6.60	7.28-13.54	39.48-685.31
RMSE FB	4.57-4.87	5.97-14.55	19.76-103.74	4.90-5.30	9.19-15.67	20.51-201.14	6.56-6.70	12.91-50.40	34.91-504.73
RMSE pen	4.54-5.64	12.63-304.86	38.93-973.50	4.83-6.34	13.34-297.43	32.76-819.92	6.57-8.53	13.36-270.86	38.53-967.68

B)

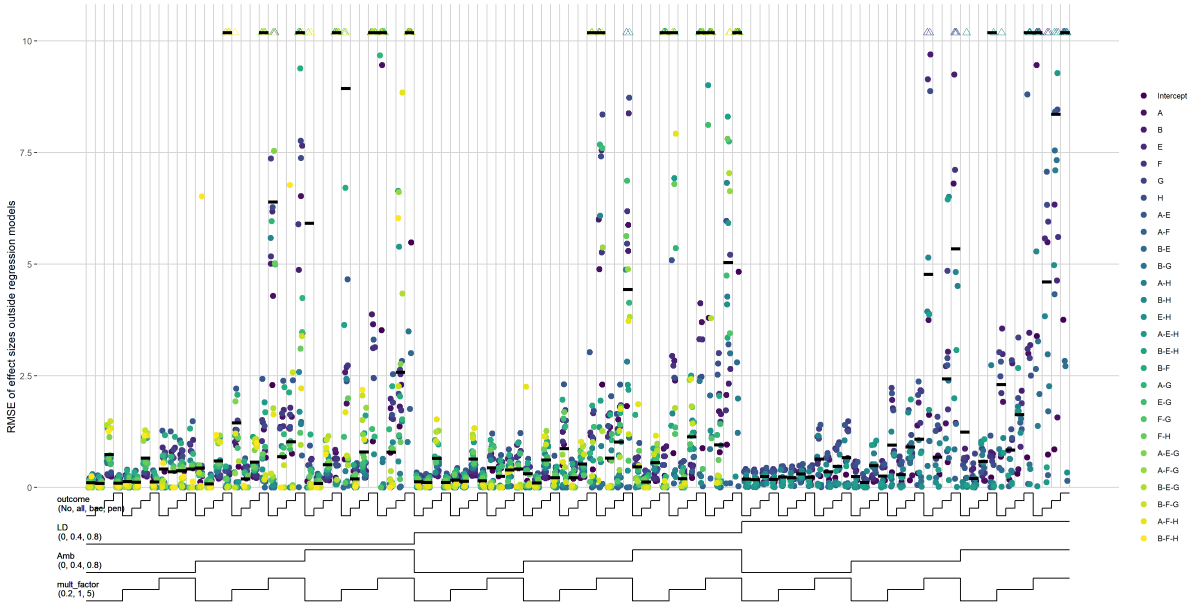

RMSE noY	10.48-41.21	43.13-639.21	591.37-4.8e03	12.29-43.98	30.54-6.9e04	45.73-8.7e03	18.44-63.55	24.22-477.33	123.92-1e03
RMSE allele	8.85-35.00	7.56-68.76	8.73-78.73	11.00-24.60	9.63-65.59	12.00-94.90	17.46-35.00	10.88-67.75	20.11-460.02
RMSE FB	37.46-73.21	56.28-101.91	50.77-257.96	39.18-65.03	51.99-101.26	55.35-503.69	24.14-46.99	48.68-243.19	58.14-835.96
RMSE pen	9.14-41.13	1.2e14-1.6e16	2e17-9.3e18	11.10-41.10	21.55-4.6e14	1.5e19-1.7e26	17.95-66.29	24.97-534.06	5.5e07-3.3e18

C)

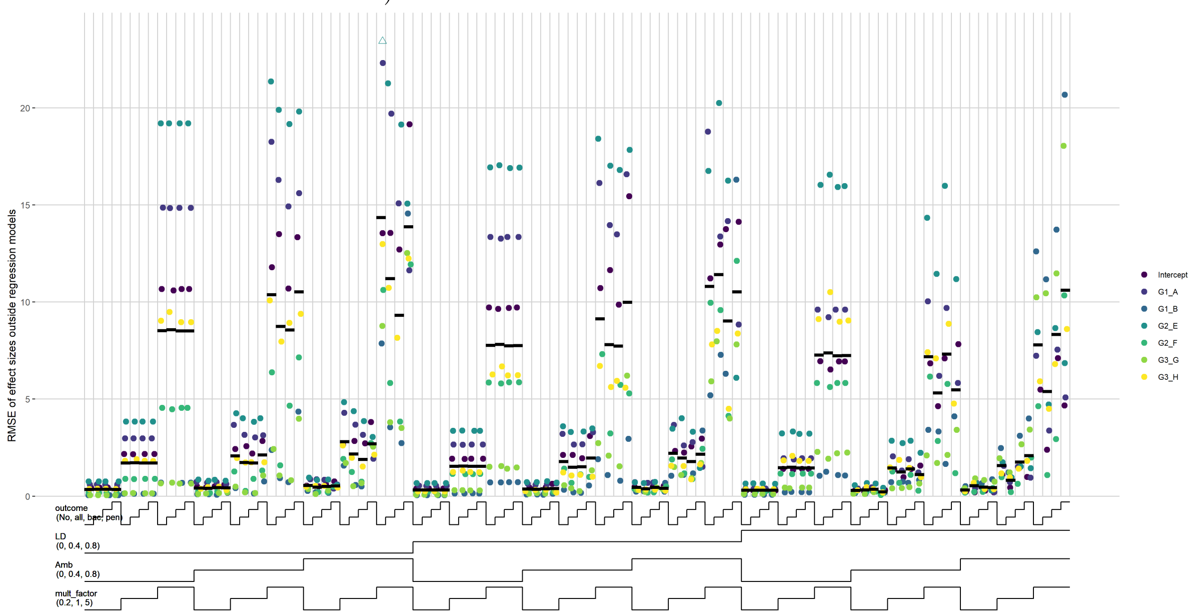

RMSE noY	35.44-852.94	42.41-1e03	56.14-1.4e03	32.04-777.12	37.03-913.81	45.37-1.1e03	30.72-726.96	30.18-718.99	32.29-779.91
RMSE allele	35.58-857.46	40.50-875.07	47.20-1.1e03	32.22-781.89	35.73-781.36	38.90-1.1e03	31.08-738.07	31.96-532.73	54.55-539.07
RMSE FB	35.54-851.45	44.10-856.15	50.75-931.53	32.12-774.48	38.24-774.02	45.81-902.37	30.78-723.08	36.27-732.23	47.13-832.97
RMSE pen	35.40-851.76	42.92-1.1e03	53.51-1.4e03	31.99-775.92	40.07-999.19	41.14-1.1e03	30.66-724.88	23.07-547.96	44.48-1.1e03

D)

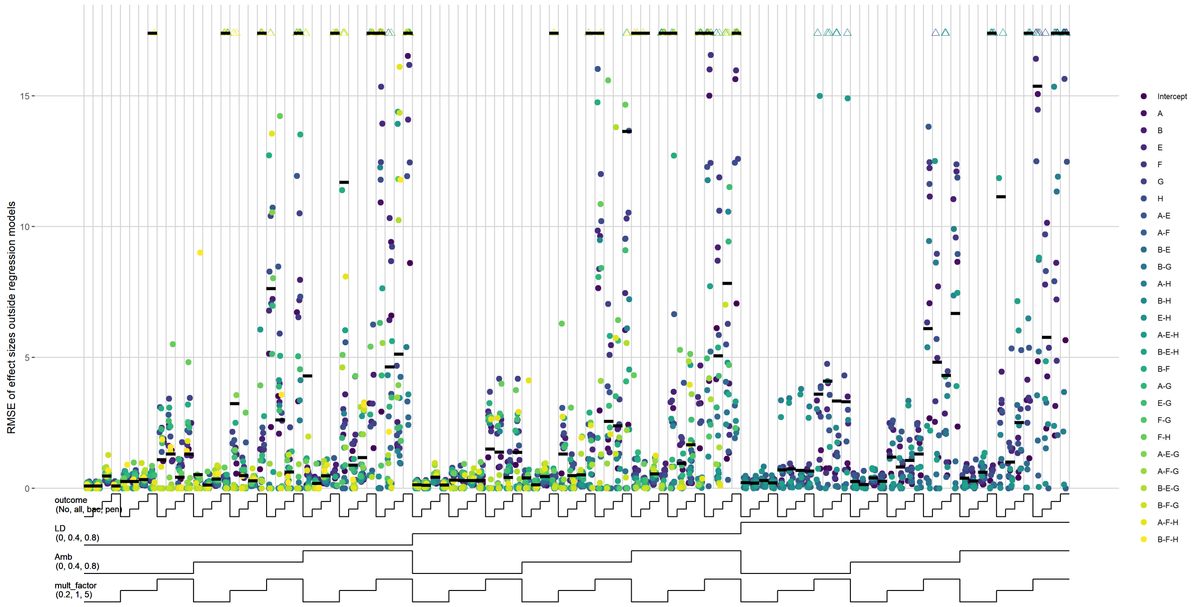

RMSE noY	8.98-109.13	52.29-763.11	429.06-1.4e04	13.14-149.54	39.10-3.1e03	2.3e03-5.4e03	21.39-359.47	26.05-610.58	38.85-1.5e03
RMSE allele	8.52-131.25	11.88-260.89	18.85-464.12	11.44-137.68	12.86-255.88	94.19-1.8e14	19.71-409.32	14.35-482.07	27.96-576.76
RMSE FB	33.47-46.56	28.78-62.32	47.62-512.89	29.37-41.77	44.47-238.06	54.58-783.53	29.84-333.74	39.58-431.05	61.44-1.8e10
RMSE pen	8.90-7.6e07	2.8e15-2.3e17	1.7e17-1.8e20	11.89-137.17	1.4e03-8e12	2.4e16-1.1e20	20.40-330.87	26.79-668.01	2.7e08-1.3e18

Figure S10: Substantive model regression coefficients. Nested-loop plots of RMSE values for estimated regression coefficients for all sub-scenarios of the proof-of-concept study. A) scenario A (without haplotype effects) with only alleles as predictor, B) scenario A with all candidate list haplotypes as predictors, C) scenario B (with haplotype effects) with only alleles as predictors and D) scenario B with all candidate list haplotypes as predictors. Explanation about the Nested-loop plots is given in the legend of Figure 2. Here, the coloured dots are either alleles (single letters) or haplotypes (combination of alleles separated by a '-'). The gene origin of each allele is conform Table 2. The horizontal bold black lines represent the RMSE of the effect sizes, with its values displayed in the table below the graph. These RMSE values give the range (min-max) for the three sub-scenarios above. Mean RMSE values have been multiplied by 100 for readability.

A)

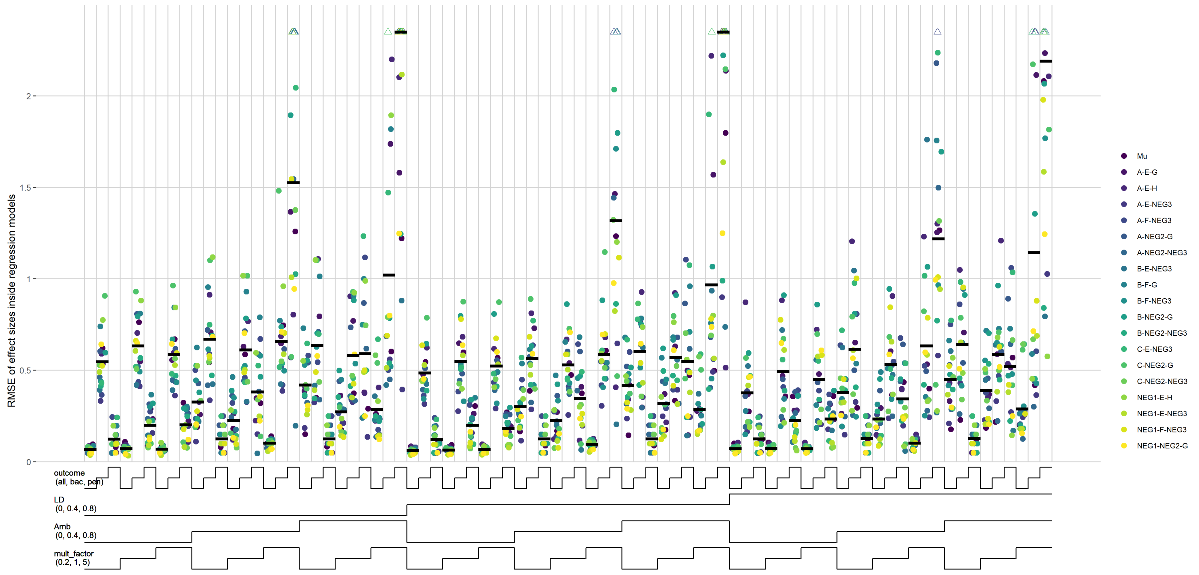

RMSE allele	6.69-7.11	10.18-32.70	27.27-42.03	6.16-6.80	9.60-30.16	28.51-41.62	7.14-7.37	10.22-37.94	28.94-45.04
RMSE FB	54.73-63.37	61.11-67.01	58.08-102.15	48.55-54.87	53.00-58.76	57.02-96.72	37.76-49.34	53.14-63.39	58.57-114.37
RMSE pen	12.35-20.17	12.53-152.54	12.54-271.23	12.19-19.98	12.57-131.84	12.57-239.48	12.59-23.46	12.72-121.85	12.73-219.01

B)

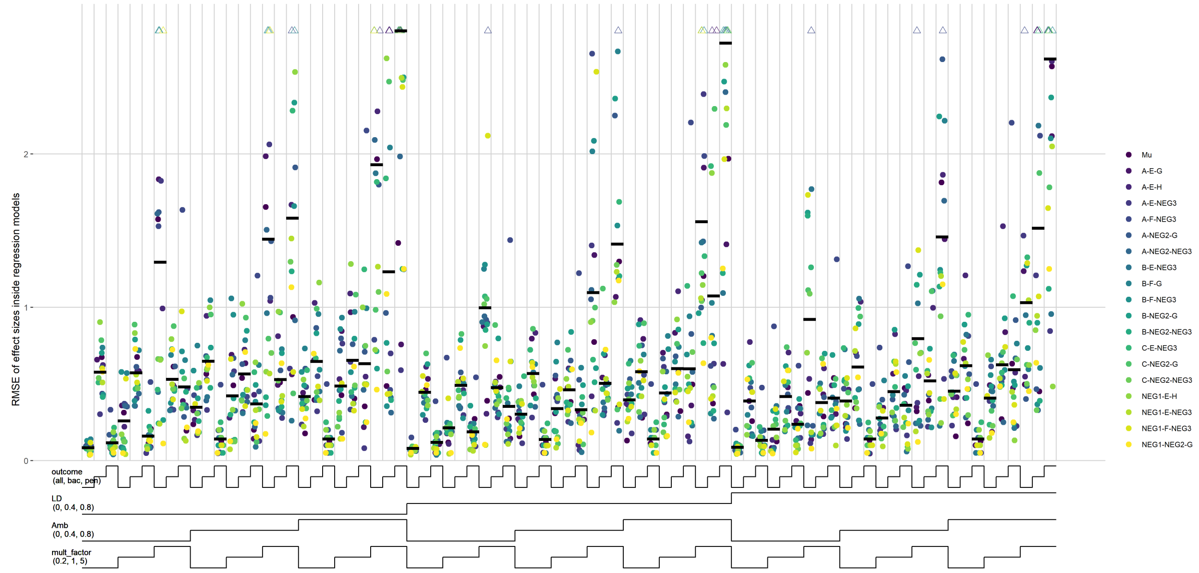

RMSE allele	8.46-129.45	34.83-144.43	41.77-193.03	7.90-99.60	30.20-109.60	39.54-155.86	8.65-92.06	27.82-79.48	40.76-103.07
RMSE FB	53.00-57.65	52.90-64.83	64.72-123.15	44.54-49.12	46.13-56.76	57.94-107.32	37.92-41.81	44.94-61.00	61.87-151.58
RMSE pen	11.61-48.12	14.04-158.16	14.09-306.62	11.87-35.43	13.85-141.25	14.02-272.35	13.28-40.77	14.03-145.86	14.10-261.99

C)

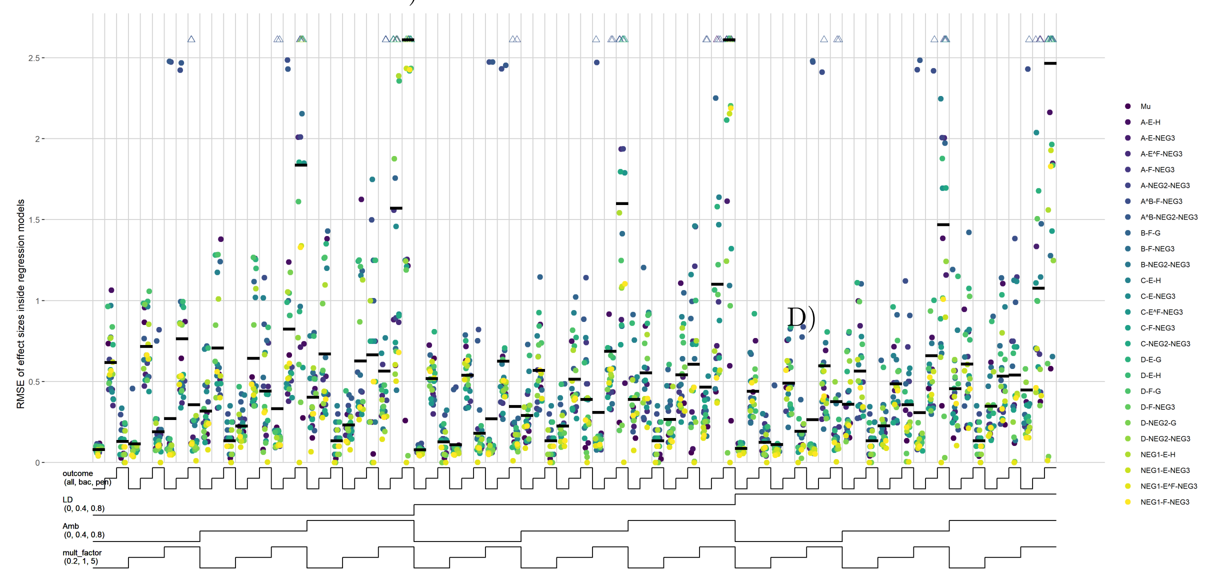

RMSE allele	8.02-27.05	22.48-33.21	23.14-56.50	7.79-26.99	22.52-30.92	26.53-46.57	8.69-26.46	22.67-35.92	34.76-45.55
RMSE FB	61.77-76.39	64.33-82.38	62.76-157.15	51.80-62.52	51.40-68.65	54.14-110.12	43.91-59.77	48.57-66.02	53.33-107.74
RMSE pen	12.96-35.61	13.37-183.71	13.36-323.75	12.71-34.60	13.35-159.93	13.35-284.34	12.45-37.55	13.33-146.96	13.41-246.63

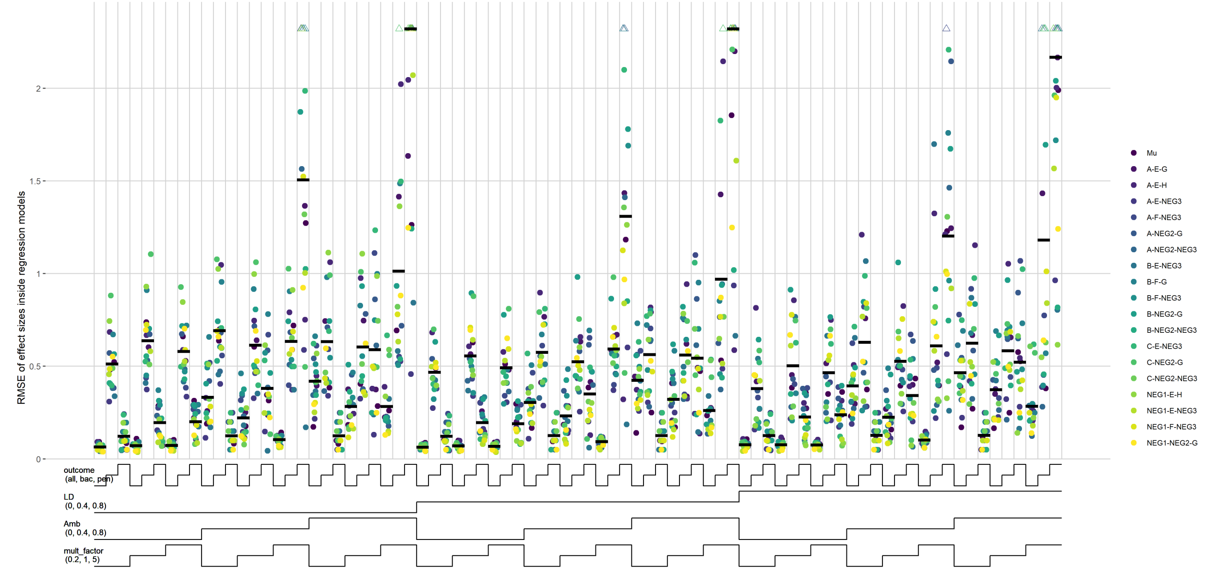

RMSE allele	6.45-7.34	10.51-33.23	28.29-41.93	6.32-7.02	9.39-30.51	26.16-42.43	7.55-7.70	10.16-39.40	28.41-46.61
RMSE FB	51.26-63.87	61.46-69.24	60.45-101.33	46.77-55.66	52.47-59.38	56.11-97.00	37.94-50.36	52.76-63.00	58.35-118.22
RMSE pen	12.29-20.09	12.54-150.67	12.54-270.73	12.26-19.69	12.60-130.97	12.60-242.31	12.60-23.81	12.75-120.32	12.74-216.80

E)

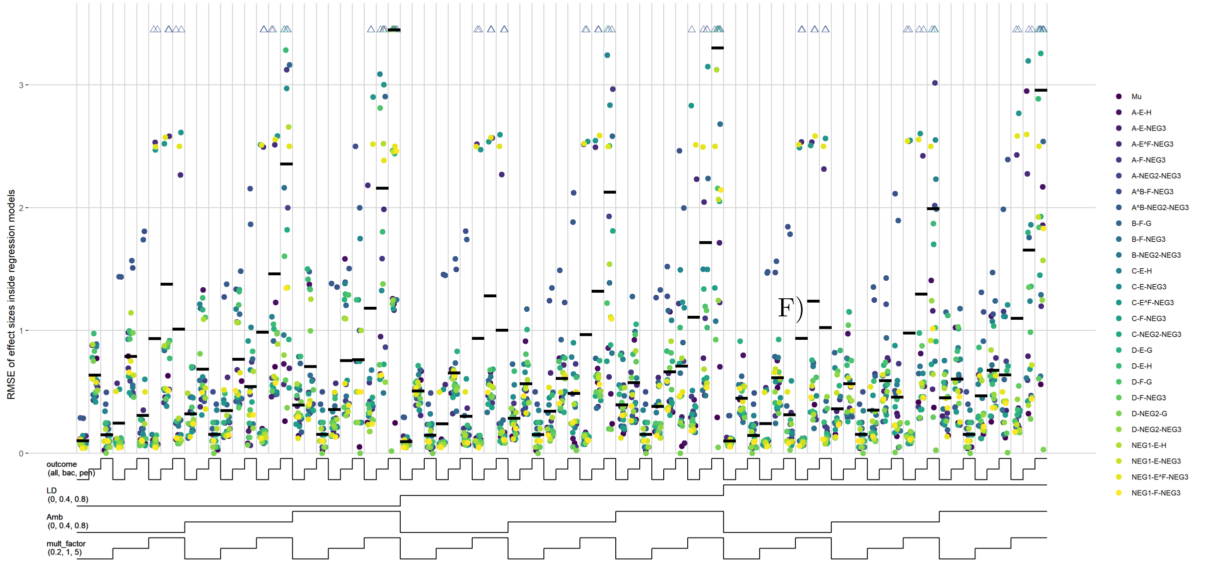

RMSE allele	10.19-93.31	32.07-98.59	35.62-118.09	9.42-93.48	28.41-96.67	38.18-110.86	10.00-93.66	35.01-97.86	45.10-109.82
RMSE FB	63.61-137.74	68.54-146.15	70.54-215.94	50.71-128.20	56.53-131.96	57.57-171.70	44.71-123.86	56.66-129.59	60.39-165.57
RMSE pen	15.03-101.06	15.30-235.70	15.28-372.22	14.63-100.23	15.26-212.72	15.28-330.13	14.46-102.41	15.27-199.26	15.35-295.73

F)

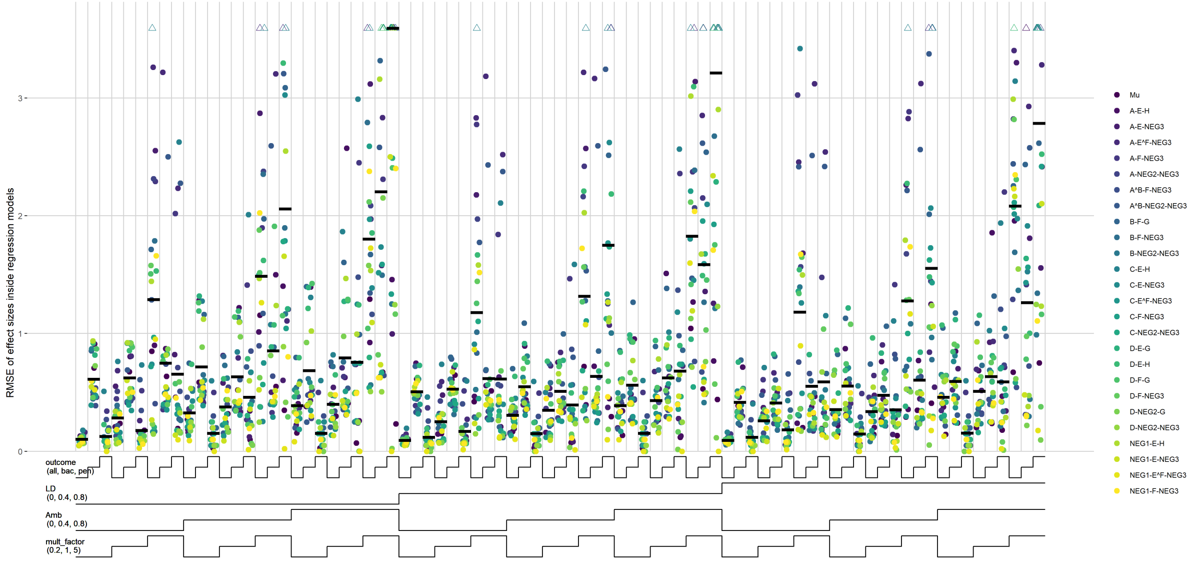

RMSE allele	10.23-128.86	32.52-148.54	38.76-180.25	9.18-117.80	30.59-131.68	38.71-182.58	9.21-118.23	33.75-127.76	45.88-208.15
RMSE FB	61.20-74.80	63.19-85.23	68.53-220.34	50.50-61.68	50.95-63.70	56.15-158.48	41.01-55.13	47.57-60.51	59.39-126.19
RMSE pen	12.65-65.47	15.29-205.70	15.30-369.02	11.88-61.36	15.13-175.03	15.29-321.13	11.88-58.96	14.68-155.33	15.36-278.36

G)

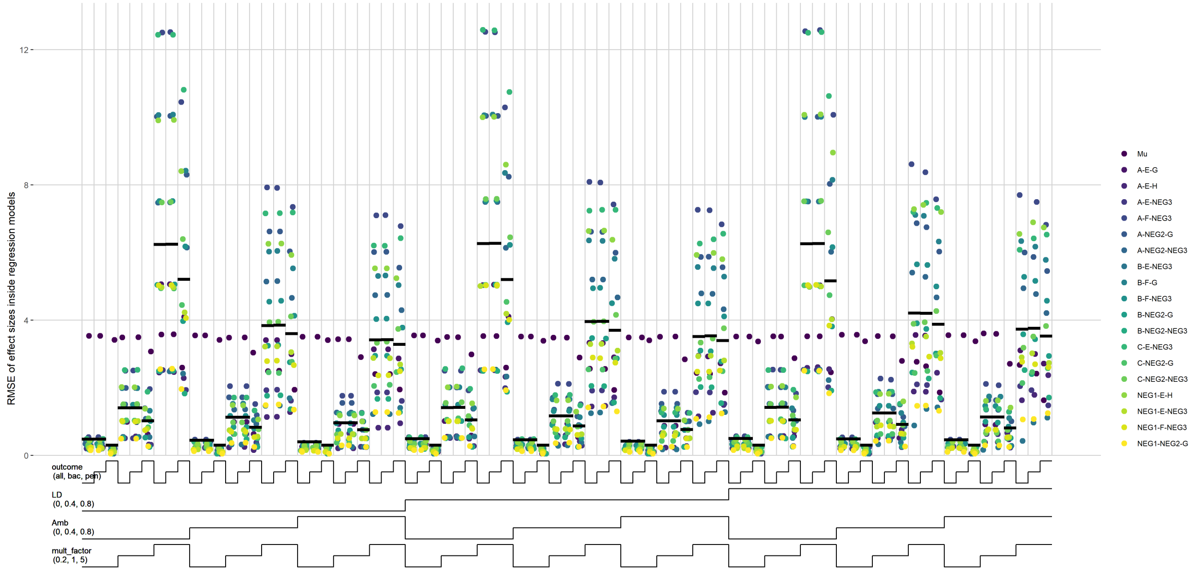

RMSE allele	47.94-624.09	44.73-384.21	40.32-341.61	49.31-626.90	46.23-395.86	41.83-351.24	49.89-625.88	48.47-421.10	45.82-373.38
RMSE FB	48.01-624.81	44.84-385.15	40.32-342.16	49.32-627.36	46.27-396.22	41.83-353.74	49.91-626.69	48.47-420.45	45.82-376.35
RMSE pen	30.08-520.96	30.07-360.18	30.06-328.26	29.99-520.54	29.96-370.14	30.01-339.42	29.83-516.26	29.87-387.63	29.87-352.99

H)

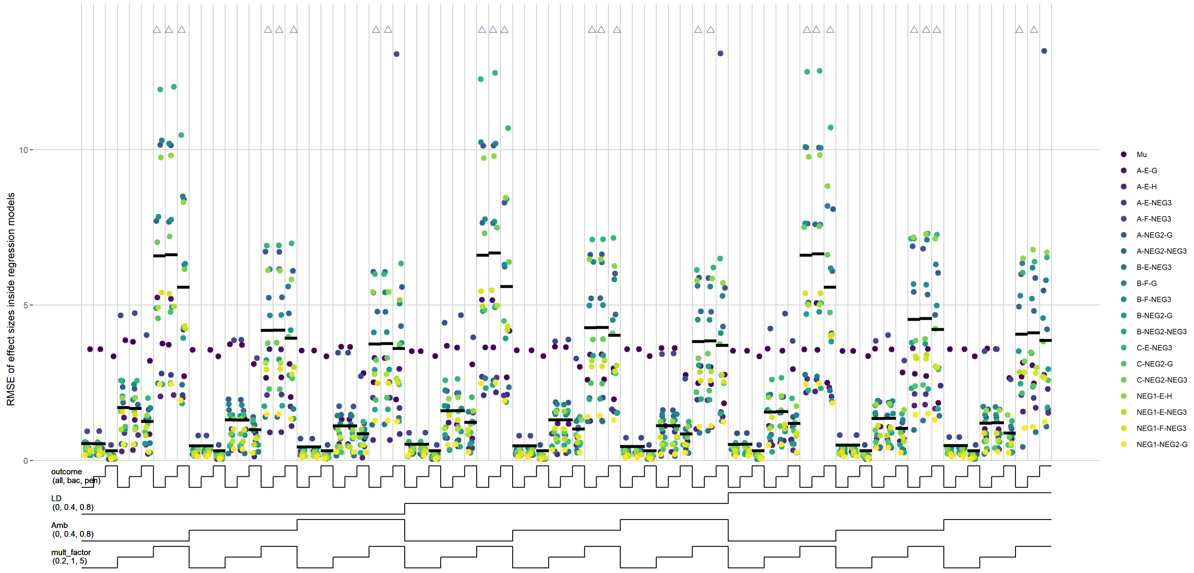

RMSE allele	54.23-658.87	47.35-418.58	43.51-375.24	52.38-661.12	47.12-427.58	45.21-383.21	51.87-661.09	49.05-454.35	47.76-406.66
RMSE FB	53.96-662.06	47.35-419.30	43.51-376.17	52.35-668.43	47.13-428.44	45.21-384.96	51.99-665.33	49.05-457.31	47.76-410.76
RMSE pen	31.06-557.66	31.06-393.47	31.08-360.73	31.10-560.22	31.11-402.70	31.09-370.93	31.08-557.78	31.11-421.64	31.07-386.82

Figure S11: Working outcome model regression coefficient Nested-loop plots of RMSE values for estimated regression coefficients for all sub-scenarios of A) scenario 001 B) scenario 002 C) scenario 011 D) scenario 003 E) scenario 013 F) scenario 012 G) scenario 001* H) scenario 002*. Explanation about the Nested-loop plots is given in the legend of Figure 2. Here only three outcome models are tested: allelic model (lowest line), forward-backward model (middle step) and penalized regression model (upper line). The horizontal bold black lines represent the RMSE of the effect sizes, with its values displayed in the table below the graph. These RMSE values give the range (min-max) for the three sub-scenarios above. Mean RMSE values have been multiplied by 100 for readability.

A)

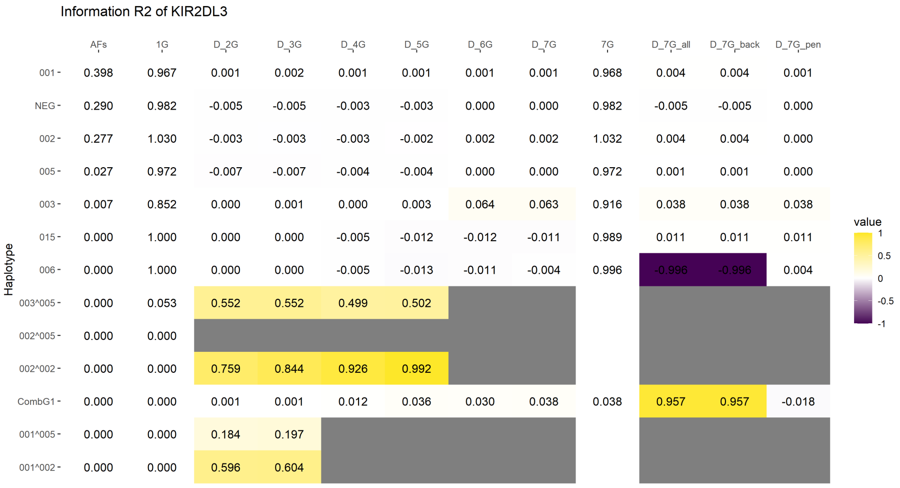
Information R2 of KIR2DL3
AFs 1G D_2G D_3G D_4G D_5G D_6G D_7G 7G D_7G_all D_7G_back D_7G_pen
001 0.398 0.967 0.001 0.002 0.001 0.001 0.001 0.001 0.968 0.004 0.004 0.001
NEG 0.290 0.982 -0.005 -0.005 -0.003 -0.003 0.000 0.000 0.982 -0.005 -0.005 0.000
002 0.277 1.030 -0.003 -0.003 -0.003 -0.002 0.002 0.002 1.032 0.004 0.004 0.000
005 0.027 0.972 -0.007 -0.007 -0.004 -0.004 0.000 0.000 0.972 0.001 0.001 0.000
003 0.007 0.852 0.000 0.001 0.000 0.003 0.064 0.063 0.916 0.038 0.038 0.038
015 0.000 1.000 0.000 0.000 -0.005 -0.012 -0.012 -0.011 0.989 0.011 0.011 0.011
006 0.000 1.000 0.000 0.000 -0.005 -0.013 -0.011 -0.004 0.996 -0.996 -0.996 0.004
003^005 0.000 0.053 0.552 0.552 0.499 0.502
002^005 0.000 0.000
002^002 0.000 0.000 0.759 0.844 0.926 0.992
CombG1 0.000 0.000 0.001 0.001 0.012 0.036 0.030 0.038 0.038 0.957 0.957 -0.018
001^005 0.000 0.000 0.184 0.197
001^002 0.000 0.000 0.596 0.604
Haplotype
value
1
0.5
0
-0.5
-1

B)

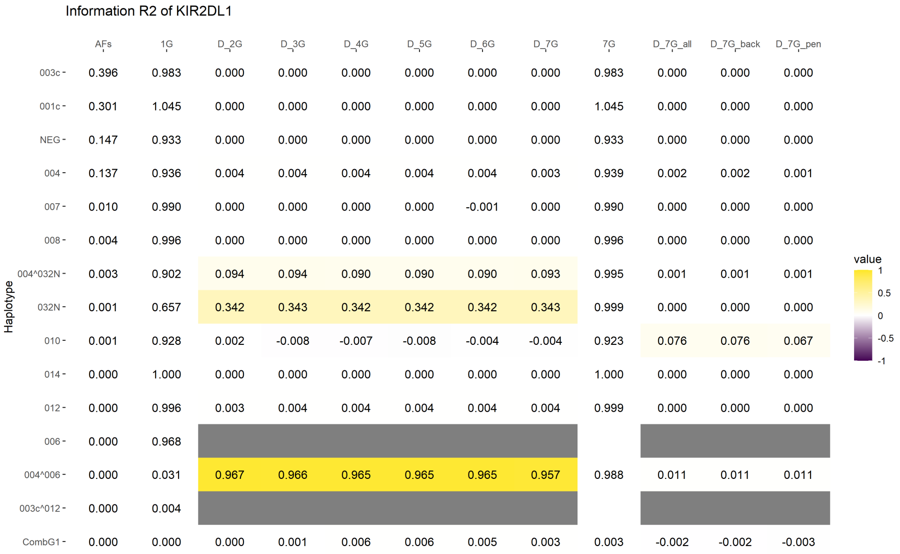
Information R2 of KIR2DL1
AFs 1G D_2G D_3G D_4G D_5G D_6G D_7G 7G D_7G_all D_7G_back D_7G_pen
003c 0.396 0.983 0.000 0.000 0.000 0.000 0.000 0.000 0.983 0.000 0.000 0.000
001c 0.301 1.045 0.000 0.000 0.000 0.000 0.000 0.000 1.045 0.000 0.000 0.000
NEG 0.147 0.933 0.000 0.000 0.000 0.000 0.000 0.000 0.933 0.000 0.000 0.000
004 0.137 0.936 0.004 0.004 0.004 0.004 0.004 0.003 0.939 0.002 0.002 0.001
007 0.010 0.990 0.000 0.000 0.000 0.000 -0.001 0.000 0.990 0.000 0.000 0.000
008 0.004 0.996 0.000 0.000 0.000 0.000 0.000 0.000 0.996 0.000 0.000 0.000
004^032N 0.003 0.902 0.094 0.094 0.090 0.090 0.090 0.093 0.995 0.001 0.001 0.001
032N 0.001 0.657 0.342 0.343 0.342 0.342 0.342 0.343 0.999 0.000 0.000 0.000
010 0.001 0.928 0.002 -0.008 -0.007 -0.008 -0.004 -0.004 0.923 0.076 0.076 0.067
014 0.000 1.000 0.000 0.000 0.000 0.000 0.000 0.000 1.000 0.000 0.000 0.000
012 0.000 0.996 0.003 0.004 0.004 0.004 0.004 0.004 0.999 0.000 0.000 0.000
006 0.000 0.968
004^006 0.000 0.031 0.967 0.966 0.965 0.965 0.965 0.957 0.988 0.011 0.011 0.011
003c^012 0.000 0.004
CombG1 0.000 0.000 0.000 0.001 0.006 0.006 0.005 0.003 0.003 -0.002 -0.002 -0.003
Haplotype
value
1
0.5
0
-0.5
-1

C)

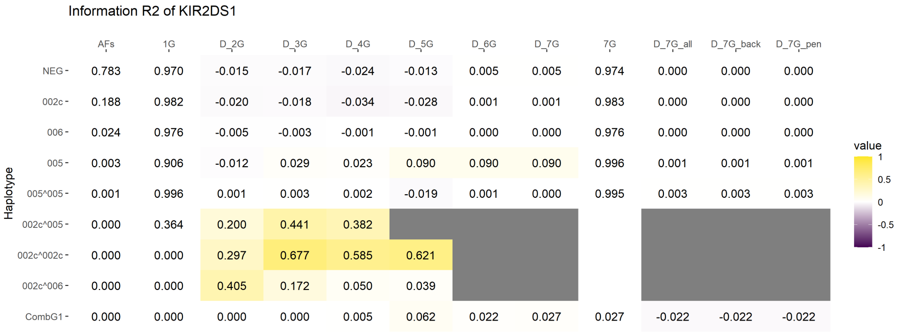
Information R2 of KIR2DS1
AFs 1G D_2G D_3G D_4G D_5G D_6G D_7G 7G D_7G_all D_7G_back D_7G_pen
NEG 0.783 0.970 -0.015 -0.017 -0.024 -0.013 0.005 0.005 0.974 0.000 0.000 0.000
002c 0.188 0.982 -0.020 -0.018 -0.034 -0.028 0.001 0.001 0.983 0.000 0.000 0.000
006 0.024 0.976 -0.005 -0.003 -0.001 -0.001 0.000 0.000 0.976 0.000 0.000 0.000
005 0.003 0.906 -0.012 0.029 0.023 0.090 0.090 0.090 0.996 0.001 0.001 0.001
005^005 0.001 0.996 0.001 0.003 0.002 -0.019 0.001 0.000 0.995 0.003 0.003 0.003
002c^005 0.000 0.364 0.200 0.441 0.382
002c^002c 0.000 0.000 0.297 0.677 0.585 0.621
002c^006 0.000 0.000 0.405 0.172 0.050 0.039
CombG1 0.000 0.000 0.000 0.000 0.005 0.062 0.022 0.027 0.027 -0.022 -0.022 -0.022
Haplotype
value
1
0.5
0
-0.5
-1

D)

Information R2 of KIR3DL1

Haplotype	AFs	1G	D_2G	D_3G	D_4G	D_5G	D_6G	D_7G	7G	D_7G_all	D_7G_back	D_7G_pen
NEG	0.219	0.965	-0.003	0.000	0.001	0.001	0.005	0.005	0.970	0.004	0.004	0.000
001c	0.164	0.964	0.000	0.013	0.013	0.013	0.013	0.013	0.977	0.001	0.001	0.000
004	0.163	0.990	0.000	0.000	0.000	0.000	0.000	0.000	0.990	-0.001	-0.001	0.000
005	0.143	1.054	0.000	0.000	0.000	0.000	0.000	0.000	1.054	0.000	0.000	0.000
002	0.126	0.970	0.000	0.000	-0.001	-0.001	-0.001	-0.001	0.969	-0.001	-0.001	0.001
015c	0.064	1.010	-0.001	0.001	0.002	0.002	0.002	0.002	1.012	0.000	0.000	0.000
008	0.044	0.976	0.000	-0.001	-0.001	-0.001	-0.001	-0.001	0.975	0.002	0.002	0.001
007c	0.022	0.977	-0.016	-0.016	-0.014	-0.014	0.001	0.001	0.978	0.000	0.000	0.000
020	0.019	0.980	0.000	0.000	0.000	0.000	0.000	0.000	0.980	0.000	0.000	0.000
009	0.016	0.979	-0.013	-0.013	-0.010	-0.011	0.005	0.005	0.984	0.000	0.000	0.000
019	0.006	0.994	0.000	0.000	0.000	0.000	0.000	0.000	0.994	0.000	0.000	0.000
053	0.006	0.991	-0.019	-0.019	-0.015	-0.014	0.000	0.000	0.991	0.003	0.003	0.003
001c^001c	0.002	0.795	-0.007	0.191	0.190	0.189	0.189	0.187	0.983	0.016	0.016	0.016
052	0.001	0.999	0.000	0.000	0.000	0.000	0.000	0.000	0.998	-0.998	-0.998	0.000
039	0.001	0.995	-0.015	-0.015	-0.167	-0.167	0.004	0.004	0.999	0.000	0.000	0.000
002^096	0.000	1.000	0.000	0.000	0.000	-0.001	-0.038	-0.042	0.958	-0.958	-0.958	0.041
002^025	0.000	1.000	0.000	0.000	-0.009	-0.010	-0.075	-0.085	0.914	-0.914	-0.914	0.069
004^072	0.000	1.000	0.000	0.000	0.000	0.000	-0.032	-0.035	0.965	-0.965	-0.965	0.034
043	0.000	0.481	0.329	0.336	0.504	0.498	0.518	0.518	0.999	0.000	0.000	0.000
015c^043	0.000	0.770	-0.211	-0.216	-0.741	-0.728						
033^109	0.000	0.250	0.000	0.000	0.000	0.000	-0.007	-0.008	0.242	0.006	0.006	0.006
033^097	0.000	0.250	0.000	0.000	0.000	0.000	-0.007	-0.008	0.242	0.006	0.006	0.006
033^044	0.000	0.250	0.000	0.000	0.000	0.000	-0.007	-0.008	0.242	0.006	0.006	0.006
005^033	0.000	0.250	0.000	0.000	0.000	0.000	-0.007	-0.008	0.242	0.006	0.006	0.006
043^067	0.000	0.105	-0.099	-0.101								
026^043	0.000	0.105	-0.099	-0.101								
016^043	0.000	0.105	-0.099	-0.101								
001c^043	0.000	0.105	-0.099	-0.101								
007c^009	0.000	0.009	0.652	0.654	0.408	0.455						
001c^015c	0.000	0.009	-0.002	-0.008								
002^039	0.000	0.008	0.032	0.032	0.548	0.554						
005^039	0.000	0.001										
007c^053	0.000	0.000	0.384	0.383	0.230	0.207						
CombG1	0.000	0.000	0.000	0.000	0.002	0.002	0.040	0.043	0.043	1.178	1.178	-0.031

value: 1, 0.5, 0, -0.5, -1

E)

Information R2 of KIR3DS1

Haplotype	AFs	1G	D_2G	D_3G	D_4G	D_5G	D_6G	D_7G	7G	D_7G_all	D_7G_back	D_7G_pen
NEG	0.775	1.002	-0.048	-0.050	-0.047	-0.047	-0.038	-0.037	0.965	0.001	0.001	0.000
013c	0.214	0.997	-0.065	-0.059	-0.053	-0.046	-0.034	-0.034	0.963	0.003	0.003	0.003
049N	0.010	0.970	-0.021	-0.027	-0.014	0.003	0.020	0.020	0.990	0.000	0.000	0.000
013c^049N	0.000	0.074	0.162	0.172	0.107	0.011						
013c^013c	0.000	0.000	0.599	0.720	0.790	0.900	0.966	0.961	0.961	0.032	0.032	0.029
CombG1	0.000	0.000	0.000	0.000	0.000	0.109	0.003	0.003	0.003	-0.003	-0.003	-0.003

value: 1, 0.5, 0, -0.5, -1

F)

Information R2 of KIR2DL2

Haplotype	AFs	1G	D_2G	D_3G	D_4G	D_5G	D_6G	D_7G	7G	D_7G_all	D_7G_back	D_7G_pen
NEG	0.714	0.998	-0.065	-0.065	-0.045	-0.036	-0.007	-0.007	0.991	-0.014	0.006	0.003
001	0.171	1.005	-0.119	-0.119	-0.086	-0.061	-0.003	-0.003	1.002	-0.027	-0.007	0.000
003	0.115	0.975	-0.011	-0.011	-0.006	-0.007	0.000	0.001	0.976	-0.009	0.000	0.000
001^003	0.000	0.000	0.174	0.170	0.421	0.419	0.984	0.997	0.997	0.002	0.002	0.002
001^001	0.000	0.000	0.561	0.561	0.425	0.346	0.991	0.994	0.994	0.005	0.005	0.005
CombG1	0.000	0.000	0.001	0.001	0.051	0.051	0.025	0.018	0.018	0.980	0.977	0.934

value: 1, 0.5, 0, -0.5, -1

G)

Information R2 of KIR2DS4

Haplotype	AFs	1G	D_2G	D_3G	D_4G	D_5G	D_6G	D_7G	7G	D_7G_all	D_7G_back	D_7G_pen
003	0.229	0.990	0.000	0.000	0.000	0.000	0.000	0.000	0.990	0.000	0.000	0.000
NEG	0.215	0.979	-0.001	-0.001	-0.002	-0.002	-0.001	-0.001	0.978	0.000	0.000	0.000
001c	0.212	0.969	0.000	0.000	0.000	0.000	0.000	0.000	0.969	0.000	0.000	0.000
006	0.171	0.985	0.001	0.001	0.001	0.001	0.001	0.001	0.985	0.000	0.000	0.000
010	0.150	1.066	0.000	0.000	0.000	-0.001	0.000	0.000	1.066	0.000	0.000	0.000
004	0.022	0.977	0.000	0.000	0.000	0.000	0.000	0.000	0.977	0.000	0.000	0.000
001c^003	0.000	0.000										
010^010	0.000	0.000										
004^006	0.000	0.000	0.999	0.999	0.999	0.999	0.922	0.918	0.918	0.081	0.081	0.081
CombG1	0.000	0.000	0.000	0.001	0.003	0.003	0.035	0.042	0.042	-0.042	-0.042	-0.042

value: 1, 0.5, 0, -0.5, -1

Table S2: Information R^2 for the seven starting genes. A) *KIR2DL3* B) *KIR2DL1* C) *KIR2DS1* D) *KIR3DL1* E) *KIR3DS1* F) *KIR2DL2* G) *KIR2DS4*. Column 1 shows the HTFs estimated in the one-gene analysis where the amount of information in this analysis is denoted in column 2. The differences in the amount of information with an increasing number of genes in the combination, compared to this one-gene analysis, are denoted in columns 3-8. The amount of information in the seven-gene analysis of the baseline no-outcome EM-algorithm model is denoted in column 9. The color spectrum indicates whether the amount of information increases (green) or decreases (red) in the combination with respect to the one gene analysis (for column 3-8) or to the seven gene baseline analysis for the three outcome models (in column 10-12). Completely white boxes indicate that there is no difference, where gray boxes indicate that the corresponding haplotype is completely collapsed.
